# Supplementary material for: Growth patterns in childhood and adolescence and adult body composition: a pooled analysis of birth cohort studies from five low and middle-income countries (COHORTS collaboration)
Source: BMJ Open. 2023 Mar 14;13(3):e068427. doi: 10.1136/bmjopen-2022-068427 (PMC10030655; doi:10.1136/bmjopen-2022-068427)
Supplement: Supplementary data [file bmjopen-2022-068427supp001.pdf]

Supplementary Tables

Supplementary Table 1. Data missing patterns (covariates) of analytic samples at each study site

Covariates	Brazil 1982 (n=674)	Brazil 1993 (n=827)	Guatemala (n=163)	India (n=681)	The Philippines (n=1197)	South Africa (n=595)
Sex	0	0	0	0	0	0
Gestational age	137 (20.3%)	91 (11%)	10 (6.1%)	149	8 (0.7%)	4 (0.7%)
Birth order	0	0	0	256 (37.6%)	0	0
Maternal age at first child birth	0	0	0	227 (33.3%)	0	0
Maternal height	11 (1.6%)	8 (1%)	0	246 (36.1%)	0	84 (14.1%)
Maternal schooling	1 (0.1%)	12 (1.4%)	1 (0.6%)	62	0	24 (4.0%)
Maternal marital status	0	0	0	0	0	0
Paternal age at child birth	163 (24.2%)	NA	3 (1.8%)	NA	36 (3.0%)	NA
Paternal schooling	40 (5.9%)	48 (5.8%)	3 (1.8%)	116 (17.0%)	59 (4.9%)	158 (26.5%)
Income in childhood	7 (1.0%)	6 (0.7%)	NA	222 (32.6%)	5 (0.4%)	244 (41.0%)
Wealth index in childhood	0	NA	1 (0.6%)	222 (32.6%)	0	25 (4.2%)
Age at menarche (women)	0	NA	7 (4.3%)	52 (7.6%)	1 (0.1%)	4 (0.7%)
Teenage childbearing	12 (1.8%)	24 (2.9%)	24 (14.7%)	280 (41.1%)	253 (21.1%)	NA
Skin color Brazil	0	0	NA	NA	NA	NA

NA: not available variable

Supplementary Table 2. Characteristics of participants excluded (missing anthropometric and body composition data) and included in the analyses at each study site

	Brazil 82		Brazil 93		Guatemala		India		The Philippines		South Africa	
	Excluded (N=5239)	Analytic sample (N=674)	Excluded (N=4421)	Analytic sample (N=827)	Excluded (N=2229)	Analytic sample (N=163)	Excluded (N=6849)	Analytic sample (N=681)	Excluded (N=1883)	Analytic sample (N=1197)	Excluded (N=2678)	Analytic sample (N=595)
Birth Variables												
Sex												
n	5239	674	4421	827	2229	163	6849	681	1883	1197	2678	595
Women	48.6%	49.0%	49.6%	54.7%*	47.8%	58.9%*	48.9%	37.4%*	48.2%	45.2%	51.3%	51.8%
Gestational age (weeks)												
n	4063	537	3936	736	698	153	847	532	1859	1189	2580	591
Mean (SD)	39.3 (1.9)	39.5 (1.8)	39.5 (2.3)	39.3 (2.5)	39.3 (2.9)	39.2 (3.4)	38.9 (2.7)	38.9 (2.4)	38.7 (2.2)	38.8 (2.1)	38.2 (1.9)	37.9* (2.0)
Birth length (cm)												
n	NA	NA	4335	827	689	163	5964	681	1853	1197	NA	NA
Mean (SD)	NA	NA	48.9 (2.3)	48.1* (2.6)	50.0 (2.3)	49.0*(2.4)	48.2 (2.3)	48.4 (2.1)	49.1 (2.1)	49.1 (2.0)	NA	NA
Birth weight (kg)												
n	5131	674	4405	827	810	163	6128	681	1832	1197	2672	595
Mean (SD)	3.2 (0.6)	3.3* (0.6)	3.2 (0.5)	3.0* (0.6)	3.1 (0.5)	3.0 (0.5)	2.8 (0.4)	2.8 (0.4)	3.0 (0.5)	3.0 (0.4)	3.1 (0.5)	3.1 (0.5)
Childhood variables												
HAZ in infancy												
n	4162	674	536	827	922	163	4661	681	1307	1197	1210	595
Mean (SD)	-0.7 (1.2)	-0.5*(1.2)	-0.3 (1.6)	-0.3 (1.3)	-2.9 (1.2)	-2.9 (1.1)	-1.9 (1.2)	-2.0 (1.1)	-2.4 (1.2)	-2.4 (1.1)	-1.1 (1.2)	-1.2 (1.0)
HAZ in childhood												
n	3978	674	440	827	951	163	4151	681	1064	1197	1209	595
Mean (SD)	-0.7 (1.1)	-0.6 (1.1)	-0.3 (1.3)	-0.3 (1.1)	-2.4 (1.0)	-2.3 (0.9)	-1.9 (1.1)	-2.0 (1.0)	-2.0 (1.0)	-2.1 (0.9)	-0.9 (0.9)	-1.0 (0.9)
HAZ in adolescence												
n	398	674	3172	827	724	163	2384	681	891	1197	1328	595
Mean (SD)	-0.3 (1.0)	-0.2 (1.0)	-0.1 (1.0)	-0.3* (1.0)	-2.0 (0.9)	-1.9 (0.8)	-1.1 (1.0)	-1.2 (1.0)	-1.8 (0.9)	-1.9* (0.8)	-0.6 (1.0)	-0.6 (0.9)
WAZ in infancy												
n	4163	674	534	827	922	163	4623	681	1307	1197	1596	595
Mean (SD)	0.1 (1.1)	0.2 (1.1)	0.2 (1.4)	0.3 (1.1)	-1.7 (1.0)	-1.7(1.0)	-1.4 (1.1)	-1.5 (1.1)	-1.7 (1.1)	-1.7 (0.9)	-0.4 (1.0)	-0.5 (1.0)
WAZ in childhood												
n	3978	674	444	827	950	163	4127	681	1064	1197	1604	595
Mean (SD)	-0.0 (1.1)	0.1 (1.0)	0.5 (2.9)	0.1 (1.2)*	-1.4 (0.8)	-1.4(0.8)	-1.3 (1.0)	-1.4 (0.9)	-1.8 (1.1)	-2.0 (0.9)*	-0.4 (0.9)	-0.5 (0.9)*
BAZ in adolescence												
n	398	674	3172	827	723	163	2384	681	891	1197	1323	595
Mean (SD)	0.3 (1.1)	0.3 (1.1)	0.4 (1.2)	0.3* (1.1)	-0.5 (0.8)	-0.3 (0.9)	-0.8 (1.2)	-1.0* (1.2)	-0.8 (1.1)	-0.9 (1.0)	-0.1 (1.3)	-0.1 (1.2)

Adult variables												
Age (years)												
n	2989	667	2986	827	1115	153	149	681	129	1197	913	484
Mean (SD)	30.2 (0.3)	30.2 (0.3)	22.6 (0.3)	22.6 (0.3)	47.7 (4.4)	45.5*(1.1)	46.1 (1.2)	46.1 (1.1)	34.5 (0.5)	34.4 (0.5)	22.0 (0.4)	22.0 (0.4)
Height (cm)												
n	2933	674	2765	827	982	163	140	681	129	1197	921	595
Mean (SD)	167.5(9.2)	168.6*(9.3)	167.8(9.4)	166.4*(10.0)	156.3(8.3)	157.3(8.2)	164.7(9.1)	164.1(9.8)	152.6(26.1)	157.4*(8.1)	165.7(8.9)	165.4(8.6)
Weight (kg)												
n	2877	674	2764	827	982	163	136	681	126	1197	911	595
Mean (SD)	75.6(17.7)	76.6(17.6)	71.9(16.8)	69.6*(16.4)	68.7(13.2)	70.2(12.9)	76.5(14.5)	76.6(15.0)	62.1(14.9)	62.1 (13.1)	64.8(14.8)	63.8(13.3)
BMI (kg/m^2)												
n	2863	671	2735	827	982	163	135	681	107	1197	911	595
Mean (SD)	26.8 (5.5)	26.9 (5.5)	25.3 (5.3)	25.0 (5.4)	28.1 (5.1)	28.5 (5.1)	28.4 (5.5)	28.4 (4.8)	24.8 (5.0)	25.0 (4.6)	23.7 (5.7)	23.4 (5.1)
Waist circumference (cm)												
n	2893	674	2766	827	982	163	138	681	103	1197	902	595
Mean (SD)	84.7(12.6)	85.4(12.5)	80.2(11.6)	79.2 (11.5)	98.7(12.4)	99.1(11.6)	97.8(12.1)	97.3(12.2)	81.1(12.6)	81.5(11.1)	78.4(11.8)	79.2(11.8)
FMI, kg/m^2												
n	2849	674	2735	827	939	163	112	681	90	1197	452	595
Mean (SD)	8.7 (4.6)	8.8 (4.5)	7.7 (4.5)	7.5 (4.6)	10.7 (4.1)	10.7(3.9)	8.6 (3.7)	9.1 (3.6)	6.7 (3.4)	7.1 (3.5)	6.7 (4.3)	6.9 (4.2)
FFMI, kg/m^2												
n	2849	674	2735	827	941	163	112	681	90	1197	452	595
Mean (SD)	18.1 (2.7)	18.1 (2.7)	17.5 (2.6)	17.3 (2.5)	17.5 (2.4)	17.8 (2.5)	19.5 (2.6)	19.3 (2.4)	17.6 (2.2)	17.9 (2.3)	14.8 (2.1)	14.6 (2.0)

*p<0.01 based on Bonferroni-adjusted comparisons of participants excluded and included in the analytic sample at each study site

Supplementary Table 3. Characteristics at birth, childhood and parental of study participants, stratified by study site and sex[1]

	Brazil 1982		Brazil 1993		Guatemala		India		The Philippines		South Africa	
	Men (n=344)	Women (n=330)	Men (n=375)	Women (n=452)	Men (n=67)	Women (n=96)	Men (n=426)	Women (n=255)	Men (n=656)	Women (n=541)	Men (n=287)	Women (n=308)
**Birth variables**												
Gestational age (weeks)	39.4 (1.8)	39.5 (1.8)	39.6 (2.2)	39.7 (2.3)	39.3 (3.0)	39.1 (3.6)	38.8 (2.4)	39.1 (2.3)	38.7 (2.1)	38.8 (2.1)	38.0 (1.9)	37.8 (2.0)
Birth order (%)												
1	143 (41.6%)	120 (36.4%)	147 (37.1%)	145 (33.1%)	12 (17.9%)	14 (14.6%)	46 (17.2%)	26 (16.5%)	149 (22.7%)	120 (22.2%)	105 (36.6%)	118 (38.3%)
2	98 (28.5%)	85 (25.8%)	107 (27.2%)	130 (29.6%)	13 (19.4%)	12 (12.5%)	59 (22.1%)	37 (23.4%)	132 (20.1%)	120 (22.2%)	86 (30.0%)	93 (30.2%)
3	54 (15.7%)	64 (19.4%)	63 (15.8%)	99 (22.6%)	6 (9.0%)	15 (15.6%)	53 (19.9%)	31 (19.6%)	130 (19.8%)	103 (19.0%)	48 (16.7%)	51 (16.6%)
≥4	49 (14.2%)	61 (18.5%)	79 (20.0%)	64 (14.6%)	36 (53.7%)	55 (57.3%)	109 (40.8%)	64 (40.5%)	245 (37.3%)	198 (36.6%)	48 (16.7%)	46 (14.9%)
**Childhood variables[2]**												
HAZ in infancy	-0.6 (1.2)	-0.5 (1.2)	-0.1 (1.3)	-0.0 (1.2)	-3.1 (1.2)	-2.8 (1.0)	-2.0 (1.1)	-1.9 (1.1)	-2.5 (1.1)	-2.4 (1.1)	-1.4 (1.0)	-1.1 (1.0)
HAZ in childhood	-0.5 (1.1)	-0.6 (1.1)	-0.1 (1.2)	-0.2 (1.1)	-2.3 (0.9)	-2.4 (0.9)	-1.9 (1.0)	-2.1 (1.0)	-2.1 (0.9)	-2.1 (0.9)	-1.0 (0.9)	-0.9 (0.9)
HAZ in adolescence	-0.1 (1.0)	-0.3 (0.9)	0.5 (1.2)	0.5 (1.0)	-2.0 (0.9)	-1.9 (0.7)	-1.2 (1.1)	-1.3 (0.8)	-1.9 (0.8)	-1.9 (0.8)	-0.7 (1.0)	-0.5 (0.9)
WAZ at infancy	0.2 (1.1)	0.2 (0.1)	0.3 (1.2)	0.2 (1.1)	-1.9 (1.0)	-1.6 (1.0)	-1.5 (1.1)	-1.5 (1.0)	-1.7 (0.9)	-1.7 (1.0)	-0.7 (1.1)	-0.4 (0.9)
WAZ in childhood	0.2 (1.0)	0.0 (1.0)	0.6 (1.2)	0.4 (1.1)	-1.4 (0.7)	-1.4 (0.8)	-1.3 (0.9)	-1.5 (0.9)	-2.0 (1.0)	-1.9 (0.9)	-0.5 (0.9)	-0.5 (0.9)
BAZ in adolescence	0.3 (1.1)	0.3 (1.1)	0.3 (1.2)	0.3 (1.1)	-0.9 (0.8)	0.1 (0.7)	-1.3 (1.2)	-0.6 (1.0)	-1.0 (1.0)	-0.7 (1.0)	-0.5 (1.1)	0.2 (1.2)
**Parental variables**												
Maternal age at childbirth (years)	26.5 (6.4)	26.8 (6.5)	26.3 (6.5)	26.4 (6.3)	26.7 (7.9)	27.7 (7.2)	26.9 (5.9)	26.9 (5.5)	26.6 (6.2)	26.4 (6.0)	26.0 (6.5)	25.7 (6.4)
Maternal height (cm)	157.1(6.4)	156.7(6.2)	160.2(6.5)	160.0(6.9)	148.3(4.7)	148.7(5.1)	151.9(5.8)	151.8(5.1)	150.4(4.8)	150.2(5.0)	158.2(6.4)	158.6(6.0)
Maternal schooling (years)	6.7 (4.1)	6.8 (4.3)	7.0 (3.5)	6.7 (3.4)	1.3 (1.5)	1.3 (1.5)	5.7 (4.5)	5.6 (4.4)	6.9 (3.3)	6.7 (3.1)	9.6 (2.5)	9.7 (2.7)
Paternal age (years)	29.8 (7.0)	30.3 (7.0)	NA	NA	32.7 (9.4)	32.9 (8.4)	NA	NA	29.1 (6.8)	28.9 (6.8)	NA	NA
Paternal schooling (years)	6.9 (4.3)	7.1 (4.0)	6.8 (3.6)	6.8 (3.4)	2.0 (2.0)	1.8 (2.0)	10.4 (5.0)	11.4 (4.7)	7.0 (3.5)	6.7 (3.2)	10.6 (2.2)	10.7 (2.6)

[1] Values are means (SD) or n (percentages). Sample sizes (n) refer to participants with non-missing data for outcomes (adult body composition), and non-missing data for exposures (growth measures). NA, not available data.[2] HAZ, height-for-age Z-score; WAZ, weight-for-age Z-score; BAZ, BMI-for-age Z-score; BMI, body mass index. HAZ and WAZ measures in infancy were measured at 24 months of age in all sites except Brazil 1993, where children were measured at 12 months of age. Childhood corresponds to 48 months for all study sites but Philippines, where anthropometric measures were obtained at 102 months of age. Measures of adolescence were obtained at 15 years of age (180 months) in all study sites.

Supplementary Table 4. Adjusted associations between weight at birth, conditional growth (height and relative weight) in infancy, childhood, and adolescence with adult fat mass index (FMI, SD units), stratified by study site and sex[1]

	Brazil 1982		Brazil 1993		Guatemala		India		The Philippines		South Africa	
	Men (n = 344)	Women (n = 330)	Men (n = 375)	Women (n = 452)	Men (n = 67)	Women (n = 96)	Men (n = 426)	Women (n = 255)	Men (n = 656)	Women (n = 541)	Men (n = 287)	Women (n = 308)
**Conditional growth, z-scores[2]**												
Birth weight	0.04 (-0.03,0.11)	-0.02 (-0.1,0.07)	0.02 (-0.05,0.09)	0.11* (0.04,0.17)	0.10 (-0.18,0.38)	0.21 (-0.03,0.44)	0.15* (0.05,0.25)	0.18* (0.04,0.32)	0.10* (0.03,0.18)	0.12* (0.04,0.20)	-0.01 (-0.11,0.09)	0.02 (-0.07,0.11)
Conditional relative weight in infancy	0.18* (0.08,0.28)	0.32* (0.17,0.46)	0.22* (0.12,0.32)	0.23* (0.14,0.32)	0.47 (-0.09,1.04)	-0.06 (-0.52,0.41)	0.20* (0.06,0.34)	0.25* (0.07,0.44)	0.25* (0.15,0.36)	0.33* (0.21,0.45)	0.22* (0.12,0.31)	0.24* (0.12,0.36)
Conditional relative weight in childhood	0.39* (0.24,0.55)	0.49* (0.29,0.68)	0.32* (0.22,0.42)	0.50* (0.40,0.61)	0.02 (-0.83,0.87)	0.43 (-0.18,1.05)	0.36* (0.15,0.56)	0.04 (-0.22,0.3)	0.59* (0.46,0.72)	0.72* (0.57,0.88)	0.28* (0.12,0.45)	0.29* (0.11,0.46)
Conditional relative weight in adolescence	0.43* (0.35,0.51)	0.61* (0.52,0.71)	0.38* (0.30,0.45)	0.56* (0.45,0.66)	0.19 (-0.32,0.71)	0.53* (0.13,0.94)	0.28* (0.19,0.37)	0.50* (0.35,0.66)	0.38* (0.29,0.47)	0.42* (0.32,0.52)	0.56* (0.47,0.66)	0.58* (0.49,0.67)
Conditional height in infancy	0.01 (-0.06,0.09)	0.17* (0.07,0.27)	0.05* (0.00,0.11)	0.06 (-0.01,0.13)	0.27 (-0.06,0.61)	0.10 (-0.19,0.39)	0.09 (0.00,0.19)	0.12 (-0.03,0.28)	0.12* (0.05,0.19)	0.14* (0.06,0.21)	0.06 (-0.03,0.16)	0.10* (0.01,0.19)
Conditional height in childhood	-0.07 (-0.20,0.07)	0.00 (-0.17,0.17)	0.13* (0.05,0.21)	0.14* (0.03,0.24)	-0.12 (-0.59,0.35)	0.00 (-0.62,0.62)	0.10 (-0.08,0.27)	0.12 (-0.12,0.36)	0.13* (0.02,0.24)	0.25* (0.13,0.36)	0.09 (-0.09,0.26)	0.16 (0,0.33)
Conditional height in adolescence	0.07 (-0.04,0.17)	0.22* (0.07,0.37)	0.08 (-0.01,0.16)	0.03 (-0.09,0.15)	0.38 (-0.1,0.86)	-0.06 (-0.65,0.53)	0.00 (-0.12,0.12)	0.03 (-0.25,0.31)	0.00 (-0.15,0.14)	-0.10 (-0.28,0.08)	-0.1 (-0.25,0.04)	-0.08 (-0.23,0.08)

[1] Values are standardized linear regression coefficients (βs and 95% CIs). In adjusted analyses we controlled for birth characteristics (gestational age, birth order), maternal characteristics (height, age at childbirth, schooling, marital status), paternal characteristics (age, schooling), income and/or wealth index of child's household at birth, and age in adulthood. In women' models, we adjusted for age of menarche and teenage childbearing. Additionally, we controlled for maternal skin color in both Brazilian cohorts and adjusted for birth year and intervention group (village fixed effects) in Guatemala analysis. * P value < 0.05; SD = Standard Deviation

[2] We used birth weight as the anchor in all conditional models given that this measure was available across all study sites

Supplementary Table 5. Adjusted associations between weight at birth, conditional growth (height and relative weight) in infancy, childhood, and adolescence with adult fat-free mass index (FFMI, SD units), stratified by study site and sex[1]

	**Brazil 1982**		**Brazil 1993**		**Guatemala**		**India**		**The Philippines**		**South Africa**	
	Men (n = 344)	Women (n = 330)	Men (n = 375)	Women (n = 452)	Men (n = 67)	Women (n = 96)	Men (n = 426)	Women (n = 255)	Men (n = 656)	Women (n = 541)	Men (n = 287)	Women (n = 308)
**Conditional growth, z-scores[2]**												
Birth weight	0.11** (0.04,0.17)	0.07* (0.01,0.12)	0.05* (0.00,0.10)	0.10* (0.05,0.14)	-0.06 (-0.31,0.19)	0.41* (0.21,0.62)	0.12* (0.02,0.21)	0.12 (-0.01,0.25)	0.13* (0.06,0.21)	0.10* (0.03,0.18)	0.20* (0.10,0.30)	0.15* (0.06,0.23)
Conditional relative weight in infancy	0.3*** (0.2,0.39)	0.2*** (0.11,0.29)	0.30* (0.22,0.37)	0.29* (0.22,0.36)	0.45 (-0.05,0.95)	0.23 (-0.18,0.64)	0.34* (0.21,0.46)	0.40* (0.23,0.57)	0.31* (0.21,0.41)	0.37* (0.26,0.49)	0.18* (0.09,0.28)	0.21* (0.09,0.32)
Conditional relative weight in childhood	0.41*** (0.27,0.55)	0.4*** (0.27,0.52)	0.43* (0.33,0.52)	0.41* (0.33,0.49)	0.33 (-0.42,1.08)	0.20 (-0.34,0.74)	0.46* (0.26,0.65)	0.37* (0.13,0.61)	0.71* (0.59,0.83)	0.71* (0.56,0.86)	0.31* (0.15,0.46)	0.41* (0.24,0.58)
Conditional relative weight in adolescence	0.39*** (0.31,0.46)	0.39*** (0.33,0.45)	0.35* (0.28,0.41)	0.38* (0.32,0.44)	0.58* (0.12,1.03)	0.45* (0.10,0.81)	0.36* (0.28,0.44)	0.52* (0.38,0.67)	0.47* (0.39,0.55)	0.47* (0.38,0.57)	0.60* (0.51,0.69)	0.62* (0.53,0.71)
Conditional height in infancy	0.05 (-0.02,0.12)	0.05. (-0.01,0.12)	-0.01 (-0.07,0.05)	0.06* (0.02,0.11)	0.35* (0.06,0.64)	0.16 (-0.10,0.41)	0.13* (0.04,0.23)	-0.05 (-0.19,0.10)	0.14* (0.07,0.20)	0.17* (0.09,0.24)	0.05 (-0.04,0.14)	0.20* (0.11,0.29)
Conditional height in childhood	-0.08 (-0.2,0.04)	-0.04 (-0.15,0.07)	0.03 (-0.06,0.12)	0.06 (0.00,0.12)	-0.11 (-0.53,0.3)	-0.39 (-0.94,0.15)	0.11 (-0.05,0.28)	-0.14 (-0.36,0.08)	0.16* (0.06,0.27)	0.24* (0.13,0.35)	-0.04 (-0.2,0.12)	0.06 (-0.1,0.22)
Conditional height in adolescence	0.02 (-0.08,0.11)	-0.01 (-0.1,0.09)	-0.08 (-0.18,0.02)	0.08 (-0.01,0.17)	0.23 (-0.19,0.66)	-0.04 (-0.56,0.48)	0.08 (-0.03,0.19)	-0.12 (-0.38,0.14)	-0.04 (-0.17,0.1)	-0.11 (-0.29,0.06)	-0.02 (-0.16,0.12)	0.13 (-0.01,0.28)

[1] Values are standardized linear regression coefficients (βs and 95% CIs). In adjusted analyses we controlled for birth characteristics (gestational age, birth order), maternal characteristics (height, age at childbirth, schooling, marital status), paternal characteristics (age, schooling), income and/or wealth index of child's household at birth, and age in adulthood. In women' models, we adjusted for age of menarche and teenage childbearing. Additionally, we controlled for maternal skin color in both Brazilian cohorts and adjusted for birth year and intervention group (village fixed effects) in Guatemala analysis. * P value < 0.05; SD = Standard Deviation

[2] We used birth weight as the anchor in all conditional models given that this measure was available across all study sites

Supplementary Table 6. Adjusted associations between weight at birth, conditional growth (height and relative weight) in infancy, childhood, and adolescence with adult fat mass/fat free mass ratio (FM/FFM, SD units), stratified by study site and sex[1]

	Brazil 1982		Brazil 1993		Guatemala		India		The Philippines		South Africa	
	Men (n = 344)	Women (n = 330)	Men (n = 375)	Women (n = 452)	Men (n = 67)	Women (n = 96)	Men (n = 426)	Women (n = 255)	Men (n = 656)	Women (n = 541)	Men (n = 287)	Women (n = 308)
**Conditional growth, z-scores[2]**												
Birth weight	0.01 (-0.05,0.07)	-0.05 (-0.14,0.03)	0.01 (-0.05,0.07)	0.07* (0.01,0.13)	0.18 (-0.12,0.47)	0.02 (-0.24,0.27)	0.15* (0.05,0.26)	0.18* (0.04,0.33)	0.08* (0.01,0.16)	0.12* (0.04,0.20)	-0.07 (-0.18,0.04)	-0.03 (-0.13,0.07)
Conditional relative weight in infancy	0.11* (0.02,0.2)	0.25* (0.11,0.39)	0.15* (0.06,0.24)	0.14* (0.05,0.24)	0.35 (-0.24,0.94)	-0.24 (-0.74,0.25)	0.13 (-0.01,0.27)	0.16 (-0.03,0.35)	0.21* (0.10,0.31)	0.32* (0.20,0.44)	0.20* (0.09,0.30)	0.21* (0.08,0.34)
Conditional relative weight in childhood	0.26* (0.13,0.4)	0.38* (0.19,0.57)	0.22* (0.13,0.3)	0.39* (0.28,0.49)	-0.16 (-1.05,0.72)	0.31 (-0.35,0.96)	0.26* (0.04,0.47)	-0.11 (-0.38,0.15)	0.52* (0.38,0.65)	0.69* (0.53,0.85)	0.24* (0.07,0.42)	0.19 (0.00,0.39)
Conditional relative weight in adolescence	0.30* (0.23,0.37)	0.50* (0.40,0.59)	0.30* (0.23,0.37)	0.45* (0.36,0.53)	-0.01 (-0.54,0.53)	0.32 (-0.11,0.75)	0.21* (0.11,0.3)	0.41* (0.25,0.57)	0.34* (0.25,0.43)	0.40* (0.30,0.51)	0.49* (0.39,0.59)	0.46* (0.36,0.56)
Conditional height in infancy	0.00 (-0.07,0.07)	0.16* (0.06,0.25)	0.05 (0,0.09)	0.04 (-0.03,0.1)	0.16 (-0.18,0.51)	-0.02 (-0.33,0.29)	0.06 (-0.04,0.16)	0.17* (0.01,0.32)	0.11* (0.04,0.19)	0.13* (0.05,0.21)	0.06 (-0.04,0.16)	0.04 (-0.06,0.15)
Conditional height in childhood	-0.03 (-0.15,0.08)	0.03 (-0.13,0.20)	0.12* (0.05,0.19)	0.13* (0.04,0.23)	-0.09 (-0.57,0.4)	0.24 (-0.42,0.91)	0.10 (-0.09,0.28)	0.18 (-0.06,0.43)	0.13* (0.02,0.25)	0.24* (0.12,0.36)	0.08 (-0.10,0.26)	0.17 (-0.02,0.35)
Conditional height in adolescence	0.05 (-0.05,0.14)	0.26* (0.12,0.41)	0.08* (0.01,0.16)	-0.01 (-0.14,0.12)	0.42 (-0.08,0.92)	-0.03 (-0.66,0.60)	-0.01 (-0.14,0.11)	0.07 (-0.22,0.35)	0.02 (-0.13,0.17)	-0.11 (-0.29,0.07)	-0.12 (-0.28,0.04)	-0.18* (-0.35,-0.01)

[1] Values are standardized linear regression coefficients (βs and 95% CIs). In adjusted analyses we controlled for birth characteristics (gestational age, birth order), maternal characteristics (height, age at childbirth, schooling, marital status), paternal characteristics (age, schooling), income and/or wealth index of child's household at birth, and age in adulthood. In women' models, we adjusted for age of menarche and teenage childbearing. Additionally, we controlled for maternal skin color in both Brazilian cohorts and adjusted for birth year and intervention group (village fixed effects) in Guatemala analysis. * P value < 0.05; SD = Standard Deviation

[2] We used birth weight as the anchor in all conditional models given that this measure was available across all study sites

BMJ Publishing Group Limited (BMJ) disclaims all liability and responsibility arising from any reliance
placed on this supplemental material which has been supplied by the author(s)

Supplementary Table 7. Adjusted associations between weight at birth, conditional growth (height and relative weight) in infancy, childhood, and adolescence with adult body mass index (BMI, SD units), stratified by study site and sex[1]

	**Brazil 1982**		**Brazil 1993**		**Guatemala**		**India**		**The Philippines**		**South Africa**	
	Men<br>(n = 344)	Women<br>(n = 330)	Men<br>(n = 375)	Women<br>(n = 452)	Men<br>(n = 67)	Women<br>(n = 96)	Men<br>(n = 426)	Women<br>(n = 255)	Men<br>(n = 656)	Women<br>(n = 541)	Men<br>(n = 287)	Women<br>(n = 308)
**Conditional growth, z-scores[2]**												
Birth weight	0.08*<br>(0.02,0.15)	0.02<br>(-0.07,0.10)	0.04<br>(-0.02,0.10)	0.13*<br>(0.07,0.20)	0.03<br>(-0.24,0.29)	0.33**<br>(0.12,0.55)	0.14*<br>(0.04,0.23)	0.17*<br>(0.03,0.30)	0.12*<br>(0.04,0.19)	0.12*<br>(0.04,0.20)	0.11*<br>(0.02,0.20)	0.07<br>(-0.02,0.15)
Conditional relative weight in infancy	0.29*<br>(0.19,0.39)	0.36*<br>(0.21,0.5)	0.33*<br>(0.23,0.42)	0.33*<br>(0.24,0.42)	0.51<br>(-0.02,1.04)	0.07<br>(-0.36,0.5)	0.28*<br>(0.15,0.41)	0.32*<br>(0.14,0.50)	0.28*<br>(0.18,0.38)	0.31*<br>(0.19,0.43)	0.22*<br>(0.14,0.31)	0.24*<br>(0.13,0.35)
Conditional relative weight in childhood	0.53*<br>(0.38,0.67)	0.59*<br>(0.4,0.79)	0.47*<br>(0.38,0.57)	0.62*<br>(0.52,0.73)	0.19<br>(-0.61,0.99)	0.38<br>(-0.19,0.95)	0.44*<br>(0.24,0.64)	0.17<br>(-0.08,0.42)	0.65*<br>(0.53,0.78)	0.70*<br>(0.54,0.85)	0.35*<br>(0.21,0.49)	0.37*<br>(0.2,0.53)
Conditional relative weight in adolescence	0.54*<br>(0.46,0.62)	0.70*<br>(0.6,0.79)	0.49*<br>(0.42,0.55)	0.66*<br>(0.55,0.77)	0.42.<br>(-0.07,0.91)	0.57*<br>(0.20,0.94)	0.35*<br>(0.27,0.44)	0.54*<br>(0.39,0.69)	0.43*<br>(0.34,0.52)	0.44*<br>(0.34,0.54)	0.67*<br>(0.59,0.75)	0.63*<br>(0.55,0.72)
Conditional height in infancy	0.04<br>(-0.04,0.11)	0.16*<br>(0.06,0.26)	0.04<br>(-0.01,0.10)	0.08*<br>(0.01,0.15)	0.35*<br>(0.03,0.66)	0.14<br>(-0.13,0.41)	0.12*<br>(0.03,0.21)	0.07<br>(-0.08,0.22)	0.13*<br>(0.06,0.20)	0.14*<br>(0.06,0.21)	0.06<br>(-0.02,0.14)	0.13*<br>(0.04,0.22)
Conditional height in childhood	-0.10<br>(-0.23,0.03)	-0.02<br>(-0.19,0.15)	0.13*<br>(0.05,0.21)	0.14*<br>(0.04,0.25)	-0.13<br>(-0.57,0.31)	-0.19<br>(-0.76,0.39)	0.11<br>(-0.06,0.27)	0.04<br>(-0.2,0.27)	0.15*<br>(0.04,0.26)	0.27*<br>(0.15,0.39)	0.03<br>(-0.11,0.18)	0.14<br>(-0.02,0.29)
Conditional height in adolescence	0.06<br>(-0.04,0.16)	0.18*<br>(0.03,0.33)	0.03<br>(-0.06,0.12)	0.07<br>(-0.06,0.19)	0.34<br>(-0.11,0.79)	-0.06<br>(-0.61,0.48)	0.03<br>(-0.08,0.14)	-0.01<br>(-0.28,0.26)	-0.03<br>(-0.17,0.11)	-0.09<br>(-0.27,0.08)	-0.09<br>(-0.21,0.04)	-0.03<br>(-0.17,0.11)

[1] Values are standardized linear regression coefficients (βs and 95% CIs). In adjusted analyses we controlled for birth characteristics (gestational age, birth order), maternal characteristics (height, age at childbirth, schooling, marital status), paternal characteristics (age, schooling), income and/or wealth index of child's household at birth, and age in adulthood. In women' models, we adjusted for age of menarche and teenage childbearing. Additionally, we controlled for maternal skin color in both Brazilian cohorts and adjusted for birth year and intervention group (village fixed effects) in Guatemala analysis. * P value < 0.05; SD = Standard Deviation

[2] We used birth weight as the anchor in all conditional models given that this measure was available across all study sites

BMJ Publishing Group Limited (BMJ) disclaims all liability and responsibility arising from any reliance placed on this supplemental material which has been supplied by the author(s)

Supplementary Table 8. Adjusted associations between weight at birth, conditional growth (height and relative weight) in infancy, childhood, and adolescence with adult waist circumference (SD units), stratified by study site and sex[1]

	Brazil 1982		Brazil 1993		Guatemala		India		The Philippines		South Africa	
	Men (n = 344)	Women (n = 330)	Men (n = 375)	Women (n = 452)	Men (n = 67)	Women (n = 96)	Men (n = 426)	Women (n = 255)	Men (n = 656)	Women (n = 541)	Men (n = 287)	Women (n = 308)
**Conditional growth, z-scores[2]**												
Birth weight	0.06 (-0.03,0.14)	0.03 (-0.07,0.13)	0.03 (-0.04,0.10)	0.11* (0.01,0.21)	0.03 (-0.26,0.32)	0.32* (0.05,0.58)	0.14* (0.03,0.24)	0.13 (-0.03,0.30)	0.09* (0.01,0.18)	0.08 (-0.02,0.18)	0.03 (-0.08,0.14)	0.01 (-0.10,0.12)
Conditional relative weight in infancy	0.26* (0.14,0.37)	0.27* (0.14,0.39)	0.33* (0.22,0.43)	0.30* (0.21,0.39)	0.51 (-0.05,1.08)	0.12 (-0.33,0.56)	0.23* (0.10,0.37)	0.31* (0.13,0.49)	0.25* (0.14,0.35)	0.25* (0.12,0.37)	0.17* (0.07,0.27)	0.17* (0.04,0.3)
Conditional relative weight in childhood	0.49* (0.32,0.66)	0.50* (0.32,0.67)	0.45* (0.33,0.57)	0.50* (0.38,0.62)	0.29 (-0.52,1.11)	0.17 (-0.4,0.73)	0.44* (0.24,0.65)	0.02 (-0.24,0.28)	0.47* (0.33,0.6)	0.57* (0.41,0.73)	0.28* (0.11,0.45)	0.23* (0.03,0.42)
Conditional relative weight in adolescence	0.53* (0.44,0.62)	0.52* (0.44,0.61)	0.45* (0.37,0.53)	0.58* (0.48,0.68)	0.38 (-0.16,0.91)	0.50* (0.13,0.87)	0.26* (0.16,0.35)	0.44* (0.28,0.6)	0.33* (0.23,0.42)	0.41* (0.30,0.51)	0.51* (0.41,0.61)	0.51* (0.41,0.60)
Conditional height in infancy	0.04 (-0.06,0.15)	0.22* (0.07,0.36)	0.08* (0.00,0.16)	0.08 (-0.05,0.21)	0.28 (-0.08,0.64)	-0.08 (-0.58,0.42)	0.11 (0.00,0.22)	-0.16 (-0.40,0.09)	0.22* (0.12,0.31)	0.11 (-0.06,0.27)	0.02 (-0.1,0.14)	0.10 (-0.06,0.26)
Conditional height in childhood	-0.06 (-0.22,0.1)	0.10 (-0.11,0.31)	0.19* (0.08,0.3)	0.19 (-0.01,0.38)	-0.19 (-0.66,0.28)	-0.22 (-0.85,0.42)	0.12 (-0.06,0.3)	0.12 (-0.22,0.46)	0.23* (0.09,0.38)	0.20 (-0.04,0.43)	0.00 (-0.21,0.21)	0.15 (-0.10,0.4)
Conditional height in adolescence	0.06 (-0.06,0.19)	0.26* (0.03,0.49)	0.10 (0.00,0.21)	0.07 (-0.16,0.31)	0.28 (-0.34,0.89)	-0.38 (-1.38,0.63)	0.06 (-0.06,0.19)	-0.17 (-0.61,0.27)	0.13 (-0.05,0.31)	-0.13 (-0.49,0.22)	-0.07 (-0.26,0.11)	-0.15 (-0.43,0.13)

[1] Values are standardized linear regression coefficients (βs and 95% CIs). In adjusted analyses we controlled for birth characteristics (gestational age, birth order), maternal characteristics (height, age at childbirth, schooling, marital status), paternal characteristics (age, schooling), income and/or wealth index of child's household at birth, and age and height in adulthood. In women' models, we adjusted for age of menarche and teenage childbearing. Additionally, we controlled for maternal skin color in both Brazilian cohorts and adjusted for birth year and intervention group (village fixed effects) in Guatemala analysis. * P value < 0.05; SD = Standard Deviation

[2] We used birth weight as the anchor in all conditional models given that this measure was available across all study sites

Supplementary Table 9. Pooled adjusted associations between length at birth, conditional growth (height and relative weight) in infancy, childhood, and adolescence with adult fat mass and fat-free mass indices, stratified by sex[1]

	FMI (SD units)						FFMI (SD units)					
	Men (n = 1524)			Women (n = 1344)			Men (n = 1524)			Women (n = 1344)		
	β (95%CI)	I^2 statistic (%)[3]	Cochran's Q test (P value)	β (95%CI)	I^2 statistic (%)	Cochran's Q test (P value)	β (95%CI)	I^2 statistic (%)	Cochran's Q test (P value)	β (95%CI)	I^2 statistic (%)	Cochran's Q test (P value)
**Conditional growth, z-scores[2]**												
Birth length	0.06 (-0.03,0.15)	50.2	0.128	0.09 (-0.01,0.20)	46.4	0.165	0.06 (-0.02,0.15)	54.8	0.083	0.07 (0.00,0.14)	7.4	0.325
Conditional height in infancy	0.11* (0.04,0.19)	2.5	0.378	0.11* (0.04,0.18)	16.7	0.539	0.12 (-0.06,0.3)	76.4	0.004	0.10 (-0.07,0.27)	78.8	0.006
Conditional height in childhood	0.11* (0.03,0.20)	0.0	0.714	0.16* (0.03,0.30)	20.2	0.425	0.11 (-0.04,0.25)	41.1	0.175	0.06 (-0.32,0.45)	88.2	0.001
Conditional height in adolescence	0.03 (-0.09,0.16)	0.0	0.402	-0.03 (-0.14,0.07)	0.0	0.768	0.00 (-0.17,0.17)	47.7	0.113	-0.04 (-0.22,0.14)	44.1	0.194
Conditional relative weight at birth	0.09* (0.04,0.14)	0.0	0.870	0.11* (0.03,0.20)	0.0	0.556	0.10* (0.02,0.18)	0.0	0.527	0.14 (-0.05,0.33)	52.8	0.070
Conditional relative weight in infancy	0.23* (0.17,0.30)	0.0	0.819	0.25* (0.11,0.39)	8.9	0.297	0.32* (0.27,0.37)	0.0	0.871	0.34* (0.24,0.44)	8.1	0.511
Conditional relative weight in childhood	0.43* (0.17,0.70)	71.1	0.009	0.49 (-0.03,1.03)	90	<0.001	0.55* (0.3,0.81)	75.7	0.003	0.50* (0.19,0.82)	76.7	0.005
Conditional relative weight in adolescence	0.34* (0.24,0.44)	29.3	0.282	0.49* (0.37,0.60)	34.5	0.296	0.41* (0.29,0.54)	56	0.090	0.45* (0.34,0.56)	43.2	0.206

[1] Values are standardized linear regression coefficients (*β*s and 95% CIs). FMI, fat mass index; FFMI, fat-free mass index. In adjusted analyses we controlled for birth characteristics (gestational age, birth order), maternal characteristics (height, age at childbirth, schooling, marital status), paternal schooling, income and/or wealth index of child's household at birth, and age in adulthood. In women' models, we adjusted for age of menarche and teenage childbearing. Additionally, we controlled for maternal skin color in Brazil and adjusted for birth year and intervention group (village fixed effects) in Guatemala analysis. * P value < 0.05

[2] We used birth length as the anchor in conditional models of sites that collected this measure (Brazil 1993, Guatemala, India, The Philippines)

[3] As a rule of thumb $I^2 = 25\%$ is interpreted as low heterogeneity, 50% moderate heterogeneity, and 75% substantial heterogeneity

Supplementary Table 10. Pooled adjusted associations between length at birth, conditional growth (height and relative weight) in infancy, childhood, and adolescence with adult body mass index and waist circumference, stratified by sex[1]

	BMI (SD units)						Waist circumference (SD units)					
	Men (n = 1524)			Women (n = 1344)			Men (n = 1524)			Women (n = 1344)		
	β (95%CI)	I^2 statistic (%)[3]	Cochran's Q test (P value)	β (95%CI)	I^2 statistic (%)	Cochran's Q test (P value)	β (95%CI)	I^2 statistic (%)	Cochran's Q test (P value)	β (95%CI)	I^2 statistic (%)	Cochran's Q test (P value)
**Conditional growth, z-scores[2]**												
Birth length	0.06 (-0.03,0.16)	57.5	0.070	0.10* (0.03,0.17)	0.9	0.389	0.06 (-0.04,0.17)	50.1	0.116	0.06 (-0.01,0.12)	0.0	0.739
Conditional height in infancy	0.12* (0.02,0.23)	29.9	0.149	0.11* (0.04,0.18)	1.7	0.540	0.16* (0.05,0.28)	29.4	0.258	0.05 (-0.15,0.24)	29.1	0.255
Conditional height in childhood	0.12* (0.04,0.21)	0.2	0.651	0.15 (-0.07,0.37)	53.8	0.101	0.18* (0.02,0.33)	0.1	0.309	0.15* (0.00,0.3)	0.0	0.725
Conditional height in adolescence	0.02 (-0.09,0.13)	0.1	0.503	-0.03 (-0.16,0.11)	11.1	0.573	0.11* (0.04,0.18)	0.0	0.871	-0.09 (-0.33,0.15)	0.0	0.631
Conditional relative weight at birth	0.10* (0.07,0.13)	0.0	0.955	0.13 (0.00,0.26)	0.0	0.236	0.08* (0.02,0.14)	0.0	0.808	0.13 (-0.01,0.28)	0.1	0.166
Conditional relative weight in infancy	0.30* (0.24,0.35)	0.0	0.840	0.30* (0.22,0.38)	0.0	0.700	0.27* (0.19,0.35)	0.0	0.642	0.26* (0.19,0.34)	0.0	0.752
Conditional relative weight in childhood	0.53* (0.33,0.73)	54.6	0.095	0.55* (0.11,0.98)	86	0.001	0.45* (0.41,0.49)	0.0	0.982	0.41 (-0.04,0.86)	85.5	0.002
Conditional relative weight in adolescence	0.42* (0.31,0.53)	55.4	0.109	0.54* (0.37,0.71)	63.8	0.030	0.34* (0.19,0.49)	68.8	0.023	0.48* (0.33,0.62)	51.5	0.114

[1] Values are standardized linear regression coefficients (*β*s and 95% CIs). BMI, body mass index. In adjusted analyses we controlled for birth characteristics (gestational age, birth order), maternal characteristics (height, age at childbirth, schooling, marital status), paternal schooling, income and/or wealth index of child's household at birth, and age in adulthood. In women' models, we adjusted for age of menarche and teenage childbearing. Additionally, we controlled for maternal skin color in Brazil and adjusted for birth year and intervention group (village fixed effects) in Guatemala analysis. * P value < 0.05
[2] We used birth length as the anchor in conditional models of sites that collected this measure (Brazil 1993, Guatemala, India, The Philippines)
[3] As a rule of thumb I^2 = 25% is interpreted as low heterogeneity, 50% moderate heterogeneity, and 75% substantial heterogeneity

Supplementary Table 11. Adjusted associations between length at birth, conditional growth (height and relative weight) in infancy, childhood, and adolescence with adult fat mass index (FMI, SD units), stratified by study site and sex[1]

	Brazil 1982		Brazil 1993		Guatemala		India		The Philippines		South Africa	
	Men (n = 344)	Women (n = 330)	Men (n = 375)	Women (n = 452)	Men (n = 67)	Women (n = 96)	Men (n = 426)	Women (n = 255)	Men (n = 656)	Women (n = 541)	Men (n = 287)	Women (n = 308)
**Conditional growth, z-scores[2]**												
Birth length	NA	NA	-0.01 (-0.06,0.05)	0.07* (0.01,0.14)	0.00 (-0.23,0.23)	0.06 (-0.15,0.28)	0.11* (0.02,0.20)	0.21* (0.10,0.32)	0.07* (0.01,0.14)	0.07 (0.00,0.14)	NA	NA
Conditional height in infancy	NA	NA	0.07* (0.02,0.13)	0.07* (0,0.14)	0.31 (-0.04,0.66)	0.11 (-0.2,0.42)	0.09 (-0.01,0.2)	0.09 (-0.06,0.25)	0.13* (0.06,0.20)	0.15* (0.07,0.22)	NA	NA
Conditional height in childhood	NA	NA	0.14* (0.06,0.22)	0.14* (0.03,0.24)	-0.12 (-0.59,0.35)	0.02 (-0.62,0.65)	0.10 (-0.08,0.27)	0.09 (-0.15,0.33)	0.14* (0.03,0.25)	0.25* (0.13,0.37)	NA	NA
Conditional height in adolescence	NA	NA	0.07 (-0.01,0.16)	0.03 (-0.1,0.15)	0.35 (-0.14,0.84)	-0.05 (-0.65,0.55)	0.00 (-0.11,0.12)	-0.02 (-0.30,0.26)	0.00 (-0.15,0.14)	-0.09 (-0.27,0.09)	NA	NA
Conditional relative weight at birth	NA	NA	0.07 (-0.03,0.17)	0.12* (0.02,0.22)	0.14 (-0.18,0.47)	0.23 (-0.05,0.51)	0.13 (0.00,0.27)	0.00 (-0.19,0.19)	0.08 (-0.02,0.17)	0.12* (0.02,0.22)	NA	NA
Conditional relative weight in infancy	NA	NA	0.21* (0.11,0.31)	0.22* (0.13,0.31)	0.42 (-0.16,1.01)	-0.06 (-0.53,0.41)	0.20* (0.06,0.34)	0.26* (0.07,0.44)	0.25* (0.15,0.36)	0.33* (0.21,0.45)	NA	NA
Conditional relative weight in childhood	NA	NA	0.32* (0.22,0.42)	0.50* (0.40,0.61)	0.04 (-0.82,0.89)	0.45 (-0.18,1.07)	0.36* (0.15,0.57)	0.01 (-0.25,0.26)	0.59* (0.46,0.72)	0.72* (0.56,0.88)	NA	NA
Conditional relative weight in adolescence	NA	NA	0.38* (0.30,0.45)	0.56* (0.45,0.66)	0.16 (-0.36,0.69)	0.53* (0.13,0.94)	0.28* (0.19,0.37)	0.50* (0.35,0.65)	0.38* (0.29,0.47)	0.42* (0.32,0.52)	NA	NA

[1] Values are standardized linear regression coefficients (βs and 95% CIs). In adjusted analyses we controlled for birth characteristics (gestational age, birth order), maternal characteristics (height, age at childbirth, schooling, marital status), paternal characteristics (age, schooling), income and/or wealth index of child's household at birth, and age in adulthood. In women' models, we adjusted for age of menarche and teenage childbearing. Additionally, we controlled for maternal skin color in Brazil and adjusted for birth year and intervention group (village fixed effects) in Guatemala analysis. * P value < 0.05; SD = Standard Deviation

[2] We used birth length as the anchor in conditional models of sites that collected this measure (Brazil 1993, Guatemala, India, The Philippines)

Supplementary Table 12. Adjusted associations between length at birth, conditional growth (height and relative weight) in infancy, childhood, and adolescence with adult fat-free mass index (FFMI, SD units), stratified by study site and sex[1]

	Brazil 1982		Brazil 1993		Guatemala		India		The Philippines		South Africa	
	Men (n = 344)	Women (n = 330)	Men (n = 375)	Women (n = 452)	Men (n = 67)	Women (n = 96)	Men (n = 426)	Women (n = 255)	Men (n = 656)	Women (n = 541)	Men (n = 287)	Women (n = 308)
**Conditional growth, z-scores[2]**												
Birth length	NA	NA	0.00 (-0.05,0.05)	0.08* (0.04,0.12)	0.00 (-0.20,0.20)	0.19 (-0.01,0.38)	0.09* (0.01,0.18)	0.09 (-0.02,0.19)	0.09* (0.03,0.15)	0.03 (-0.04,0.09)	NA	NA
Conditional height in infancy	NA	NA	0.01 (-0.05,0.07)	0.06* (0.02,0.11)	0.35* (0.05,0.66)	0.16 (-0.11,0.43)	0.13* (0.04,0.23)	-0.05 (-0.20,0.09)	0.14* (0.08,0.21)	0.19* (0.12,0.26)	NA	NA
Conditional height in childhood	NA	NA	0.03 (-0.06,0.12)	0.06 (0.00,0.12)	-0.10 (-0.52,0.31)	-0.40 (-0.95,0.16)	0.11 (-0.05,0.28)	-0.15 (-0.37,0.07)	0.17* (0.06,0.27)	0.24* (0.13,0.35)	NA	NA
Conditional height in adolescence	NA	NA	-0.08 (-0.18,0.02)	0.08 (-0.01,0.17)	0.25 (-0.19,0.68)	-0.05 (-0.58,0.48)	0.08 (-0.03,0.19)	-0.14 (-0.40,0.13)	-0.03 (-0.17,0.1)	-0.09 (-0.26,0.08)	NA	NA
Conditional relative weight at birth	NA	NA	0.13* (0.05,0.21)	0.09* (0.02,0.16)	-0.08 (-0.37,0.21)	0.42* (0.18,0.67)	0.08 (-0.05,0.21)	0.10 (-0.08,0.28)	0.11* (0.02,0.20)	0.14* (0.04,0.24)	NA	NA
Conditional relative weight in infancy	NA	NA	0.29* (0.21,0.37)	0.29* (0.23,0.36)	0.46 (-0.06,0.98)	0.22 (-0.19,0.63)	0.34* (0.21,0.46)	0.40* (0.23,0.58)	0.31* (0.21,0.41)	0.37* (0.25,0.49)	NA	NA
Conditional relative weight in childhood	NA	NA	0.43* (0.33,0.52)	0.41* (0.33,0.5)	0.33 (-0.43,1.10)	0.21 (-0.34,0.75)	0.46* (0.26,0.65)	0.36* (0.12,0.60)	0.71* (0.59,0.83)	0.70* (0.55,0.85)	NA	NA
Conditional relative weight in adolescence	NA	NA	0.34* (0.28,0.41)	0.38* (0.32,0.44)	0.59* (0.12,1.06)	0.45* (0.10,0.81)	0.36* (0.28,0.44)	0.52* (0.38,0.67)	0.47* (0.39,0.55)	0.47* (0.38,0.57)	NA	NA

[1] Values are standardized linear regression coefficients (βs and 95% CIs). In adjusted analyses we controlled for birth characteristics (gestational age, birth order), maternal characteristics (height, age at childbirth, schooling, marital status), paternal characteristics (age, schooling), income and/or wealth index of child's household at birth, and age in adulthood. In women' models, we adjusted for age of menarche and teenage childbearing. Additionally, we controlled for maternal skin color in Brazil and adjusted for birth year and intervention group (village fixed effects) in Guatemala analysis. * P value < 0.05; SD = Standard Deviation

[2] We used birth length as the anchor in conditional models of sites that collected this measure (Brazil 1993, Guatemala, India, The Philippines)

Supplementary Table 13. Adjusted associations length at birth, conditional growth (height and relative weight) in infancy, childhood, and adolescence with adult body mass index (BMI, SD units), stratified by study site and sex[1]

	Brazil 1982		Brazil 1993		Guatemala		India		The Philippines		South Africa	
	Men (n = 344)	Women (n = 330)	Men (n = 375)	Women (n = 452)	Men (n = 67)	Women (n = 96)	Men (n = 426)	Women (n = 255)	Men (n = 656)	Women (n = 541)	Men (n = 287)	Women (n = 308)
**Conditional growth, z-scores[2]**												
Birth length	NA	NA	-0.01 (-0.06,0.05)	0.10* (0.04,0.16)	0.00 (-0.22,0.22)	0.13 (-0.07,0.33)	0.10* (0.02,0.19)	0.18* (0.07,0.29)	0.08* (0.02,0.15)	0.07 (0.00,0.14)	NA	NA
Conditional height in infancy	NA	NA	0.06* (0.01,0.12)	0.09* (0.02,0.16)	0.37* (0.04,0.70)	0.15 (-0.13,0.44)	0.12* (0.02,0.21)	0.04 (-0.11,0.20)	0.14* (0.07,0.20)	0.15* (0.07,0.23)	NA	NA
Conditional height in childhood	NA	NA	0.13* (0.05,0.21)	0.14* (0.04,0.25)	-0.13 (-0.57,0.32)	-0.18 (-0.76,0.41)	0.11 (-0.06,0.27)	0.01 (-0.22,0.25)	0.15* (0.05,0.26)	0.27* (0.15,0.39)	NA	NA
Conditional height in adolescence	NA	NA	0.03 (-0.06,0.11)	0.06 (-0.06,0.19)	0.33 (-0.13,0.8)	-0.06 (-0.61,0.5)	0.03 (-0.08,0.15)	-0.05 (-0.32,0.22)	-0.03 (-0.17,0.11)	-0.08 (-0.26,0.09)	NA	NA
Conditional relative weight at birth	NA	NA	0.12* (0.02,0.21)	0.15* (0.04,0.25)	0.04 (-0.27,0.34)	0.36* (0.1,0.61)	0.11 (-0.02,0.24)	0.04 (-0.15,0.22)	0.09* (0.00,0.19)	0.12* (0.02,0.22)	NA	NA
Conditional relative weight in infancy	NA	NA	0.32* (0.22,0.41)	0.33* (0.24,0.42)	0.49 (-0.07,1.04)	0.06 (-0.37,0.50)	0.28* (0.15,0.41)	0.33* (0.15,0.51)	0.28* (0.18,0.38)	0.31* (0.19,0.43)	NA	NA
Conditional relative weight in childhood	NA	NA	0.47* (0.38,0.57)	0.63* (0.52,0.73)	0.20 (-0.61,1.01)	0.40 (-0.18,0.97)	0.44* (0.24,0.64)	0.14 (-0.11,0.39)	0.65* (0.53,0.78)	0.70* (0.54,0.85)	NA	NA
Conditional relative weight in adolescence	NA	NA	0.49* (0.42,0.55)	0.66* (0.55,0.77)	0.41 (-0.09,0.91)	0.57* (0.19,0.95)	0.35* (0.27,0.44)	0.54* (0.39,0.69)	0.43* (0.34,0.52)	0.44* (0.34,0.54)	NA	NA

[1] Values are standardized linear regression coefficients (βs and 95% CIs). In adjusted analyses we controlled for birth characteristics (gestational age, birth order), maternal characteristics (height, age at childbirth, schooling, marital status), paternal characteristics (age, schooling), income and/or wealth index of child's household at birth, and age in adulthood. In women' models, we adjusted for age of menarche and teenage childbearing. Additionally, we controlled for maternal skin color in Brazil and adjusted for birth year and intervention group (village fixed effects) in Guatemala analysis. * P value < 0.05; SD = Standard Deviation

[2] We used birth length as the anchor in conditional models of sites that collected this measure (Brazil 1993, Guatemala, India, The Philippines)

Supplementary Table 14. Adjusted associations between length at birth, conditional growth (height and relative weight) in infancy, childhood, and adolescence with adult waist circumference (SD units), stratified by study site and sex

	Brazil 1982		Brazil 1993		Guatemala		India		The Philippines		South Africa	
	Men (n = 344)	Women (n = 330)	Men (n = 375)	Women (n = 452)	Men (n = 67)	Women (n = 96)	Men (n = 426)	Women (n = 255)	Men (n = 656)	Women (n = 541)	Men (n = 287)	Women (n = 308)
**Conditional growth, z-scores[2]**												
Birth length	NA	NA	-0.01 (-0.07,0.05)	0.08 (-0.03,0.19)	-0.03 (-0.26,0.21)	0.03 (-0.22,0.28)	0.10* (0.00,0.20)	0.11 (-0.05,0.26)	0.09* (0.02,0.17)	0.02 (-0.09,0.12)	NA	NA
Conditional height in infancy	NA	NA	0.11* (0.03,0.19)	0.09 (-0.04,0.22)	0.32 (-0.06,0.69)	-0.03 (-0.53,0.47)	0.11 (0.00,0.23)	-0.16 (-0.40,0.08)	0.22* (0.12,0.31)	0.12 (-0.04,0.28)	NA	NA
Conditional height in childhood	NA	NA	0.19* (0.08,0.3)	0.18 (-0.01,0.38)	-0.19 (-0.66,0.28)	-0.18 (-0.82,0.46)	0.12 (-0.06,0.31)	0.12 (-0.22,0.46)	0.24* (0.09,0.38)	0.20 (-0.04,0.43)	NA	NA
Conditional height in adolescence	NA	NA	0.10 (-0.01,0.2)	0.06 (-0.17,0.29)	0.27 (-0.35,0.88)	-0.34 (-1.36,0.67)	0.07 (-0.06,0.19)	-0.18 (-0.62,0.26)	0.13 (-0.05,0.31)	-0.12 (-0.48,0.23)	NA	NA
Conditional relative weight at birth	NA	NA	0.10 (-0.01,0.21)	0.12* (0.02,0.23)	0.07 (-0.25,0.39)	0.42* (0.15,0.68)	0.11 (-0.02,0.25)	0.10 (-0.09,0.29)	0.04 (-0.05,0.14)	0.10 (0.00,0.21)	NA	NA
Conditional relative weight in infancy	NA	NA	0.32* (0.21,0.42)	0.30* (0.21,0.39)	0.47 (-0.12,1.06)	0.10 (-0.34,0.55)	0.23* (0.10,0.37)	0.31* (0.13,0.50)	0.25* (0.14,0.35)	0.24* (0.12,0.37)	NA	NA
Conditional relative weight in childhood	NA	NA	0.45* (0.33,0.57)	0.50* (0.39,0.62)	0.31 (-0.51,1.13)	0.19 (-0.38,0.76)	0.44* (0.24,0.65)	0.00 (-0.26,0.26)	0.47* (0.33,0.60)	0.56* (0.41,0.72)	NA	NA
Conditional relative weight in adolescence	NA	NA	0.45* (0.37,0.52)	0.58* (0.48,0.68)	0.35 (-0.20,0.90)	0.50* (0.13,0.87)	0.26* (0.16,0.35)	0.43* (0.27,0.60)	0.33* (0.23,0.42)	0.40* (0.30,0.51)	NA	NA

[1] Values are standardized linear regression coefficients (βs and 95% CIs). In adjusted analyses we controlled for birth characteristics (gestational age, birth order), maternal characteristics (height, age at childbirth, schooling, marital status), paternal characteristics (age, schooling), income and/or wealth index of child's household at birth, and age and height in adulthood. In women' models, we adjusted for age of menarche and teenage childbearing. Additionally, we controlled for maternal skin color in Brazil and adjusted for birth year and intervention group (village fixed effects) in Guatemala analysis. * P value < 0.05; SD = Standard Deviation

[2] We used birth length as the anchor in conditional models of sites that collected this measure (Brazil 1993, Guatemala, India, The Philip

Supplementary Table 15. Correlations between adult height and body composition measurements, stratified by study site and sex

	Brazil 1982		Brazil 1993		Guatemala		India		The Philippines		South Africa	
	r	p-value	r	p-value	r	p-value	r	p-value	r	p-value	r	p-value
**Men**												
BMI	0.00	0.944	0.02	0.744	0.01	0.935	-0.01	0.904	0.06	0.103	0.01	0.830
FMI	-0.01	0.888	0.04	0.450	0.10	0.445	0.02	0.704	0.05	0.160	0.04	0.504
FFMI	0.00	0.944	-0.02	0.682	-0.08	0.509	-0.01	0.851	0.07	0.059	0.01	0.872
WC	0.17	0.001	0.17	0.001	0.17	0.163	0.18	<0.001	0.21	<0.001	0.26	<0.001
**Women**												
BMI	-0.04	0.479	-0.09	0.065	0.03	0.806	0.01	0.888	0.05	0.246	-0.06	0.258
FMI	-0.02	0.749	-0.09	0.052	0.04	0.676	0.09	0.167	0.04	0.322	-0.07	0.219
FFMI	-0.08	0.131	-0.05	0.294	-0.01	0.945	-0.15	0.014	0.06	0.157	-0.01	0.794
WC	0.08	0.133	0.06	0.194	0.22	0.03	0.23	<0.001	0.18	<0.001	0.15	0.008

BMI: body mass index; FMI: fat mass index; FFMI: fat-free mass index; WC: waist circumference
