# Supplementary material for: Growth patterns in childhood and adolescence and adult body composition: a pooled analysis of birth cohort studies from five low and middle-income countries (COHORTS collaboration)
Source: BMJ Open. 2023 Mar 14;13(3):e068427. doi: 10.1136/bmjopen-2022-068427 (PMC10030655; doi:10.1136/bmjopen-2022-068427)
Supplement: Author's manuscript [file bmjopen-2022-068427.draft_revisions.pdf]

BMJ Open

BMJ Open is committed to open peer review. As part of this commitment we make the peer review history of every article we publish publicly available.

When an article is published we post the peer reviewers' comments and the authors' responses online. We also post the versions of the paper that were used during peer review. These are the versions that the peer review comments apply to.

The versions of the paper that follow are the versions that were submitted during the peer review process. They are not the versions of record or the final published versions. They should not be cited or distributed as the published version of this manuscript.

BMJ Open is an open access journal and the full, final, typeset and author-corrected version of record of the manuscript is available on our site with no access controls, subscription charges or pay-per-view fees (http://bmjopen.bmj.com).

If you have any questions on BMJ Open's open peer review process please email info.bmjopen@bmj.com

BMJ Open

Associations of growth patterns in childhood and adolescence with adult body composition: findings from six birth cohorts in five low- and middle-income countries (COHORTS collaboration)

Journal:	*BMJ Open*
Manuscript ID	bmjopen-2022-068427
Article Type:	Original research
Date Submitted by the Author:	25-Sep-2022

Complete List of Authors:	Poveda Rey, Natalia Elvira; Emory University, Rollins School of Public Health<br>Adair, Linda; University of North Carolina at Chapel Hill, Carolina Population Center<br>Martorell, Reynaldo; Emory University School of Public Health, Hubert Department of Global Health<br>Patel, Shivani; Emory University, Global Health<br>Ramirez-Zea, Manuel; Institute of Nutrition of Central America and Panama (INCAP), INCAP Research Center for the Prevention of Chronic Diseases (CIIPEC)<br>Bhargava, Santosh; Senior Consultant Pediatrics and Founder of the New Delhi Birth Cohort<br>Bechayda, Sonny A; University of San Carlos - Talamban Campus, Office of Population Studies Foundation<br>Carba, Delia B; University of San Carlos - Talamban Campus, Office of Population Studies Foundation<br>Kroker-Lobos, Maria; Institute of Nutrition of Central America and Panama (INCAP), INCAP Research Center for the Prevention of Chronic Diseases (CIIPEC)<br>Horta, Bernardo; Federal University of Pelotas, Post-Graduate Program in Epidemiology<br>Lima, Natália; Federal University of Pelotas<br>Mazariegos, Mónica; Institute of Nutrition of Central America and Panama (INCAP), INCAP Research Center for the Prevention of Chronic Diseases (CIIPEC)<br>Menezes, Ana Maria ; Federal University of Pelotas, Post-Graduate Program in Epidemiology<br>Norris, Shane; University of the Witwatersrand, SAMRC/Wits Developmental Pathways for Health Research Unit, Department of Pediatrics, Faculty of Health Sciences<br>Nyati, Lukhanyo H; University of the Witwatersrand, SAMRC/Wits Developmental Pathways for Health Research Unit, Department of Pediatrics, Faculty of Health Sciences<br>Richter, Linda; University of the Witwatersrand, DSI-NRF Centre of Excellence in Human Development<br>Sachdev, Harshpal; Sitaram Bhartia Institute of Science and Research, Senior Consultant Pediatrics and Clinical Epidemiology<br>Wehrmeister , Fernando ; Universidade Federal de Pelotas, Post-

Graduate Program in Epidemiology
Stein, Aryeh D.; Emory University, Hubert Department of Global Health

Keywords: EPIDEMIOLOGY, PUBLIC HEALTH, NUTRITION & DIETETICS

SCHOLARONE™
Manuscripts

I, the Submitting Author has the right to grant and does grant on behalf of all authors of the Work (as defined in the below author licence), an exclusive licence and/or a non-exclusive licence for contributions from authors who are: i) UK Crown employees; ii) where BMJ has agreed a CC-BY licence shall apply, and/or iii) in accordance with the terms applicable for US Federal Government officers or employees acting as part of their official duties; on a worldwide, perpetual, irrevocable, royalty-free basis to BMJ Publishing Group Ltd ("BMJ") its licensees and where the relevant Journal is co-owned by BMJ to the co-owners of the Journal, to publish the Work in this journal and any other BMJ products and to exploit all rights, as set out in our licence.

The Submitting Author accepts and understands that any supply made under these terms is made by BMJ to the Submitting Author unless you are acting as an employee on behalf of your employer or a postgraduate student of an affiliated institution which is paying any applicable article publishing charge ("APC") for Open Access articles. Where the Submitting Author wishes to make the Work available on an Open Access basis (and intends to pay the relevant APC), the terms of reuse of such Open Access shall be governed by a Creative Commons licence – details of these licences and which Creative Commons licence will apply to this Work are set out in our licence referred to above.

Other than as permitted in any relevant BMJ Author's Self Archiving Policies, I confirm this Work has not been accepted for publication elsewhere, is not being considered for publication elsewhere and does not duplicate material already published. I confirm all authors consent to publication of this Work and authorise the granting of this licence.

For peer review only

Associations of growth patterns in childhood and adolescence with adult body composition: findings from six birth cohorts in five low- and middle-income countries (COHORTS collaboration)

Author names:

Natalia E Poveda,[1] Linda S Adair,[2] Reynaldo Martorell,[3] Shivani A Patel,[3] Manuel Ramirez-Zea,[4] Santosh K Bhargava,[5] Sonny A Bechayda,[6] Delia B Carba,[6] María F Kroker-Lobos,[4] Bernardo L Horta,[7] Natalia P Lima,[7] Mónica Mazariegos,[4] Ana M B Menezes,[7] Shane A Norris,[8] Lukhanyo H Nyati,[8] Linda M Richter,[9] Harshpal Singh Sachdev,[10] Fernando C Wehrmeister,[7] Aryeh D Stein,[3] and COHORTS Group[11]

Author affiliations

[1]Nutrition and Health Sciences, Laney Graduate School, Emory University, Atlanta, GA, USA

[2]Department of Nutrition, Gillings School of Global Public Health, University of North Carolina at Chapel Hill, Chapel Hill, NC, USA

[3]Hubert Department of Global Health, Rollins School of Public Health, Emory University, Atlanta GA, USA

[4]INCAP Research Center for the Prevention of Chronic Diseases (CIIPEC), Institute of Nutrition of Central America and Panama (INCAP), Guatemala City, Guatemala

[5]Senior Consultant Pediatrics and Founder of the New Delhi Birth Cohort, D-7 Gulmohar Park, New Delhi 110049, India

[6]Office of Population Studies Foundation, University of San Carlos, Talamban, Cebu City, The Philippines

[7]Post-Graduate Program in Epidemiology, Federal University of Pelotas, Pelotas, Brazil

[8]SAMRC/Wits Developmental Pathways for Health Research Unit, Department of Pediatrics, Faculty of Health Sciences, University of the Witwatersrand, Johannesburg, South Africa.

[9]DSI-NRF Centre of Excellence in Human Development, University of the Witwatersrand, Johannesburg, South Africa

For peer review only

[10]Senior Consultant Pediatrics and Clinical Epidemiology, Sitaram Bhartia Institute of Science and Research, B-16 Qutab Institutional Area, New Delhi, India

[11]Additional members of the COHORTS Group include: **Pelotas Birth Cohorts**: Cesar G Victora; **New Delhi Birth Cohort**: Caroline HD Fall, Clive Osmond, Lakshmy Ramakrishnan, Bhaskar Singh, and Sikha Sinha; **INCAP Nutrition Supplementation Trial Longitudinal Study**: Adela Sanchez; **Cebu Longitudinal Health and Nutrition Survey:** Isabelita Bas, Judith B Borja, Nanette R Lee, Lorna L Perez

Correspondence to:

Aryeh D Stein, PhD

Rollins School of Public Health of Emory University

1518 Clifton Rd NE #7007

Atlanta GA 30322, USA

Aryeh.stein@emory.edu

ORCID iDs

Natalia E Poveda https://orcid.org/0000-0001-6070-362X

Linda S Adair https//orcid.org/ 0000-0002-3670-8073

Reynaldo Martorell https://orcid.org/ 0000-0001-8862-7243

Shivani A Patel https://orcid.org/0000-0003-0082-5857

Manuel Ramirez-Zea https://orcid.org/0000-0001-5107-9175

Sonny A Bechayda https://orcid.org/0000-0003-0076-5276

Bernardo L Horta https://orcid.org/ 0000-0001-9843-412X

Mónica Mazariegos https://orcid.org/0000-0002-2250-3683

Ana M B Menezes https://orcid.org/ 0000-0002-2996-9427

For peer review only

52 Shane A Norris https://orcid.org/ 0000-0001-7124-3788

53 Linda M Richter https://orcid.org/ 0000-0002-3654-3192

54 Harshpal Singh Sachdev https://orcid.org/0000-0002-4956-9391

55 Fernando C Wehrmeister https://orcid.org/0000-0001-7137-1747

56 Aryeh D Stein http://orcid.org/0000-0003-1138-6458

For peer review only

ABSTRACT

Objective We examined the associations of linear growth and relative weight in four age intervals (prenatal, infancy, childhood, and adolescence) with adult body composition.

Design Secondary data analysis of prospective birth cohort studies

Settings Data from six prospective birth cohorts from five low- and middle-income countries (Brazil, Guatemala, India, Philippines and South Africa).

Participants A combined analytic sample of 4173 participants who were followed from birth to ages 22-46 years. Our analyses included participants with complete and valid anthropometric data (weight and height) at birth, infancy, childhood and adolescence, and body composition data in adult life.

Exposures Birth weight, and conditional growth measures (conditional height and conditional relative weight) in infancy, childhood, and adolescence

Primary outcome measures Body composition [body mass index (BMI), fat mass index (FMI), fat-free mass index (FFMI), fat mass/fat-free mass ratio (FM/FFM), and waist circumference] in young and mid-adulthood

Results In pooled analyses of the six cohorts, we found that birthweight was a positive predictor of FMI, FFMI, BMI and waist circumference in adulthood. Relative weight gain by one SD in infancy was associated with increments of 0.2-0.3 SD in all adult outcomes. Relative weight gains in childhood and adolescence were the strongest predictors of adult body composition outcomes (effect sizes ranged between 0.31 and 0.58 SD units for each SD of conditional weight). Conditional height in infancy was associated with increments of 0.1 SD units in adult adiposity (FMI and BMI among men and women, and waist circumference among women). Site- and sex-stratified analyses showed consistency in the direction of estimates, although there were differences in their magnitude.

Conclusions Prenatal and postnatal relative weight gains were significant predictors of body size and composition in young and mid-adulthood. Conditional height in infancy was a significant but weak predictor of adult adiposity and did not predict FFMI.

For peer review only

Keywords: fat mass, fat-free mass, body mass index, waist circumference, growth, conditional height, conditional relative weight, cohort studies

Strengths and limitations of this study

- The prospective design of these six well-characterized population-based birth cohorts from five low- and middle-income countries. However, attrition might be one source of selection bias given the long-lasting follow-up.
- We analyzed two compartments of body composition (FMI and FFMI), beyond BMI, that were measured using gold standards and/or validated techniques at each study site.
- The use of conditional growth measures allowed us to break the high correlation between weight and height and to study the independent role of linear growth and relative weight at specific age intervals. However, the definition of these age intervals differed between some study sites because it was guided by data availability.
- We selected the best model at each study site, fully adjusted by multiple confounders, and applied a random effects meta-analysis that considers the heterogeneity between study sites. However, residual confounding might be a potential bias since we could not control for covariates such as diet, reproductive characteristics, and life style factors.

For peer review only

Introduction

Low and middle-income countries (LMICs) have witnessed an increase in the prevalence of cardiometabolic risk factors such as diabetes, hypertension, and obesity in the adult population.[1-3] Overweight and obesity are not only metabolic outcomes but also serve as risk factors for other cardiometabolic diseases.[4] The developmental origins of health and disease (DOHaD) hypothesis proposes that anatomic, physiologic, and metabolic adaptations in early life have long-lasting effects on the onset of adult cardiometabolic disease.[5]

Adaptations in body composition might have an important role in the programming of cardiometabolic disease "*through its own programming by early growth, and/or through being a mediator of the programming process*".[6] A recent review concluded that associations between infant growth and long-term cardiometabolic outcomes might be mediated by body composition after estimates were attenuated when controlling for current BMI.[7] Similarly, other systematic reviews and one meta-analysis showed that childhood obesity is a risk factor of cardiometabolic outcomes in adulthood. However, results were inconclusive in demonstrating whether or not this excessive weight gain in childhood was an independent factor or whether most of this effect was mediated through contemporary adult BMI.[8-10]

BMI in infancy, childhood, and adolescence has a moderate to strong tracking into adult adiposity.[8 11] However, the discrimination between body compartments such as fat and lean mass, which have different metabolic functions, is more relevant to understand cardiometabolic risk than BMI alone.[12] The study of the role of growth across the lifespan on body composition has demonstrated similarities and differences when comparing body compartments. Overall studies have shown that birth weight is positively associated with lean mass later in life,[6] and that it is a stronger predictor of fat-free mass (FFM) than fat mass (FM).[13 14] Less consistency (positive inverse and null findings) has been observed in studies that have examined the association between birthweight (low and high) with adiposity in childhood,

adolescence, and adulthood.[6 15 16] Additionally, studies in high-income countries (HIC) have shown that a rapid weight gain (≥ 0.67 SD) in infancy (first 24 months after birth) are associated with higher obesity and fat mass in childhood and adulthood.[7 17] Whereas findings in LMICs have shown that weight gain in infancy is associated with higher FM and FFM in adult life, being a stronger predictor of FFM.[6 7 17]

There is a paucity of studies about the long-term effects of growth across the life span on adult body composition. Available evidence has primarily examined associations between prenatal and postnatal growth (mainly in infancy) with body composition in childhood and adolescence. In a recent literature review of publications between 2003 and 2018, only 6 out of 39 studies examined associations between weight gain during infancy and body composition in adult participants from LMICs.[7] Less evidence is available about the role of linear growth (height) on adult body composition, and findings are mixed.[7] There is a need for investigating the independent long-term role of linear growth and relative weight gain across the life course on body composition in young and mid-adulthood; stages in which the accumulation of cardiometabolic risk is higher.

For peer review only

Pooled analyses of COHORTS, a consortium of birth cohorts from five LMICs,[18] showed that higher weight at birth, in infancy, and in mid-childhood were associated with a higher risk of adult overweight (ages 18-31).[13] These pooled analyses also showed that weight at birth and in infancy were stronger predictors of FFM than of FM in adult life, while weight gain in mid-childhood was a stronger predictor of FM than FFM.[13 14] We aim to expand on these previous studies through the examination of the associations of linear growth and relative weight at birth, infancy, childhood, and adolescence with measures of body composition (BMI, FMI, FFMI, FM/FFM ratio, and waist circumference) in young and mid-adulthood, using updated data from COHORTS.

Methods

Study design and participants

For peer review only - http://bmjopen.bmj.com/site/about/guidelines.xhtml

We conducted a secondary data analysis of COHORTS, the Consortium of Health-Oriented Research in Transitional Societies (COHORTS).[18] This consortium comprises six prospective birth cohorts in five study sites: 1) The 1982[19 20] and 1993 Pelotas Birth Cohorts in Brazil;[21] 2) The Institute of Nutrition of Central America and Panama Nutrition Trial and Longitudinal Study in Guatemala;[22] 3)The New Delhi Birth Cohort in India;[23] 4) The Cebu Longitudinal Health and Nutrition Survey in the Philippines;[24] and The Birth to Twenty Plus Cohort in South Africa.[25] Table 1 presents a general description of each birth cohort.

We selected one analytic sample for each study site (Figure 1). Of a total of 27437 participants, we excluded participants with missing body composition in adulthood (n=16029), implausible values of BMI ≤ 12 or ≥ 60 Kg/m^2 and waist circumference ≤ 51 or ≥ 190 cm (n=1), and pregnancy at the moment of the anthropometric assessment (n=135). We also excluded those individuals with missing anthropometric data at birth, childhood, or adolescence (n=6967), implausible values of weight and height considering the WHO Z-scores thresholds (n=168),[26 27] and pregnancy at the adolescence measurement (n=47).

Variable specification

Outcomes: Adult body composition

Study sites applied different standard methods to measure body composition in adult participants such as dual-energy X-ray absorptiometry (DEXA), air-displacement plethysmography (BodPod), deuterium oxide dilution technique, bioimpedance,[19 28-30] and anthropometry (Table 1). Our outcomes measures are: 1) BMI (weight in kg/height in meters^2) as a proxy of body size; 2) FMI to represent the fat compartment adjusted for size (fat mass in kg/height in meters^2); 3) FFMI to represent the lean compartment adjusted for size (fat-free mass in kg/height in meters^2); 4) FM/FMM ratio (a higher ratio indicates a greater increment in the fat mass component); and 5) the waist circumference (centimeters) as a proxy of abdominal adiposity. We transformed these outcomes measures into Z-scores (SD: standard deviation units) to compare effect sizes within strata of site and sex, and among the measures.

For peer review only

Table 1. General characteristics of the six birth cohorts and body composition methods used at each study site

Country (cohort)	Study design	Cohort enrollment	Age at enrollment	Initial sample	Analytic sample	Age & year most recent follow-up	Body composition methods
Brazil (1982 Pelotas Birth Cohort)	Prospective cohort	1982	Birth	5914	674	30 (2012)	BodPod and anthropometry
Brazil (1993 Pelotas Birth Cohort)	Prospective cohort	1993	Birth	5249	827	22.5 (2015)	BodPod and anthropometry
Guatemala (INCAP Nutrition Trial and Longitudinal Study)	Community trial	1969-77	Pregnancy – 7 years	2392	163	45.5 (2015-17)	Deuterium oxide dilution technique and anthropometry
India (NDBC)	Prospective cohort	1969-72	Before pregnancy	8181	681	46 (2016-19)	Bioimpedance and anthropometry
The Philippines (CLHNS)	Prospective cohort	1983-84	Birth	3080	1197	34.5 (2018)	Bioimpedance and anthropometry
South Africa (Birth to Twenty Plus Cohort)	Prospective cohort	1990	Pregnancy	3273	595	22 (2012)	DEXA (Dual-energy X-ray absorptiometry) and anthropometry

INCAP: Institute of Nutrition of Central America and Panama; NDBC: New Delhi Birth Cohort; CLHNS: Cebu Longitudinal Health and Nutrition Survey
BodPod: air-displacement plethysmography

For peer review only

Anthropometry at birth, infancy, childhood, and adolescence

Birthweight was measured in all study sites, while birth length was available only in four of the six study sites (except Brazil 1982 and South Africa). In Brazil, anthropometric data at birth was measured at the hospitals by the research team (Brazil 1993) or by the hospital personnel (Brazil 1982) using pediatric scales.[31] In Guatemala, the study team measured weight within the first 48 hours after birth and length within the first 15 days after the delivery, using a beam balance and a measuring board.[32] In India, trained project staff measured birth and length within the first 72 hours of life using standard instruments.[33] In the Philippines, the 60% of the participants who were born at home were weighted by local birth attendants using hanging scales and the remaining participants were measured in hospitals, and birth length was measured within the first 6 days of life.[34] In South Africa, birthweight measured by hospital personnel was obtained from hospital records.

Trained research staff at each study site followed standard procedures to take anthropometric measures of length and/or height and weight in subsequent follow-ups (childhood and adolescence). Growth measures that represent linear growth (length/height) and mass accretion independent of linear size (relative weight) were measured in different periods at each study site. We identified four common or near ages where anthropometric data was available across all cohorts: 1) birth, 2) infancy (all study sites registered weight and length data at 24 months of age except Brazil 1993 that collected these data at 12 months), 3) childhood (five study sites collected growth data at 48 months of age, but Philippines that collected data at 102 months), and 4) adolescence (all study sites collected anthropometric data at180 months of age). To be able to compare growth measures between study sites, we estimated sex-specific length/height-for-age and weight-for-age Z-scores (HAZ and WAZ, respectively) using the WHO growth standards.[35]

Exposures: Conditional growth (height and relative weight)

Repeated measures of linear growth and relative weight are strongly correlated. To eliminate this correlation, we estimated conditional size measures (standardized residuals) by regressing current HAZ

For peer review only - http://bmjopen.bmj.com/site/about/guidelines.xhtml

on previous HAZ and WAZ measures, and regressing current WAZ on current HAZ and all prior HAZ and WAZ measures, within strata of site and sex. This approach allowed us to identify if children's growth deviates from their expected length/height or weight as estimated based on their own previous growth measurements and the growth of other children. We used birthweight as the anchor to estimate conditional growth measures (height and relative weight) in the main analysis of all cohorts. In separate models, we derived conditional growth measures using birth length as an anchor in the four study studies in which this variable was available (Supplementary Tables 8-13). We estimated conditional height and conditional relative weight in four age intervals across all study sites: 1) prenatal (from conception to birth captured though birthweight and birth length); 2) infancy (birth to 12 months in Brazil 1993, and birth to 24 months in the other study sites); 3) childhood (12-48 months in Brazil 1993, 24-48 months in Brazil 1982, Guatemala, India, and South Africa, and 24-102 months in Philippines); and 4) adolescence (48-180 months in five study sites and 102-180 months in Philippines).

Covariates: We identified potential confounders available at each study site such as birth characteristics (gestational age, birth order), income and/or wealth index of child's household at birth, maternal characteristics (height, age at first childbirth, schooling attainment, marital status), paternal characteristics (age, schooling attainment), reproductive factors (age at menarche), and age in adulthood (Supplementary Table 1).

Statistical analyses

We used R version 4.0 for all statistical analyses. Statistical significance was established at a p-value < 0.05.

Site-specific regression models

We used multiple linear regressions with standardized coefficients to estimate the associations between growth patterns at the four age intervals and adult body composition since all outcomes were continuous

For peer review only

and without major violations of the normal distribution (graphical examination). We built site-specific models stratified by sex given the biological differences in body composition.[36] We ran two sets of models: 1) Unadjusted models for all study sites except Guatemala, in which we minimally adjust for birth year and intervention group considering the initial study design of this cohort;[22] 2) Fully adjusted models controlling for potential confounders available at each study site (Supplementary Table 1). Additionally, we adjusted for adult height in waist circumference models, for age at menarche and teenage childbearing in models of women, and for maternal skin color in both Brazil cohorts as one proxy of socioeconomic status. In Brazil 1993 models, we used survey procedures given that this cohort collected data in subsamples (all children with low-weight at birth and a random sample of other children) at 12 and 48 months of age.[31] After a visual examination of effect sizes from both sets of models at each study site, we observed slight changes in magnitude but not in direction (results not presented). We present the fully adjusted models to account for the confounding effect of this group of covariates.

Pooling Effect Sizes

The cohorts come from different study sites and data were collected in different periods, resulting in heterogeneity among site-specific estimates. We used a random-effects meta-analysis approach to pool effect sizes to account for this underlying heterogeneity. Given that our outcomes were continuous, we used the restricted maximum likelihood estimator (REML) to calculate the between-study heterogeneity (variance of the distribution of true effect sizes).[37] We also applied Knapp-Hartung adjustments in the estimation of confidence intervals of pooled effects. We estimated Cochran's Q with its corresponding p-value to test whether there was heterogeneity between study sites, and calculated the I^2 statistic to quantify the magnitude of between-study heterogeneity. As a rule of thumb an I^2 equal to 25% is interpreted as low heterogeneity, 50% as moderate heterogeneity, and 75% as substantial heterogeneity.[37] We used the Metafor package in R to run these analyses.

Multiple imputation

For peer review only

To deal with missing data, we built sex-specific multiple imputation models at each study site. Missing data patterns are presented in Supplementary Table 1. We assumed that the underlying missing pattern was missing at random (MAR). In brief, we used the R package Multivariate Imputation by Chained Equations (MICE) to generate different multiple imputed datasets with a maximum of 50 iterations. We estimated the number of imputations based on the highest percentage of missingness within strata of site and sex. In our imputation models, we included all covariates used in the main analyses except the outcome variables. We used the "with and pool" functions to run the main analyses in each imputed dataset and to combine the estimates into a single result with its corresponding standard errors and confidence intervals.

Results

We analyzed data from a combined analytic sample of 4137 participants with complete and valid information of body composition in adulthood and anthropometry across the life course (Figure 1). The comparison of general characteristics between participants included in the final analytic samples versus those excluded only showed significant differences in a couple of variables in selected cohorts (Supplementary Table 2). General characteristics of the six cohorts, and birth, childhood and adulthood characteristics of men and women at each study site are presented in Tables 1 and 2. Participant's average adult age ranged from 22 years in Brazil 1993 and South Africa to 45-46 years in Guatemala and India. India had the lowest mean birth weight compared with other study sites. The proportion of excess weight (BMI ≥ 25 kg/m^2) among men ranged from 16.0% in South Africa to 73.2% in India, and among women ranged from 44.5% in Brazil 1993 to 85.4% in Guatemala. Participants from Guatemala, India, and Brazil 1982 had the highest FMI (women above 10 kg/m^2 and men above 7 kg/m^2). FFMI ranged from 15.5 to 20.2 kg/m^2 in men, and from 13.7 to 17.7 kg/m^2 in women.

For peer review only

Table 2. Characteristics of participants included in the analysis, stratified by study site and sex[1]

	Brazil 1982		Brazil 1993		Guatemala		India		The Philippines		South Africa	
	Men (n=344)	Women (n=330)	Men (n=375)	Women (n=452)	Men (n=67)	Women (n=96)	Men (n=426)	Women (n=255)	Men (n=656)	Women (n=541)	Men (n=287)	Women (n=308)
**Birth variables**												
Gestational age (weeks)	39.4 (1.8)	39.5 (1.8)	39.6 (2.2)	39.7 (2.3)	39.3 (3.0)	39.1 (3.6)	38.8 (2.4)	39.1 (2.3)	38.7 (2.1)	38.8 (2.1)	38.0 (1.9)	37.8 (2.0)
Birth length (cm)	NA	NA	49.4 (2.3)	48.4 (2.1)	49.6 (2.6)	48.5 (2.2)	48.7 (2.0)	47.9 (2.1)	49.3 (2.0)	48.7 (2.0)	NA	NA
Birth weight (kg)	3.3 (0.6)	3.2 (0.5)	3.3 (0.5)	3.1 (0.5)	3.1 (0.5)	3.0 (0.5)	2.9 (0.4)	2.7 (0.4)	3.0 (0.4)	3.0 (0.4)	3.1 (0.5)	3.0 (0.5)
Birth order (%)												
1	143 (41.6%)	120 (36.4%)	147 (37.1%)	145 (33.1%)	12 (17.9%)	14 (14.6%)	46 (17.2%)	26 (16.5%)	149 (22.7%)	120 (22.2%)	105 (36.6%)	118 (38.3%)
2	98 (28.5%)	85 (25.8%)	107 (27.2%)	130 (29.6%)	13 (19.4%)	12 (12.5%)	59 (22.1%)	37 (23.4%)	132 (20.1%)	120 (22.2%)	86 (30.0%)	93 (30.2%)
3	54 (15.7%)	64 (19.4%)	63 (15.8%)	99 (22.6%)	6 (9.0%)	15 (15.6%)	53 (19.9%)	31 (19.6%)	130 (19.8%)	103 (19.0%)	48 (16.7%)	51 (16.6%)
≥4	49 (14.2%)	61 (18.5%)	79 (20.0%)	64 (14.6%)	36 (53.7%)	55 (57.3%)	109 (40.8%)	64 (40.5%)	245 (37.3%)	198 (36.6%)	48 (16.7%)	46 (14.9%)
**Childhood variables[2]**												
HAZ in infancy	-0.6 (1.2)	-0.5 (1.2)	-0.1 (1.3)	-0.0 (1.2)	-3.1 (1.2)	-2.8 (1.0)	-2.0 (1.1)	-1.9 (1.1)	-2.5 (1.1)	-2.4 (1.1)	-1.4 (1.0)	-1.1 (1.0)
HAZ in childhood	-0.5 (1.1)	-0.6 (1.1)	-0.1 (1.2)	-0.2 (1.1)	-2.3 (0.9)	-2.4 (0.9)	-1.9 (1.0)	-2.1 (1.0)	-2.1 (0.9)	-2.1 (0.9)	-1.0 (0.9)	-0.9 (0.9)
HAZ in adolescence	-0.1 (1.0)	-0.3 (0.9)	0.5 (1.2)	0.5 (1.0)	-2.0 (0.9)	-1.9 (0.7)	-1.2 (1.1)	-1.3 (0.8)	-1.9 (0.8)	-1.9 (0.8)	-0.7 (1.0)	-0.5 (0.9)
WAZ at infancy	0.2 (1.1)	0.2 (0.1)	0.3 (1.2)	0.2 (1.1)	-1.9 (1.0)	-1.6 (1.0)	-1.5 (1.1)	-1.5 (1.0)	-1.7 (0.9)	-1.7 (1.0)	-0.7 (1.1)	-0.4 (0.9)
WAZ in childhood	0.2 (1.0)	0.0 (1.0)	0.6 (1.2)	0.4 (1.1)	-1.4 (0.7)	-1.4 (0.8)	-1.3 (0.9)	-1.5 (0.9)	-2.0 (1.0)	-1.9 (0.9)	-0.5 (0.9)	-0.5 (0.9)
BAZ in adolescence	0.3 (1.1)	0.3 (1.1)	0.3 (1.2)	0.3 (1.1)	-0.9 (0.8)	0.1 (0.7)	-1.3 (1.2)	-0.6 (1.0)	-1.0 (1.0)	-0.7 (1.0)	-0.5 (1.1)	0.2 (1.2)
**Covariates**												
Maternal age at childbirth (years)	26.5 (6.4)	26.8 (6.5)	26.3 (6.5)	26.4 (6.3)	26.7 (7.9)	27.7 (7.2)	26.9 (5.9)	26.9 (5.5)	26.6 (6.2)	26.4 (6.0)	26.0 (6.5)	25.7 (6.4)
Maternal height (cm)	157.1 (6.4)	156.7 (6.2)	160.2 (6.5)	160.0 (6.9)	148.3 (4.7)	148.7 (5.1)	151.9 (5.8)	151.8 (5.1)	150.4 (4.8)	150.2 (5.0)	158.2 (6.4)	158.6 (6.0)
Maternal schooling (years)	6.7 (4.1)	6.8 (4.3)	7.0 (3.5)	6.7 (3.4)	1.3 (1.5)	1.3 (1.5)	5.7 (4.5)	5.6 (4.4)	6.9 (3.3)	6.7 (3.1)	9.6 (2.5)	9.7 (2.7)
Paternal age (years)	52.0 (7.0)	52.5 (7.0)	NA	NA	32.7 (9.4)	32.9 (8.4)	NA	NA	29.1 (6.8)	28.9 (6.8)	NA	NA
Paternal schooling (years)	6.9 (4.3)	7.1 (4.0)	6.8 (3.6)	6.8 (3.4)	2.0 (2.0)	1.8 (2.0)	10.4 (5.0)	11.4 (4.7)	7.0 (3.5)	6.7 (3.2)	10.6 (2.2)	10.7 (2.6)
Childhood wealth index (score)	3.4 (1.1)	3.5 (1.1)	NA	NA	-0.3 (0.6)	-0.2 (0.8)	4.5 (1.1)	4.5 (1.1)	3.6 (2.4)	3.5 (2.3)	4.1 (1.4)	4.1 (1.3)

For peer review only

Age at menarche	--	12.2 (1.2)	--	--	--	13.4 (1.4)	--	13.7 (1.2)	--	13.2 (1.0)	--	12.7 (1.2)
**Adult variables**												
Height (cm)	174.9 (7.1)	161.9 (6.2)	174.8 (7.6)	160.8 (6.6)	164.7 (5.7)	152.0 (4.8)	169.9 (6.8)	154.5 (5.5)	162.8 (5.6)	150.9 (5.5)	171.5 (6.2)	159.7 (6.4)
Weight (kg)	82.8 (16.3)	70.1 (16.6)	75.8 (16.1)	66.4 (15.6)	71.4 (14.3)	69.4 (11.8)	80.6 (15.0)	70.0 (12.5)	66.1 (12.8)	57.3 (11.9)	62.9 (11.3)	64.6 (14.9)
BMI (kg/m^2)	27.0 (4.8)	26.7 (6.1)	24.6 (4.7)	25.5 (5.9)	26.3 (4.8)	30.0 (4.7)	27.9 (4.7)	29.3 (4.8)	24.9 (4.4)	25.2 (5.0)	21.4 (3.5)	25.3 (5.6)
Excess weight[3]	215 (62.5%)	171 (51.8%)	160 (40.4%)	195 (44.5%)	42 (62.7%)	82 (85.4%)	312 (73.2%)	208 (81.6%)	297 (45.3%)	256 (47.3%)	46 (16.0%)	142 (46.1%)
Waist circumference (cm)	89.9 (11.5)	80.8 (11.7)	82.2 (10.8)	77.3 (11.6)	93.7 (10.8)	102.8 (10.7)	100.6 (12.0)	91.8 (10.5)	82.1 (10.8)	80.8 (11.5)	75.8 (8.6)	82.4 (13.5)
Fat mass index (FMI, kg/m^2)	7.1 (3.6)	10.5 (4.7)	5.3 (3.7)	9.6 (4.5)	7.7 (2.8)	12.7 (3.1)	7.7 (2.8)	11.6 (3.4)	5.5 (2.5)	9.0 (3.5)	4.0 (2.1)	9.6 (3.9)
Fat-free mass index (FFMI, kg/m^2)	19.9 (2.1)	16.2 (1.9)	19.2 (1.9)	15.8 (1.9)	18.5 (2.6)	17.3 (2.2)	20.2 (2.2)	17.7 (1.7)	19.3 (2.0)	16.1 (1.4)	15.5 (1.8)	13.7 (1.9)
Fat mass/Fat-free mass ratio	0.4 (0.2)	0.6 (0.2)	0.3 (0.2)	0.6 (0.2)	0.4 (0.1)	0.7 (0.1)	0.4 (0.1)	0.6 (0.2)	0.3 (0.1)	0.5 (0.2)	0.3 (0.1)	0.7 (0.2)

[1] Values are means (SD) or n (percentages). Sample sizes (n) refer to participants with non-missing data for outcomes (adult body composition), and non-missing data for exposures (growth measures). NA, not available data.

[2] HAZ, height-for-age Z-score; WAZ, weight-for-age Z-score; BAZ, BMI-for-age Z-score; BMI, body mass index. HAZ and WAZ measures in infancy were measured at 24 months of age in all sites except Brazil 1993, where children were measured at 12 months of age. Childhood corresponds to 48 months for all study sites but Philippines, where anthropometric measures were obtained at 102 months of age. Measures of adolescence were obtained at 15 years of age (180 months) in all study sites.

[3] Excess weight defined as a BMI ≥ 25 kg/m^2

For peer review only

Conditional relative weight and adult body composition

Table 3 shows the pooled adjusted associations of conditional growth with adult FMI, FFMI and FM/FFM. Birth weight and conditional relative weight in infancy, childhood, and adolescence were positively associated with FMI and FFMI in adulthood. A higher birth weight was a predictor of higher FMI (men: 0.07 [95%CI: 0.00,0.14]; women: 0.09 [95%CI: 0.00,0.18]) and FFMI (men: 0.11 [95%CI: 0.04, 0.18]; women: 0.12 [95%CI: 0.05, 0.19]). Birthweight was not significantly associated with adult FM/FFM ratio. We found that an increment of one SD in conditional relative weight in infancy was associated with an increment of 0.2-0.3 SD in adult FMI, while we observed that for every SD increment in conditional weight in childhood or adolescence there was a 0.4-0.5 SD increase in FMI in adulthood. Similarly, the associations of relative weight gain with adult FFMI and FM/FFM were positive and stronger as boys and girls grew older. Increments in conditional relative weight by one SD in infancy, childhood, and adolescence were associated with higher FFMI by 0.3, 0.4-0.5, and 0.4 SD units in adult life, respectively. Conditional relative weight in infancy, childhood and adolescence were associated with a higher FM/FMM ratio with increments ranging from 0.18 to 0.43 for every SD in conditional weight.

For peer review only

Table 3. Pooled adjusted associations between weight at birth, conditional growth (height and relative weight) in infancy, childhood, and adolescence with adult fat mass index, fat-free mass index and fat mass/fat-free mass ratio, stratified by sex[1]

	FMI (SD units)			FFMI (SD units)			FM/FFM (SD units)		
**Conditional growth, z-scores[2]**	β (95%CI)	I^2 statistic (%)[3]	Cochran's Q test (P value)	β (95%CI)	I^2 statistic (%)	Cochran's Q test (P value)	β (95%CI)	I^2 statistic (%)[3]	Cochran's Q test (P value)
**Men (n = 2155)**									
Birth weight	0.07* (0.00,0.14)	44.9	0.128	0.11* (0.04,0.18)	46.6	0.073	0.06 (-0.04,0.15)	67.8	0.023
Conditional relative weight in infancy	0.23* (0.19,0.27)	0.0	0.866	0.30* (0.23,0.37)	22.6	0.303	0.18* (0.12,0.23)	0.0	0.685
Conditional relative weight in childhood	0.40* (0.26,0.55)	62.3	0.019	0.50* (0.33,0.66)	75.9	0.001	0.31* (0.16,0.47)	66.1	0.009
Conditional relative weight in adolescence	0.39* (0.27,0.50)	78.2	0.001	0.43* (0.32,0.55)	82.3	<0.001	0.31* (0.18,0.44)	78.7	0.001
Conditional height in infancy	0.09* (0.03,0.14)	23.7	0.244	0.09 (0,0.18)	68	0.004	0.07* (0.02,0.12)	27.9	0.309
Conditional height in childhood	0.08 (-0.01,0.18)	41.4	0.161	0.05 (-0.06,0.17)	57.2	0.044	0.09* (0.00,0.17)	27.7	0.322
Conditional height in adolescence	0.02 (-0.07,0.12)	13.2	0.207	0.00 (-0.08,0.08)	12.6	0.353	0.02 (-0.08,0.13)	24.2	0.143
**Women (n = 1982)**									
Birth weight-for-age	0.09* (0.00,0.18)	61.4	0.03	0.12* (0.05,0.19)	18.3	0.042	0.06 (-0.04,0.16)	71.4	0.007
Conditional relative weight in infancy	0.26* (0.19,0.33)	0.0	0.526	0.30* (0.21,0.39)	50.3	0.104	0.20* (0.09,0.31)	30.1	0.118
Conditional relative weight in childhood	0.46* (0.21,0.72)	84.3	<0.001	0.42* (0.17,0.66)	92.2	<0.001	0.37* (0.07,0.67)	87.8	0
Conditional relative weight in adolescence	0.52* (0.44,0.61)	51.2	0.091	0.39* (0.13,0.66)	95	<0.001	0.43* (0.38,0.48)	0	0.745
Conditional height in infancy	0.11* (0.07,0.16)	12.9	0.547	0.09 (-0.01,0.19)	77.6	0.002	0.10* (0.03,0.16)	36.2	0.196
Conditional height in childhood	0.14* (0.04,0.24)	31	0.288	0.12 (-0.14,0.37)	89.9	<0.001	0.16* (0.08,0.25)	15	0.486
Conditional height in adolescence	0.01 (-0.13,0.15)	55.5	0.063	0.06 (-0.16,0.27)	89.2	<0.001	-0.01 (-0.19,0.17)	71.3	0.003

[1] Values are standardized linear regression coefficients (*β*s and 95% CIs). FMI, fat mass index; FFMI, fat-free mass index. FM/FFM, fat mass/fat-free mass ratio. In adjusted analyses we controlled for birth characteristics (gestational age, birth order), maternal characteristics (height, age at childbirth, schooling, marital status), paternal schooling, income and/or wealth index of child's household at birth, and age in adulthood. In women' models, we adjusted for age of menarche and teenage childbearing. Additionally, we controlled for maternal skin color in both Brazil cohorts and adjusted for birth year and intervention group (village fixed effects) in Guatemala analysis. * P value < 0.05

[2] We used relative birth weight as the anchor in all conditional models given that this measure was available across all study sites. In the supplementary section, we present similar models using birth length as the anchor in the sites that collected this measure.

[3] As a rule of thumb I^2 = 25% is interpreted as low heterogeneity, 50% moderate heterogeneity, and 75% substantial heterogeneity

For peer review only

The between-study heterogeneity assessment showed low or moderate heterogeneity across almost all associations of conditional growth with FMI and FM/FFM, except conditional relative weight in childhood among women (I^2=84.3%) and conditional relative weight in adolescence among men (I^2=78.2%). The between-study heterogeneity assessment of FFMI models showed substantial variability in estimates of conditional relative weight in childhood and adolescence among women and men, and conditional height in childhood and adolescence among women ($I^2 \geq 75\%$) (Table 3).

Site-specific estimates were consistent in magnitude and direction. Birth weight was positively associated with adult FMI in men and women from India and the Philippines, and in women from Brazil 1993. Conditional relative weights in infancy, childhood, and adolescence were positively associated with adult FMI among men and women across all cohorts (Supplementary Table 3). Site-specific analyses showed that birthweight was a predictor of a higher adult FFMI among men and women from four out of the six cohorts (Brazil 1982, India, the Philippines, and South Africa), and among women from Brazil 1993 and Guatemala. Across all cohorts but Guatemala, a higher relative weight gain in childhood was significantly associated with a higher FFMI. Conditional relative weight in adolescent was a strong and significant predictor of a higher FFMI in adult life among all study sites and both sexes (Supplementary Table 4). Birthweight was positively and significantly associated with FM/FFM among men and women from India and the Philippines, and women from Brazil 1993. Conditional relative weights in infancy, childhood, and adolescence were positively associated with FM/FFM across all study sites with a few exceptions (Supplementary Table 5).

Table 4 shows the pooled adjusted associations of conditional growth with adult BMI and waist circumference. Birth weight and all conditional relative weight measures were positively associated with BMI and waist circumference among adult men and women. Effect sizes at birth were small but significant predictors of BMI and waist circumference. Associations strengthened with age at measurement; for example, men's BMI increased by 0.29 [95%CI: 0.24, 0.35], 0.50 [95%CI: 0.37, 0.63]

For peer review only

and 0.47 [95%CI: 0.35, 0.60] SD units per each SD in conditional relative weight in infancy, childhood, and adolescence, respectively. Similar patterns were observed in women. Relative weight gains in infancy and childhood were associated with increments in waist circumference ranging from 0.25-0.26 and 0.40-0.43 SD among adult men and women, respectively. Increments in relative weight during adolescence predicted increments in waist circumference by 0.39 SD in men and 0.49 SD units in women. The between-study heterogeneity was low or moderate for most of the associations, except some associations of conditional relative weight in childhood and adolescence (heterogeneities above 75%) (Table 4).

Site-specific analyses of BMI and waist circumference showed similar effect sizes in direction and magnitude as pooled estimates. Relative weight gains in infancy, childhood, and adolescence were predictors of higher BMI and waist circumference in adulthood across study sites, with a few exceptions (Supplementary Tables 6 and 7).

For peer review only

Table 4. Pooled adjusted associations between weight at birth, conditional growth (height and relative weight) in infancy, childhood, and adolescence with adult body mass index and waist circumference, stratified by sex[1]

	BMI (SD units)			Waist Circumference (SD units)		
**Conditional growth, z-scores[2]**	β (95%CI)	I^2 statistic (%)[3]	Cochran's Q test (P value)	β (95%CI)	I^2 statistic (%)	Cochran's Q test (P value)
**Men (n = 2155)**						
Birth weight	0.10* (0.05,0.14)	8.0	0.520	0.07* (0.03,0.12)	0.0	0.621
Conditional relative weight in infancy	0.29* (0.24,0.35)	0.0	0.616	0.26* (0.19,0.33)	15.0	0.359
Conditional relative weight in childhood	0.50* (0.37,0.63)	59.3	0.042	0.43* (0.34,0.52)	0.0	0.554
Conditional relative weight in adolescence	0.47* (0.35,0.60)	85.8	<0.001	0.39* (0.27,0.52)	80.4	<0.001
Conditional height in infancy	0.10* (0.03,0.16)	36.9	0.099	0.12* (0.03,0.21)	48.8	0.088
Conditional height in childhood	0.08 (-0.04,0.19)	57.5	0.046	0.11 (-0.04,0.26)	56.7	0.043
Conditional height in adolescence	0.01 (-0.07,0.09)	0.0	0.322	0.08* (0.00,0.16)	0.0	0.632
**Women (n = 1982)**						
Birth weight-for-age	0.12* (0.03,0.2)	52.0	0.046	0.08* (0.01,0.16)	0.1	0.253
Conditional relative weight in infancy	0.30* (0.24,0.36)	0.0	0.671	0.25* (0.19,0.31)	0.0	0.662
Conditional relative weight in childhood	0.53* (0.31,0.75)	78.9	0.001	0.40* (0.15,0.64)	80.4	0.001
Conditional relative weight in adolescence	0.58* (0.47,0.69)	70.0	0.005	0.49* (0.41,0.56)	32.7	0.254
Conditional height in infancy	0.12* (0.08,0.16)	0.0	0.702	0.07 (-0.07,0.2)	35.9	0.144
Conditional height in childhood	0.12 (-0.01,0.25)	53.3	0.066	0.14* (0.05,0.23)	0.0	0.843
Conditional height in adolescence	0.01 (-0.1,0.13)	37.0	0.224	-0.05 (-0.28,0.19)	45.4	0.123

[1] Values are standardized linear regression coefficients (*β*s and 95% CIs). BMI, body mass index. In adjusted analyses we controlled for birth characteristics (gestational age, birth order), maternal characteristics (height, age at childbirth, schooling, marital status), paternal schooling, income and/or wealth index of child's household at birth, and age in adulthood. In women' models, we adjusted for age of menarche and teenage childbearing. Additionally, we controlled for maternal skin color in both Brazilian cohorts and adjusted for birth year and intervention group (village fixed effects) in Guatemala analysis. * P value < 0.05

[2] We used relative birth weight as the anchor in all conditional models given that this measure was available across all study sites. In the supplementary section, we present similar models using birth length as the anchor in the sites that collected this measure

[3] As a rule of thumb: $I^2 = 25\%$ is interpreted as low heterogeneity, 50% moderate heterogeneity, and 75% substantial heterogeneity

For peer review only

401 ***Conditional height and adult body composition***

402

403 Conditional height in infancy was positively associated with FMI and FM/FFM ratio in adult life, but
404 effect sizes were small (around 0.1). None of the conditional height variables were significantly
405 associated with adult FFMI (Table 3). A high conditional height in infancy was a significant but weak
406 predictor of higher adult BMI among men and women, and waist circumference among women
407 (increments of 0.10-0.12 SD units) (Table 4). Conditional height in childhood was significantly but
408 weakly associated with women's waist circumference (0.14 [95%CI: 0.05,0.23]) whilst conditional height
409 in adolescence was positively associated with men's waist circumference (0.08 [95%CI: 0.00,0.16]). Site-
410 specific results are shown in Supplementary Tables 3-7.

411

412 We used conditionals derived from birth length as the anchor in models in four of the study sites
413 (Supplementary Tables 8 – 13). In pooled analysis, we found that birth length was a weak but statistically
414 significant predictor of higher FFMI and BMI in adult women (Supplementary Tables 8 and 9). Other
415 effect sizes from these models were consistent in direction and magnitude with effect sizes observed in
416 the main models (birth weight as anchor). Site-specific analysis using birth length as anchor are presented
417 in Supplementary Tables 10-13.

418

419 We also examined the correlation between body composition outcomes with adult height. The three
420 indices (BMI, FMI, and FFMI) were not significantly correlated with height across all study sites and
421 sexes (correlation coefficients ranged from -0.15 to 0.10). The correlation coefficients of waist
422 circumference with adult height were significant but weak (0.15-0.26) (Supplementary Table 14).

423

424 **Discussion**

425

For peer review only

In a pooled analysis of six prospective birth cohorts from five LMICs, we found that a higher birthweight and higher relative weight gains in infancy, childhood, and adolescence were associated with higher FFMI, FMI, BMI and waist circumference in adult life. Additionally, we observed that conditional relative weight from infancy to adolescence was associated with a greater FM/FMM ratio. Effect sizes of all outcomes were strongest in childhood and adolescence, almost twice the magnitude of effect sizes in infancy. Our findings showed that prenatal linear growth was a positive predictor of FFMI and BMI only among adult women. In infancy, a higher conditional height was a significant but weak predictor of higher FMI and BMI among adult men and women, and higher waist circumference among women. A higher conditional height in childhood was positively but weakly associated with FMI and waist circumference among women. A faster linear growth in adolescence was a weak predictor of a higher waist circumference among adult men.

This updated analysis confirms previous results from COHORTS[13 14] and similar site-specific studies[28 33 38] at showing that birthweight is a predictor of adult FFMI and FMI. Thus, our findings showed the independent and long-lasting role of prenatal growth (weight gain mainly) on body composition and size in young and middle-age adulthood. There are differences in effect sizes when comparing our estimates of FMI and FFMI with previous COHORTS' analysis because we adjusted by adult height, used a different approach to combine site-specific estimates, participants were older, and possibly due to the decreased association of birthweight with adult measures across time. The positive and significant associations between prenatal and postnatal relative weight gains with all outcomes in young and mid-adulthood suggest that relative weight is a predictor of overall body size than body composition. A recent review of literature highlighted a similar conclusion in relation to rapid weight gain in infancy (0-2 years of age) with body size in childhood, adolescence, and adulthood.[7] We expand on that conclusion by showing that excessive relative weight gain beyond 2 years of age is also a predictor of overall size in young and middle-age adulthood. Additionally, we infer that a higher FFMI reflects the accretion in FFM as a body response (expansion of bone and muscle mass) to support higher weight,[39 40] since on average COHORTS

For peer review only

452 participants had overweight or obesity in adulthood. This inference is also supported by the finding that
453 conditional relative weight in infancy, childhood and adolescence were associated with a greater FM/FFM
454 ratio, indicating that the increments in FM were faster than the increments in FMM. Thus, higher weight
455 gains during these age intervals might be associated with a greater risk of adiposity in adult life.

456

457 Compared to previous combined analysis of COHORTs, we examined for the first time the interval from
458 childhood to adolescence. Our findings showed that relative weight gain in adolescence was associated
459 with higher adult adiposity (FMI, FM/FFM ratio, and waist circumference) in consistency with earlier
460 studies in LMICs.[13 14 28] Studies in HIC, like an analysis of the Fels Longitudinal Study showed similar
461 trends. This study found that timing and velocity of BMI in childhood, adolescence, and post-adolescence
462 were predictors of higher BMI and adiposity in adulthood (ages 35-45), being adolescence and post-
463 adolescence the strongest predictors of adult adiposity.[41] Whereas we observed similar effect sizes in
464 childhood and adolescence suggesting that both periods are equally important on predicting the risk of
465 higher adiposity in adult life. In a deeper understanding of the predicting role of growth on adult
466 adiposity, recent analyses of Brazil 1982[42] and South Africa[28] cohorts found that conditional relative
467 weight gains across different stages of the life course were significant predictors of both visceral and
468 subcutaneous fat at ages 30 and 22, respectively.[42]

469

470 A combination of biological, environmental, behavioral, and sociodemographic factors is associated with
471 weight gain and adiposity in childhood and adolescence that may not only persist later in life but might
472 undermine the positive role of a healthy birthweight and linear growth early in life. For instance, the
473 medium to strong tracking of child and adolescent BMI into adult adiposity,[8 11] prenatal and postnatal
474 factors,[43] reproductive characteristics of women (earlier age at menarche, short periods between age at
475 menarche and age at first pregnancy)[44], and changes in lifestyle patterns (diets of obesogenic
476 environments, a decline in physical activity, and an increment in sedentary activities) as consequence of

For peer review only

477 the nutrition transition and transformations of the food systems, home and work environments in LMICs during the last decades.[45]

Fewer studies have examined the role of linear growth on later body composition and results are mixed.[7] Our results showed that linear growth in infancy was a significant but weak predictor of adiposity in adult life (FMI and BMI in men and women, and waist circumference in women). These findings are also consistent with previous reports of COHORTs[13] and South Africa birth cohort[28], but our estimates were smaller given the adjustment by adult height. Our findings showed that linear growth was not a predictor of adult FFMI, which suggests that linear growth is a predictor of adult height rather than FFM. In contrast, an earlier study in South Africa showed that linear growth across 5 age intervals (from 0-22 years of age) predicted a higher FFM in young adulthood, [28] and an analysis conducted in Guatemala before the start of obesity epidemic found that linear growth was associated with higher FFM in adolescence.[46]

Some limitations of our analysis include: 1) attrition as one source of selection bias given the long-lasting follow-up of these birth cohorts; 2) residual confounding since we could not control for covariates such as diet, reproductive and life style factors; 3) missing data of covariates (ranges between 0.1-41%), for which we assumed MAR and applied multiple imputation techniques. In the imputation models we did not include outcomes variables which might have reduced the strength of the associations; and 4) as expected there was substantial between-study heterogeneity, mostly in associations of conditional relative weight in childhood and adolescence. Some reasons that might explain the observed heterogeneity, beyond the baseline differences in places and contexts, include: a) the age intervals definition was guided by data availability, as not all children were measured at the same intervals; b) differences in sample sizes across study sites given that we had to restrict the analysis to participants with complete anthropometric data in the four age intervals to be able to estimate conditional growth measures; c) differences in the techniques and instruments to measure body composition which might be a source of information bias

For peer review only

(non-differential misclassification). In this regard, our research question intended to study the absolute increase in body composition not the relative estimate, and we assumed that all techniques, independent of the method and units used, ranked people in the same way. Additionally, we transformed all body composition measurements into SD units in each cohort and estimated standardized betas to be able to compare estimates between sites. To minimize the previous limitations, we used the random meta-analysis approach that not only considers the underlying heterogeneity when pooling estimates but also allowed us to control for differences in sample sizes across study sites.

Among the strengths of our analyses, we highlight the prospective and long follow-up of these six birth cohorts in LMICs. We used large sample sizes (except Guatemala), and analyzed two compartments of body composition (beyond BMI) that were measured using gold standards and/or validated techniques. We studied the independent association of linear growth and weight gain with body composition through the use of conditional growth measures, which allowed us to break the high correlation between weight and height and to study the independent role of linear growth and relative weight gain in specific age intervals. We selected the best model at each study site, fully adjusted by multiple confounders, and applied a random effects meta-analysis that by default considers the heterogeneity between study sites.

We are cautious with the generalizability of our results since we used selected birth cohorts, the study design of cohorts is not intended to be representative of countries were information was collected, and there are differences (social, demographic, economic, political, cultural) in contexts and periods in which each cohort started. However, the consistency in magnitude and direction of effect sizes across study-sites (six birth cohorts from five different cities around the world), indicates that our findings are robust and suggests that the long-term role of growth on body composition might apply to individuals from other LMICs with similar characteristics.

For peer review only

In summary, our findings imply that: 1) growth in the first 1,000 days of life has a small but long-lasting association with adult body composition; 2) childhood and adolescence are key periods to implement targeted interventions to prevent adult obesity given the strongest associations between relative weight gain after 2 years of age with body size and composition in adulthood; and 3) public health interventions should aim to promote an adequate linear growth in early life and a healthy weight gain throughout the life course to prevent the excess of adiposity in adulthood, as a step to avert the early onset of adverse cardiometabolic outcomes.

Acknowledgments We thank to all COHORTS participants and study teams. In Guatemala, we acknowledge Paul Melgar, the late field director of the INCAP Nutrition Trial and Longitudinal Study.

Contributors NEP and ADS conceived the idea. NEP, LSA, RM, SAP, MRZ, and ADS were involved in the conceptualization of this analysis. LSA, MRZ, SKB, SAB, DBC, MFKL, BLH, NPL, MM, AMBM, SAN, LHN, LMR, HSS, FCW, ADS, and COHORTS group led and/or participated in data collection. NEP drafted the analysis plan, conducted the data management and statistical analysis, wrote the first draft of the manuscript, and incorporated edits along the review process. All coauthors reviewed and provided insightful comments to the analysis and manuscript drafts. All authors approved the final report.

Funding: The Bill and Melinda Gates Foundation (OPP1164115) funded the most recent wave of data collection in Guatemala, Philippines, and South Africa, and data management and analysis in Brazil. Data collection in Brazil was funded by the Wellcome Trust (086974/Z/08/Z). The New Delhi Birth Cohort has also received funding from the Indian Council of Medical Research (50/1-3/TF/05-NCD-II; 3/1/2/2/15-RCH; 5/10/FR/10/2019-RCH; 5/4/8-7/2019-NCD-II), the Department of Biotechnology (BT/PR3874/MED/97/1/2011; BT/PR5317/FNS/20/552/2012), the United States National Center for Health Statistics (PL-480, RESEARCH PROJECT 0-1-658-2), and the British Heart Foundation (UKPG/05/046). South Africa (Birth to Thirty) funders are the South African Medical Research Council, Wellcome Trust (UK, 077210/Z/05/Z; 092097/Z/10/Z), University of the Witwatersrand and the DSI-

For peer review only

NRF Centre of Excellence in Human Development. NEP was partially supported by a Fulbright-Colciencias fellowship.

Competing interests None declared

Patient and public involvement Patients and/or the public were not involved in the design, or conduct, or reporting, or dissemination plans of our research

Patient consent for publication Not applicable

Ethics approval and consent to participate All cohorts obtained ethics approvals for data collection from the corresponding Institutional Review Boards at each study site (Brazil, Federal University of Pelotas, reference number 1.250.366; Guatemala, Institute of Nutrition for Central America and Panama, reference number CIE-REV-072-2017; India, Sitaram Bhartia Institute of Science and Research, reference numbers SBISR/2012/002, SBISR/RES1/3/2012, SBISR/IEC/2014/001, IEC/SBISR/2015/1, and FL/SBISR/IEC/2019–01; Philippines, Research Ethics Committee at University of San Carlos, reference number 006/2018-01-borja; South Africa, Human Research Ethics Committee at University of Witswatersrand, reference number M180225). Parents or guardians of participants granted their written or verbal consent to participate at baseline and in childhood/adolescence follow-ups, while in adulthood participants gave their own consent. The present analysis was approved by the Institutional Review Board of Emory University (IRB number 95960).

Provenance and peer review Not commissioned; externally peer reviewed.

Data availability statement Data will be available upon reasonable request to the principal investigators at each study site.

For peer review only

References

1. (NCD-RisC) NRFC. Worldwide trends in diabetes since 1980: a pooled analysis of 751 population-based studies with 4.4 million participants. *Lancet* 2016;387(10027):1513-30. doi: 10.1016/s0140-6736(16)00618-8 [published Online First: 2016/04/12]

2. (NCD-RisC) NRFC. Worldwide trends in blood pressure from 1975 to 2015: a pooled analysis of 1479 population-based measurement studies with 19·1 million participants. *Lancet* 2017;389(10064):37-55. doi: 10.1016/s0140-6736(16)31919-5 [published Online First: 2016/11/20]

3. (NCD-RisC) NRFC. Trends in adult body-mass index in 200 countries from 1975 to 2014: a pooled analysis of 1698 population-based measurement studies with 19·2 million participants. *Lancet* 2016;387(10026):1377-96. doi: 10.1016/s0140-6736(16)30054-x [published Online First: 2016/04/27]

4. Ezzati M, Pearson-Stuttard J, Bennett JE, et al. Acting on non-communicable diseases in low- and middle-income tropical countries. *Nature* 2018;559(7715):507-16. doi: 10.1038/s41586-018-0306-9 [published Online First: 2018/07/27]

5. Barker DJ. The developmental origins of well-being. *Philos Trans R Soc Lond B Biol Sci* 2004;359(1449):1359-66. doi: 10.1098/rstb.2004.1518 [published Online First: 2004/09/07]

6. Wells JC, Chomtho S, Fewtrell MS. Programming of body composition by early growth and nutrition. *Proc Nutr Soc* 2007;66(3):423-34. doi: 10.1017/s0029665107005691 [published Online First: 2007/07/20]

7. Woo JG. Infant Growth and Long-term Cardiometabolic Health: a Review of Recent Findings. *Curr Nutr Rep* 2019;8(1):29-41. doi: 10.1007/s13668-019-0259-0 [published Online First: 2019/02/08]

8. Umer A, Kelley GA, Cottrell LE, et al. Childhood obesity and adult cardiovascular disease risk factors: a systematic review with meta-analysis. *BMC Public Health* 2017;17(1):683. doi: 10.1186/s12889-017-4691-z [published Online First: 20170829]

9. Lloyd LJ, Langley-Evans SC, McMullen S. Childhood obesity and adult cardiovascular disease risk: a systematic review. *International journal of obesity (2005)* 2010;34(1):18-28. doi: 10.1038/ijo.2009.61 [published Online First: 20090512]

10. Lloyd LJ, Langley-Evans SC, McMullen S. Childhood obesity and risk of the adult metabolic syndrome: a systematic review. *International journal of obesity (2005)* 2012;36(1):1-11.

11. Singh AS, Mulder C, Twisk JW, et al. Tracking of childhood overweight into adulthood: a systematic review of the literature. *Obes Rev* 2008;9(5):474-88. doi: 10.1111/j.1467-789X.2008.00475.x [published Online First: 20080305]

For peer review only

For peer review only - http://bmjopen.bmj.com/site/about/guidelines.xhtml

12. Ward LC. Human body composition: yesterday, today, and tomorrow. *Eur J Clin Nutr* 2018;72(9):1201-07. doi: 10.1038/s41430-018-0210-2 [published Online First: 2018/09/07]

13. Adair LS, Fall CH, Osmond C, et al. Associations of Linear Growth and Relative Weight Gain During Early Life With Adult Health and Human Capital in Countries of Low and Middle Income: Findings From Five Birth Cohort Studies. *Lancet (London, England)* 2013;382(9891) doi: 10.1016/S0140-6736(13)60103-8

14. Kuzawa CW, Hallal PC, Adair L, et al. Birth weight, postnatal weight gain, and adult body composition in five low and middle income countries. *Am J Hum Biol* 2012;24(1):5-13. doi: 10.1002/ajhb.21227 [published Online First: 2011/11/28]

15. Weng SF, Redsell SA, Swift JA, et al. Systematic review and meta-analyses of risk factors for childhood overweight identifiable during infancy. *Arch Dis Child* 2012;97(12):1019-26. doi: 10.1136/archdischild-2012-302263 [published Online First: 20121029]

16. Rallis D, Balomenou F, Tzoufi M, et al. A systematic review indicates an association between birth weight and body fat in childhood. *Acta Paediatr* 2021;110(7):2023-39. doi: 10.1111/apa.15834 [published Online First: 20210315]

17. Yang Z, Huffman SL. Nutrition in pregnancy and early childhood and associations with obesity in developing countries. *Matern Child Nutr* 2013;9 Suppl 1(Suppl 1):105-19. doi: 10.1111/mcn.12010 [published Online First: 2012/11/28]

18. Richter LM, Victora CG, Hallal PC, et al. Cohort profile: the consortium of health-orientated research in transitioning societies. *Int J Epidemiol* 2012;41(3):621-6. doi: 10.1093/ije/dyq251 [published Online First: 2011/01/13]

19. Horta BL, Gigante DP, Gonçalves H, et al. Cohort Profile Update: The 1982 Pelotas (Brazil) Birth Cohort Study. *Int J Epidemiol* 2015;44(2):441, 41a-41e. doi: 10.1093/ije/dyv017 [published Online First: 2015/03/04]

20. Victora CG, Barros FC. Cohort profile: the 1982 Pelotas (Brazil) birth cohort study. *Int J Epidemiol* 2006;35(2):237-42. doi: 10.1093/ije/dyi290 [published Online First: 2005/12/24]

21. Victora CG, Hallal PC, Araújo CL, et al. Cohort Profile: The 1993 Pelotas (Brazil) Birth Cohort Study. *International journal of epidemiology* 2008;37(4) doi: 10.1093/ije/dym177

22. Stein AD, Melgar P, Hoddinott J, et al. Cohort Profile: the Institute of Nutrition of Central America and Panama (INCAP) Nutrition Trial Cohort Study. *Int J Epidemiol* 2008;37(4):716-20. doi: 10.1093/ije/dyn028 [published Online First: 2008/02/21]

23. Bhargava SK, Sachdev HS, Fall CH, et al. Relation of serial changes in childhood body-mass index to impaired glucose tolerance in young adulthood. *N Engl J Med* 2004;350(9):865-75.

For peer review only

24. Adair LS, Popkin BM, Akin JS, et al. Cohort profile: the Cebu longitudinal health and nutrition survey. *Int J Epidemiol* 2011;40(3):619-25. doi: 10.1093/ije/dyq085 [published Online First: 2010/05/29]

25. Richter L, Norris S, Pettifor J, et al. Cohort Profile: Mandela's children: the 1990 Birth to Twenty study in South Africa. *Int J Epidemiol* 2007;36(3):504-11. doi: 10.1093/ije/dym016 [published Online First: 2007/03/16]

26. Mei Z, Grummer-Strawn LM. Standard deviation of anthropometric Z-scores as a data quality assessment tool using the 2006 WHO growth standards: a cross country analysis. *Bull World Health Organ* 2007;85(6):441-8. doi: 10.2471/blt.06.034421

27. Jesson J, Schomaker M, Malasteste K, et al. Stunting and growth velocity of adolescents with perinatally acquired HIV: differential evolution for males and females. A multiregional analysis from the IeDEA global paediatric collaboration. *J Int AIDS Soc* 2019;22(11):e25412. doi: 10.1002/jia2.25412

28. Prioreschi A, Munthali RJ, Kagura J, et al. The associations between adult body composition and abdominal adiposity outcomes, and relative weight gain and linear growth from birth to age 22 in the Birth to Twenty Plus cohort, South Africa. *PLoS One* 2018;13(1):e0190483. doi: 10.1371/journal.pone.0190483 [published Online First: 20180116]

29. Gonçalves H, Wehrmeister FC, Assunção MCF, et al. Cohort Profile Update: The 1993 Pelotas (Brazil) Birth Cohort follow-up at 22 years. *Int J Epidemiol* 2018;47(5):1389-90e. doi: 10.1093/ije/dyx249 [published Online First: 2017/12/15]

30. Kroker-Lobos MF, Ramirez-Zea M, Stein AD. Overweight and Obesity, Cardiometabolic Health, and Body Composition: Findings From the Follow-Up Studies of the INCAP Longitudinal Study. *Food and Nutrition Bulletin* 2020;41(1_suppl):S59-S68. doi: 10.1177/0379572120903222

31. Menezes AMB, Oliveira PD, Wehrmeister FC, et al. Associations between growth from birth to 18 years, intelligence, and schooling in a Brazilian cohort. *The American journal of clinical nutrition* 2020 doi: 10.1093/ajcn/nqaa047 [published Online First: 2020/04/03]

32. Li H, Stein AD, Barnhart HX, et al. Associations between prenatal and postnatal growth and adult body size and composition. *The American journal of clinical nutrition* 2003;77(6):1498-505. doi: 10.1093/ajcn/77.6.1498 [published Online First: 2003/06/07]

33. Sachdev HS, Fall CH, Osmond C, et al. Anthropometric indicators of body composition in young adults: relation to size at birth and serial measurements of body mass index in childhood in the New Delhi birth cohort. *The American journal of clinical nutrition* 2005;82(2):456-66. doi: 10.1093/ajcn.82.2.456 [published Online First: 2005/08/10]

34. Adair LS. Size at birth and growth trajectories to young adulthood. *Am J Hum Biol* 2007;19(3):327-37. doi: 10.1002/ajhb.20587

For peer review only

35. WHO. The WHO Child Growth Standards: World Health Organization; 2016 [updated 2016-06-08 09:11:46. Available from: https://www.who.int/childgrowth/en/.

36. Li C, Ford ES, Zhao G, et al. Estimates of body composition with dual-energy X-ray absorptiometry in adults. *The American journal of clinical nutrition* 2009;90(6):1457-65. doi: 10.3945/ajcn.2009.28141 [published Online First: 2009/10/09]

37. Harrer M, Cuijpers P, Furukawa TA, et al. Doing Meta-Analysis With R: A Hands-On Guide.

38. Corvalán C, Gregory CO, Ramirez-Zea M, et al. Size at birth, infant, early and later childhood growth and adult body composition: a prospective study in a stunted population. *Int J Epidemiol* 2007;36(3):550-7. doi: 10.1093/ije/dym010 [published Online First: 2007/03/23]

39. Savvidis C, Tournis S, Dede AD. Obesity and bone metabolism. *Hormones (Athens)* 2018;17(2):205-17. doi: 10.1007/s42000-018-0018-4 [published Online First: 20180424]

40. Tomlinson DJ, Erskine RM, Morse CI, et al. The impact of obesity on skeletal muscle strength and structure through adolescence to old age. *Biogerontology* 2016;17(3):467-83. doi: 10.1007/s10522-015-9626-4 [published Online First: 20151214]

41. Guo SS, Huang C, Maynard LM, et al. Body mass index during childhood, adolescence and young adulthood in relation to adult overweight and adiposity: the Fels Longitudinal Study. *International journal of obesity and related metabolic disorders : journal of the International Association for the Study of Obesity* 2000;24(12):1628-35. doi: 10.1038/sj.ijo.0801461

42. Araújo de França GV, De Lucia Rolfe E, Horta BL, et al. Associations of birth weight, linear growth and relative weight gain throughout life with abdominal fat depots in adulthood: the 1982 Pelotas (Brazil) birth cohort study. *International journal of obesity (2005)* 2016;40(1):14-21. doi: 10.1038/ijo.2015.192 [published Online First: 20150923]

43. Larqué E, Labayen I, Flodmark CE, et al. From conception to infancy - early risk factors for childhood obesity. *Nat Rev Endocrinol* 2019;15(8):456-78. doi: 10.1038/s41574-019-0219-1 [published Online First: 20190703]

44. Mazariegos M, Varghese JS, Kroker-Lobos MF, et al. Age at childbirth and change in BMI across the life-course: evidence from the INCAP Longitudinal Study. *BMC Pregnancy Childbirth* 2022;22(1):151. doi: 10.1186/s12884-022-04485-6 [published Online First: 20220224]

45. Popkin BM, Corvalan C, Grummer-Strawn LM. Dynamics of the double burden of malnutrition and the changing nutrition reality. *Lancet* 2020;395(10217):65-74. doi: 10.1016/s0140-6736(19)32497-3 [published Online First: 20191215]

46. Rivera JA, Martorell R, Ruel MT, et al. Nutritional supplementation during the preschool years influences body size and composition of Guatemalan adolescents. *The Journal of nutrition*

For peer review only

712 1995;125(4 Suppl):1068s-77s. doi: 10.1093/jn/125.suppl_4.1068S [published Online First: 1995/04/01]

Figure Legend

Figure 1. Flowchart of analytic samples selection, COHORTS

For peer review only

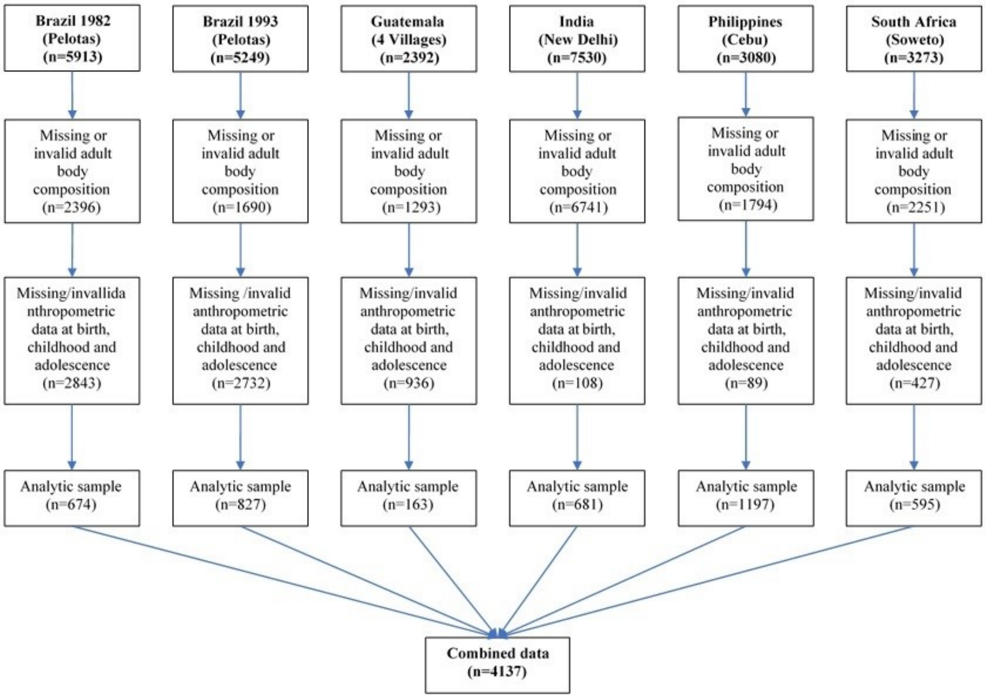

Figure 1. Flowchart of analytic samples selection, COHORTS

72x50mm (300 x 300 DPI)

1

Supplementary Tables

Supplementary Table 1. Data missing patterns (covariates) of analytic samples at each study site

Covariates	Brazil 1982 (n=674)	Brazil 1993 (n=827)	Guatemala (n=163)	India (n=681)	The Philippines (n=1197)	South Africa (n=595)
Sex	0	0	0	0	0	0
Gestational age	137 (20.3%)	91 (11%)	10 (6.1%)	149	8 (0.7%)	4 (0.7%)
Birth order	0	0	0	256 (37.6%)	0	0
Maternal age at first child birth	0	0	0	227 (33.3%)	0	0
Maternal height	11 (1.6%)	8 (1%)	0	246 (36.1%)	0	84 (14.1%)
Maternal schooling	1 (0.1%)	12 (1.4%)	1 (0.6%)	62	0	24 (4.0%)
Maternal marital status	0	0	0	0	0	0
Paternal age at child birth	163 (24.2%)	NA	3 (1.8%)	NA	36 (3.0%)	NA
Paternal schooling	40 (5.9%)	48 (5.8%)	3 (1.8%)	116 (17.0%)	59 (4.9%)	158 (26.5%)
Income in childhood	7 (1.0%)	6 (0.7%)	NA	222 (32.6%)	5 (0.4%)	244 (41.0%)
Wealth index in childhood	0	NA	1 (0.6%)	222 (32.6%)	0	25 (4.2%)
Age at menarche (women)	0	NA	7 (4.3%)	52 (7.6%)	1 (0.1%)	4 (0.7%)
Teenage childbearing	12 (1.8%)	24 (2.9%)	24 (14.7%)	280 (41.1%)	253 (21.1%)	NA
Skin color Brazil	0	0	NA	NA	NA	NA

NA: not available variable

For peer review only

Supplementary Table 2. Characteristics of participants excluded (missing anthropometric and body composition data) and included in the analyses at each study site

	Brazil 82		Brazil 93		Guatemala		India		The Philippines		South Africa	
	Excluded (N=5239)	Analytic sample (N=674)	Excluded (N=4421)	Analytic sample (N=827)	Excluded (N=2229)	Analytic sample (N=163)	Excluded (N=6849)	Analytic sample (N=681)	Excluded (N=1883)	Analytic sample (N=1197)	Excluded (N=2678)	Analytic sample (N=595)
Birth Variables												
Sex												
n	5239	674	4421	827	2229	163	6849	681	1883	1197	2678	595
Women	48.6%	49.0%	49.6%	54.7%*	47.8%	58.9%*	48.9%	37.4%*	48.2%	45.2%	51.3%	51.8%
Gestational age (weeks)												
n	4063	537	3936	736	698	153	847	532	1859	1189	2580	591
Mean (SD)	39.3 (1.9)	39.5 (1.8)	39.5 (2.3)	39.3 (2.5)	39.3 (2.9)	39.2 (3.4)	38.9 (2.7)	38.9 (2.4)	38.7 (2.2)	38.8 (2.1)	38.2 (1.9)	37.9* (2.0)
Birth length (cm)												
n	NA	NA	4335	827	689	163	5964	681	1853	1197	NA	NA
Mean (SD)	NA	NA	48.9 (2.3)	48.1* (2.6)	50.0 (2.3)	49.0*(2.4)	48.2 (2.3)	48.4 (2.1)	49.1 (2.1)	49.1 (2.0)	NA	NA
Birth weight (kg)												
n	5131	674	4405	827	810	163	6128	681	1832	1197	2672	595
Mean (SD)	3.2 (0.6)	3.3* (0.6)	3.2 (0.5)	3.0* (0.6)	3.1 (0.5)	3.0 (0.5)	2.8 (0.4)	2.8 (0.4)	3.0 (0.5)	3.0 (0.4)	3.1 (0.5)	3.1 (0.5)
Childhood variables												
HAZ in infancy												
n	4162	674	536	827	922	163	4661	681	1307	1197	1210	595
Mean (SD)	-0.7 (1.2)	-0.5*(1.2)	-0.3 (1.6)	-0.3 (1.3)	-2.9 (1.2)	-2.9 (1.1)	-1.9 (1.2)	-2.0 (1.1)	-2.4 (1.2)	-2.4 (1.1)	-1.1 (1.2)	-1.2 (1.0)
HAZ in childhood												
n	3978	674	440	827	951	163	4151	681	1064	1197	1209	595
Mean (SD)	-0.7 (1.1)	-0.6 (1.1)	-0.3 (1.3)	-0.3 (1.1)	-2.4 (1.0)	-2.3 (0.9)	-1.9 (1.1)	-2.0 (1.0)	-2.0 (1.0)	-2.1 (0.9)	-0.9 (0.9)	-1.0 (0.9)
HAZ in adolescence												
n	398	674	3172	827	724	163	2384	681	891	1197	1328	595
Mean (SD)	-0.3 (1.0)	-0.2 (1.0)	-0.1 (1.0)	-0.3* (1.0)	-2.0 (0.9)	-1.9 (0.8)	-1.1 (1.0)	-1.2 (1.0)	-1.8 (0.9)	-1.9* (0.8)	-0.6 (1.0)	-0.6 (0.9)
WAZ in infancy												
n	4163	674	534	827	922	163	4623	681	1307	1197	1596	595
Mean (SD)	0.1 (1.1)	0.2 (1.1)	0.2 (1.4)	0.3 (1.1)	-1.7 (1.0)	-1.7(1.0)	-1.4 (1.1)	-1.5 (1.1)	-1.7 (1.1)	-1.7 (0.9)	-0.4 (1.0)	-0.5 (1.0)
WAZ in childhood												
n	3978	674	444	827	950	163	4127	681	1064	1197	1604	595
Mean (SD)	-0.0 (1.1)	0.1 (1.0)	0.5 (2.9)	0.1 (1.2)*	-1.4 (0.8)	-1.4(0.8)	-1.3 (1.0)	-1.4 (0.9)	-1.8 (1.1)	-2.0 (0.9)*	-0.4 (0.9)	-0.5 (0.9)*
BAZ in adolescence												
n	398	674	3172	827	723	163	2384	681	891	1197	1323	595
Mean (SD)	0.3 (1.1)	0.3 (1.1)	0.4 (1.2)	0.3* (1.1)	-0.5 (0.8)	-0.3 (0.9)	-0.8 (1.2)	-1.0* (1.2)	-0.8 (1.1)	-0.9 (1.0)	-0.1 (1.3)	-0.1 (1.2)

For peer review only

Adult variables												
Age (years)												
n	2989	667	2986	827	1115	153	149	681	129	1197	913	484
Mean (SD)	30.2 (0.3)	30.2 (0.3)	22.6 (0.3)	22.6 (0.3)	47.7 (4.4)	45.5*(1.1)	46.1 (1.2)	46.1 (1.1)	34.5 (0.5)	34.4 (0.5)	22.0 (0.4)	22.0 (0.4)
Height (cm)												
n	2933	674	2765	827	982	163	140	681	129	1197	921	595
Mean (SD)	167.5(9.2)	168.6*(9.3)	167.8(9.4)	166.4*(10.0)	156.3(8.3)	157.3(8.2)	164.7(9.1)	164.1(9.8)	152.6(26.1)	157.4*(8.1)	165.7(8.9)	165.4(8.6)
Weight (kg)												
n	2877	674	2764	827	982	163	136	681	126	1197	911	595
Mean (SD)	75.6(17.7)	76.6(17.6)	71.9(16.8)	69.6*(16.4)	68.7(13.2)	70.2(12.9)	76.5(14.5)	76.6(15.0)	62.1(14.9)	62.1 (13.1)	64.8(14.8)	63.8(13.3)
BMI (kg/m^2)												
n	2863	671	2735	827	982	163	135	681	107	1197	911	595
Mean (SD)	26.8 (5.5)	26.9 (5.5)	25.3 (5.3)	25.0 (5.4)	28.1 (5.1)	28.5 (5.1)	28.4 (5.5)	28.4 (4.8)	24.8 (5.0)	25.0 (4.6)	23.7 (5.7)	23.4 (5.1)
Waist circumference (cm)												
n	2893	674	2766	827	982	163	138	681	103	1197	902	595
Mean (SD)	84.7(12.6)	85.4(12.5)	80.2(11.6)	79.2 (11.5)	98.7(12.4)	99.1(11.6)	97.8(12.1)	97.3(12.2)	81.1(12.6)	81.5(11.1)	78.4(11.8)	79.2(11.8)
FMI, kg/m^2												
n	2849	674	2735	827	939	163	112	681	90	1197	452	595
Mean (SD)	8.7 (4.6)	8.8 (4.5)	7.7 (4.5)	7.5 (4.6)	10.7 (4.1)	10.7(3.9)	8.6 (3.7)	9.1 (3.6)	6.7 (3.4)	7.1 (3.5)	6.7 (4.3)	6.9 (4.2)
FFMI, kg/m^2												
n	2849	674	2735	827	941	163	112	681	90	1197	452	595
Mean (SD)	18.1 (2.7)	18.1 (2.7)	17.5 (2.6)	17.3 (2.5)	17.5 (2.4)	17.8 (2.5)	19.5 (2.6)	19.3 (2.4)	17.6 (2.2)	17.9 (2.3)	14.8 (2.1)	14.6 (2.0)

*p<0.01 based on Bonferroni-adjusted comparisons of participants excluded and included in the analytic sample at each study site

For peer review only

Supplementary Table 3. Adjusted associations between weight at birth, conditional growth (height and relative weight) in infancy, childhood, and adolescence with adult fat mass index (FMI, SD units), stratified by study site and sex[1]

	Brazil 1982		Brazil 1993		Guatemala		India		The Philippines		South Africa	
	Men (n = 344)	Women (n = 330)	Men (n = 375)	Women (n = 452)	Men (n = 67)	Women (n = 96)	Men (n = 426)	Women (n = 255)	Men (n = 656)	Women (n = 541)	Men (n = 287)	Women (n = 308)
**Conditional growth, z-scores[2]**												
Birth weight	0.04 (-0.03,0.11)	-0.02 (-0.11,0.06)	0.02 (-0.05,0.09)	0.10* (0.04,0.17)	0.11 (-0.18,0.39)	0.2 (-0.04,0.44)	0.15* (0.05,0.25)	0.19* (0.05,0.33)	0.10* (0.03,0.18)	0.12* (0.04,0.2)	-0.02 (-0.13,0.08)	0.02 (-0.07,0.11)
Conditional relative weight in infancy	0.18* (0.08,0.28)	0.31* (0.17,0.45)	0.22* (0.12,0.32)	0.23* (0.14,0.32)	0.49 (-0.08,1.06)	-0.06 (-0.53,0.40)	0.22* (0.08,0.35)	0.26* (0.07,0.45)	0.25* (0.15,0.36)	0.33* (0.2,0.45)	0.22* (0.12,0.32)	0.24* (0.12,0.36)
Conditional relative weight in childhood	0.39* (0.24,0.54)	0.48* (0.29,0.68)	0.32* (0.22,0.42)	0.50* (0.4,0.61)	0.03 (-0.82,0.88)	0.43 (-0.19,1.05)	0.34* (0.13,0.55)	0.03 (-0.22,0.29)	0.58* (0.46,0.71)	0.72* (0.57,0.88)	0.28* (0.11,0.44)	0.29* (0.11,0.46)
Conditional relative weight in adolescence	0.43* (0.35,0.51)	0.62* (0.52,0.72)	0.38* (0.30,0.45)	0.56* (0.45,0.66)	0.20 (-0.31,0.72)	0.52* (0.12,0.92)	0.28* (0.19,0.37)	0.49* (0.34,0.65)	0.38* (0.29,0.47)	0.42* (0.32,0.52)	0.57* (0.47,0.66)	0.58* (0.49,0.67)
Conditional height in infancy	0.01 (-0.06,0.09)	0.17* (0.07,0.27)	0.05* (0,0.11)	0.06 (-0.01,0.13)	0.27 (-0.06,0.59)	0.09 (-0.2,0.38)	0.10* (0.00,0.2)	0.11 (-0.04,0.26)	0.12* (0.05,0.19)	0.14* (0.06,0.21)	0.07 (-0.03,0.16)	0.10* (0,0.19)
Conditional height in childhood	-0.07 (-0.2,0.07)	0.00 (-0.17,0.17)	0.13* (0.05,0.21)	0.14* (0.03,0.24)	-0.11 (-0.58,0.35)	0.01 (-0.62,0.64)	0.11 (-0.07,0.28)	0.10 (-0.14,0.34)	0.13* (0.02,0.24)	0.25* (0.13,0.37)	0.10 (-0.07,0.27)	0.17* (0.01,0.34)
Conditional height in adolescence	0.07 (-0.04,0.17)	0.22* (0.08,0.37)	0.08 (-0.01,0.16)	0.03 (-0.09,0.15)	0.37 (-0.11,0.85)	-0.07 (-0.66,0.52)	0.00 (-0.12,0.12)	0.05 (-0.23,0.33)	0.00 (-0.15,0.14)	-0.10 (-0.27,0.08)	-0.10 (-0.25,0.05)	-0.07 (-0.22,0.08)

[1] Values are standardized linear regression coefficients (βs and 95% CIs). In adjusted analyses we controlled for birth characteristics (gestational age, birth order), maternal characteristics (height, age at childbirth, schooling, marital status), paternal characteristics (age, schooling), income and/or wealth index of child's household at birth, and age in adulthood. In women' models, we adjusted for age of menarche and teenage childbearing. Additionally, we controlled for maternal skin color in both Brazilian cohorts and adjusted for birth year and intervention group (village fixed effects) in Guatemala analysis. * P value < 0.05; SD = Standard Deviation

[2] We used birth weight as the anchor in all conditional models given that this measure was available across all study sites

For peer review only

Supplementary Table 4. Adjusted associations between weight at birth, conditional growth (height and relative weight) in infancy, childhood, and adolescence with adult fat-free mass index (FFMI, SD units), stratified by study site and sex[1]

	Brazil 1982		Brazil 1993		Guatemala		India		The Philippines		South Africa	
	Men	Women	Men	Women	Men	Women	Men	Women	Men	Women	Men	Women
	(n = 344)	(n = 330)	(n = 375)	(n = 452)	(n = 67)	(n = 96)	(n = 426)	(n = 255)	(n = 656)	(n = 541)	(n = 287)	(n = 308)
**Conditional growth, z-scores[2]**												
Birth weight	0.10* (0.04,0.17)	0.06* (0.01,0.12)	0.05* (0.00,0.10)	0.10* (0.05,0.14)	-0.06 (-0.31,0.19)	0.41* (0.2,0.62)	0.11* (0.01,0.20)	0.12 (-0.01,0.25)	0.13* (0.06,0.20)	0.11* (0.03,0.18)	0.20* (0.1,0.3)	0.14* (0.06,0.23)
Conditional relative weight in infancy	0.30* (0.20,0.39)	0.20* (0.10,0.29)	0.30* (0.22,0.37)	0.29* (0.22,0.36)	0.46 (-0.05,0.96)	0.23 (-0.18,0.64)	0.34* (0.22,0.47)	0.40* (0.23,0.57)	0.31* (0.21,0.41)	0.38* (0.26,0.49)	0.19* (0.09,0.28)	0.21* (0.1,0.33)
Conditional relative weight in childhood	0.41* (0.27,0.55)	0.39* (0.27,0.52)	0.43* (0.33,0.52)	0.41* (0.33,0.49)	0.35 (-0.40,1.10)	0.20 (-0.35,0.74)	0.44* (0.25,0.64)	0.37* (0.13,0.61)	0.71* (0.59,0.83)	0.71* (0.56,0.86)	0.31* (0.15,0.46)	0.40* (0.24,0.57)
Conditional relative weight in adolescence	0.39* (0.31,0.46)	0.39* (0.33,0.45)	0.35* (0.28,0.41)	0.38* (0.32,0.44)	0.58* (0.13,1.03)	0.44* (0.09,0.80)	0.36* (0.28,0.45)	0.52* (0.38,0.66)	0.47* (0.39,0.55)	0.47* (0.38,0.57)	0.61* (0.52,0.69)	0.62* (0.53,0.71)
Conditional height in infancy	0.05 (-0.02,0.12)	0.06 (-0.01,0.12)	-0.01 (-0.07,0.05)	0.06* (0.02,0.1)	0.34* (0.05,0.63)	0.15 (-0.1,0.41)	0.13* (0.04,0.22)	-0.05 (-0.19,0.09)	0.14* (0.07,0.20)	0.17* (0.09,0.24)	0.05 (-0.04,0.14)	0.19* (0.11,0.28)
Conditional height in childhood	-0.08 (-0.2,0.04)	-0.04 (-0.15,0.07)	0.03 (-0.06,0.12)	0.06 (0,0.12)	-0.12 (-0.53,0.29)	-0.4 (-0.95,0.15)	0.10 (-0.06,0.27)	-0.14 (-0.36,0.08)	0.16* (0.06,0.27)	0.24* (0.13,0.35)	-0.03 (-0.19,0.13)	0.07 (-0.09,0.23)
Conditional height in adolescence	0.02 (-0.08,0.11)	0.00 (-0.1,0.09)	-0.08 (-0.18,0.02)	0.08 (-0.01,0.17)	0.24 (-0.19,0.66)	-0.07 (-0.59,0.45)	0.07 (-0.04,0.18)	-0.10 (-0.36,0.16)	-0.04 (-0.17,0.1)	-0.11 (-0.28,0.06)	-0.02 (-0.16,0.12)	0.12 (-0.02,0.27)

[1] Values are standardized linear regression coefficients (βs and 95% CIs). In adjusted analyses we controlled for birth characteristics (gestational age, birth order), maternal characteristics (height, age at childbirth, schooling, marital status), paternal characteristics (age, schooling), income and/or wealth index of child's household at birth, and age in adulthood. In women' models, we adjusted for age of menarche and teenage childbearing. Additionally, we controlled for maternal skin color in both Brazilian cohorts and adjusted for birth year and intervention group (village fixed effects) in Guatemala analysis. * P value < 0.05; SD = Standard Deviation

[2] We used birth weight as the anchor in all conditional models given that this measure was available across all study sites

For peer review only

Supplementary Table 5. Adjusted associations between weight at birth, conditional growth (height and relative weight) in infancy, childhood, and adolescence with adult fat mass/fat free mass ratio (FM/FFM, SD units), stratified by study site and sex[1]

	Brazil 1982		Brazil 1993		Guatemala		India		The Philippines		South Africa	
	Men	Women	Men	Women	Men	Women	Men	Women	Men	Women	Men	Women
	(n = 344)	(n = 330)	(n = 375)	(n = 452)	(n = 67)	(n = 96)	(n = 426)	(n = 255)	(n = 656)	(n = 541)	(n = 287)	(n = 308)
**Conditional growth, z-scores**[2]												
Birth weight	0.01 (-0.05,0.08)	-0.05 (-0.14,0.03)	0.01 (-0.04,0.07)	0.07* (0.01,0.13)	0.18 (-0.11,0.48)	0.02 (-0.24,0.27)	0.16* (0.05,0.26)	0.20* (0.06,0.34)	0.08* (0.01,0.16)	0.12* (0.04,0.2)	-0.08 (-0.19,0.03)	-0.03 (-0.13,0.07)
Conditional relative weight in infancy	0.11* (0.02,0.20)	0.24* (0.1,0.38)	0.15* (0.06,0.24)	0.14* (0.05,0.24)	0.37 (-0.23,0.96)	-0.25 (-0.75,0.24)	0.15* (0.01,0.29)	0.16 (-0.03,0.36)	0.21* (0.1,0.32)	0.32* (0.19,0.44)	0.20* (0.09,0.31)	0.21* (0.08,0.34)
Conditional relative weight in childhood	0.26* (0.13,0.40)	0.38* (0.18,0.57)	0.22* (0.13,0.3)	0.39* (0.28,0.49)	-0.16 (-1.04,0.73)	0.31 (-0.35,0.97)	0.24* (0.02,0.46)	-0.12 (-0.39,0.14)	0.51* (0.38,0.65)	0.70* (0.54,0.85)	0.24* (0.06,0.42)	0.20* (0,0.39)
Conditional relative weight in adolescence	0.30* (0.23,0.38)	0.50* (0.4,0.59)	0.30* (0.23,0.37)	0.45* (0.36,0.54)	0.00 (-0.53,0.54)	0.31 (-0.12,0.74)	0.20* (0.11,0.30)	0.39* (0.24,0.55)	0.34* (0.25,0.43)	0.40* (0.30,0.51)	0.49* (0.39,0.6)	0.46* (0.36,0.56)
Conditional height in infancy	0.00 (-0.07,0.07)	0.16* (0.06,0.26)	0.05 (0,0.09)	0.04 (-0.03,0.10)	0.16 (-0.17,0.5)	-0.03 (-0.34,0.28)	0.07 (-0.03,0.17)	0.15 (-0.01,0.31)	0.11* (0.04,0.19)	0.13* (0.05,0.21)	0.06 (-0.04,0.16)	0.04 (-0.06,0.15)
Conditional height in childhood	-0.03 (-0.15,0.09)	0.04 (-0.13,0.20)	0.12* (0.05,0.19)	0.13* (0.04,0.23)	-0.08 (-0.56,0.41)	0.26 (-0.41,0.92)	0.11 (-0.07,0.29)	0.16 (-0.08,0.41)	0.13* (0.02,0.25)	0.24* (0.13,0.36)	0.10 (-0.09,0.28)	0.17 (-0.01,0.36)
Conditional height in adolescence	0.05 (-0.05,0.14)	0.26* (0.12,0.41)	0.08* (0.01,0.16)	-0.01 (-0.14,0.12)	0.41 (-0.09,0.91)	-0.02 (-0.65,0.60)	-0.01 (-0.14,0.11)	0.09 (-0.2,0.37)	0.02 (-0.13,0.17)	-0.11 (-0.29,0.07)	-0.12 (-0.28,0.04)	-0.17* (-0.34,0)

[1] Values are standardized linear regression coefficients (βs and 95% CIs). In adjusted analyses we controlled for birth characteristics (gestational age, birth order), maternal characteristics (height, age at childbirth, schooling, marital status), paternal characteristics (age, schooling), income and/or wealth index of child's household at birth, and age in adulthood. In women' models, we adjusted for age of menarche and teenage childbearing. Additionally, we controlled for maternal skin color in both Brazilian cohorts and adjusted for birth year and intervention group (village fixed effects) in Guatemala analysis. * P value < 0.05; SD = Standard Deviation

[2] We used birth weight as the anchor in all conditional models given that this measure was available across all study sites

For peer review only

Supplementary Table 6. Adjusted associations between weight at birth, conditional growth (height and relative weight) in infancy, childhood, and adolescence with adult body mass index (BMI, SD units), stratified by study site and sex[1]

	Brazil 1982		Brazil 1993		Guatemala		India		The Philippines		South Africa	
	Men (n = 344)	Women (n = 330)	Men (n = 375)	Women (n = 452)	Men (n = 67)	Women (n = 96)	Men (n = 426)	Women (n = 255)	Men (n = 656)	Women (n = 541)	Men (n = 287)	Women (n = 308)
**Conditional growth, z-scores[2]**												
Birth weight	0.08* (0.01,0.15)	0.01 (-0.07,0.1)	0.04 (-0.02,0.1)	0.13* (0.06,0.20)	0.03 (-0.24,0.3)	0.33* (0.11,0.55)	0.13* (0.04,0.23)	0.18* (0.04,0.31)	0.12* (0.04,0.19)	0.12* (0.04,0.20)	0.11* (0.02,0.19)	0.07 (-0.02,0.15)
Conditional relative weight in infancy	0.29* (0.20,0.39)	0.35* (0.21,0.49)	0.33* (0.23,0.42)	0.33* (0.24,0.42)	0.52 (-0.01,1.06)	0.07 (-0.36,0.5)	0.30* (0.17,0.42)	0.33* (0.15,0.51)	0.28* (0.18,0.38)	0.31* (0.19,0.43)	0.22* (0.14,0.31)	0.24* (0.13,0.35)
Conditional relative weight in childhood	0.53* (0.38,0.67)	0.59* (0.39,0.79)	0.47* (0.38,0.57)	0.62* (0.52,0.73)	0.21 (-0.59,1.01)	0.38 (-0.19,0.96)	0.42* (0.23,0.62)	0.16 (-0.09,0.41)	0.65* (0.53,0.78)	0.70* (0.55,0.85)	0.35* (0.21,0.49)	0.36* (0.2,0.53)
Conditional relative weight in adolescence	0.54* (0.46,0.62)	0.70* (0.6,0.8)	0.49* (0.42,0.55)	0.66* (0.55,0.77)	0.43 (-0.06,0.91)	0.56* (0.18,0.93)	0.35* (0.26,0.44)	0.53* (0.38,0.68)	0.43* (0.34,0.52)	0.44* (0.34,0.54)	0.67* (0.59,0.75)	0.63* (0.55,0.72)
Conditional height in infancy	0.03 (-0.04,0.11)	0.17* (0.07,0.27)	0.04 (-0.01,0.1)	0.08* (0.01,0.15)	0.33* (0.03,0.64)	0.13 (-0.14,0.4)	0.12* (0.03,0.21)	0.06 (-0.09,0.21)	0.13* (0.06,0.2)	0.14* (0.06,0.21)	0.06 (-0.02,0.14)	0.13* (0.04,0.21)
Conditional height in childhood	-0.09 (-0.22,0.04)	-0.02 (-0.19,0.15)	0.13* (0.05,0.21)	0.14* (0.04,0.25)	-0.13 (-0.57,0.31)	-0.18 (-0.76,0.4)	0.10 (-0.06,0.27)	0.03 (-0.21,0.26)	0.15* (0.04,0.26)	0.27* (0.15,0.39)	0.04 (-0.1,0.19)	0.15 (-0.01,0.3)
Conditional height in adolescence	0.06 (-0.04,0.16)	0.18* (0.03,0.33)	0.03 (-0.06,0.12)	0.07 (-0.06,0.19)	0.34 (-0.11,0.79)	-0.08 (-0.63,0.46)	0.03 (-0.08,0.14)	0.01 (-0.26,0.28)	-0.03 (-0.17,0.11)	-0.09 (-0.27,0.08)	-0.08 (-0.21,0.04)	-0.03 (-0.17,0.11)

[1] Values are standardized linear regression coefficients (βs and 95% CIs). In adjusted analyses we controlled for birth characteristics (gestational age, birth order), maternal characteristics (height, age at childbirth, schooling, marital status), paternal characteristics (age, schooling), income and/or wealth index of child's household at birth, and age in adulthood. In women' models, we adjusted for age of menarche and teenage childbearing. Additionally, we controlled for maternal skin color in both Brazilian cohorts and adjusted for birth year and intervention group (village fixed effects) in Guatemala analysis. * P value < 0.05; SD = Standard Deviation

[2] We used birth weight as the anchor in all conditional models given that this measure was available across all study sites

For peer review only

Supplementary Table 7. Adjusted associations between weight at birth, conditional growth (height and relative weight) in infancy, childhood, and adolescence with adult waist circumference (SD units), stratified by study site and sex[1]

	Brazil 1982		Brazil 1993		Guatemala		India		The Philippines		South Africa	
	Men (n = 344)	Women (n = 330)	Men (n = 375)	Women (n = 452)	Men (n = 67)	Women (n = 96)	Men (n = 426)	Women (n = 255)	Men (n = 656)	Women (n = 541)	Men (n = 287)	Women (n = 308)
**Conditional growth, z-scores[2]**												
Birth weight	0.06 (-0.03,0.15)	0.03 (-0.07,0.12)	0.03 (-0.04,0.1)	0.11* (0.01,0.21)	0.03 (-0.26,0.33)	0.30* (0.04,0.57)	0.13* (0.03,0.24)	0.14 (-0.02,0.3)	0.10* (0.01,0.18)	0.08 (-0.02,0.18)	0.03 (-0.08,0.14)	0.01 (-0.11,0.12)
Conditional relative weight in infancy	0.26* (0.14,0.37)	0.26* (0.13,0.39)	0.33* (0.22,0.43)	0.30* (0.21,0.39)	0.53 (-0.04,1.1)	0.12 (-0.32,0.57)	0.24* (0.11,0.37)	0.31* (0.13,0.5)	0.25* (0.15,0.35)	0.25* (0.12,0.37)	0.17* (0.07,0.27)	0.17* (0.04,0.3)
Conditional relative weight in childhood	0.49* (0.32,0.65)	0.49* (0.32,0.67)	0.45* (0.33,0.57)	0.50* (0.38,0.62)	0.32 (-0.5,1.14)	0.17 (-0.41,0.74)	0.44* (0.23,0.64)	0.00 (-0.26,0.26)	0.46* (0.33,0.6)	0.57* (0.41,0.73)	0.28* (0.10,0.45)	0.23* (0.04,0.42)
Conditional relative weight in adolescence	0.53* (0.44,0.62)	0.53* (0.44,0.61)	0.45* (0.37,0.53)	0.58* (0.48,0.68)	0.39 (-0.15,0.92)	0.49* (0.12,0.87)	0.26* (0.16,0.35)	0.42* (0.26,0.59)	0.32* (0.23,0.42)	0.40* (0.3,0.51)	0.51* (0.41,0.61)	0.51* (0.41,0.6)
Conditional height in infancy	0.04 (-0.06,0.15)	0.22* (0.07,0.36)	0.08* (0,0.16)	0.08 (-0.05,0.21)	0.27 (-0.08,0.62)	-0.09 (-0.6,0.41)	0.11 (0,0.22)	-0.18 (-0.43,0.06)	0.22* (0.12,0.32)	0.11 (-0.06,0.27)	0.02 (-0.10,0.14)	0.09 (-0.07,0.25)
Conditional height in childhood	-0.06 (-0.22,0.1)	0.1 (-0.11,0.31)	0.19* (0.08,0.3)	0.19 (-0.01,0.38)	-0.19 (-0.66,0.28)	-0.22 (-0.86,0.42)	0.11 (-0.07,0.3)	0.07 (-0.27,0.41)	0.24* (0.09,0.38)	0.20 (-0.03,0.43)	0.01 (-0.20,0.22)	0.15 (-0.1,0.41)
Conditional height in adolescence	0.06 (-0.06,0.19)	0.26* (0.03,0.49)	0.10 (0,0.21)	0.07 (-0.16,0.31)	0.27 (-0.34,0.88)	-0.42 (-1.42,0.58)	0.06 (-0.06,0.19)	-0.21 (-0.65,0.23)	0.13 (-0.05,0.31)	-0.13 (-0.49,0.22)	-0.07 (-0.25,0.12)	-0.17 (-0.44,0.11)

[1] Values are standardized linear regression coefficients (βs and 95% CIs). In adjusted analyses we controlled for birth characteristics (gestational age, birth order), maternal characteristics (height, age at childbirth, schooling, marital status), paternal characteristics (age, schooling), income and/or wealth index of child's household at birth, and age and height in adulthood. In women' models, we adjusted for age of menarche and teenage childbearing. Additionally, we controlled for maternal skin color in both Brazilian cohorts and adjusted for birth year and intervention group (village fixed effects) in Guatemala analysis. * P value < 0.05; SD = Standard Deviation

[2] We used birth weight as the anchor in all conditional models given that this measure was available across all study sites

For peer review only

Supplementary Table 8. Pooled adjusted associations between length at birth, conditional growth (height and relative weight) in infancy, childhood, and adolescence with adult fat mass and fat-free mass indices, stratified by sex[1]

	FMI (SD units)						FFMI (SD units)					
	Men (n = 1524)			Women (n = 1344)			Men (n = 1524)			Women (n = 1344)		
	β (95%CI)	I^2 statistic (%)[3]	Cochran's Q test (P value)	β (95%CI)	I^2 statistic (%)	Cochran's Q test (P value)	β (95%CI)	I^2 statistic (%)	Cochran's Q test (P value)	β (95%CI)	I^2 statistic (%)	Cochran's Q test (P value)
**Conditional growth, z-scores[2]**												
Birth length	0.06 (-0.03,0.15)	47.4	0.152	0.10 (-0.01,0.21)	53.3	0.129	0.06 (-0.02,0.14)	51.4	0.108	0.07* (0,0.13)	6.9	0.339
Conditional height in infancy	0.11* (0.04,0.19)	0.1	0.417	0.10* (0.03,0.18)	21.4	0.479	0.12 (-0.06,0.29)	76.1	0.004	0.1 (-0.07,0.27)	79.3	0.006
Conditional height in childhood	0.12* (0.04,0.19)	0	0.759	0.16* (0.03,0.3)	24.6	0.375	0.10 (-0.04,0.25)	39.5	0.184	0.06 (-0.33,0.45)	88.4	0.001
Conditional height in adolescence	0.03 (-0.09,0.15)	0	0.415	-0.03 (-0.14,0.07)	0	0.778	0.00 (-0.17,0.16)	43.4	0.136	-0.04 (-0.21,0.13)	42.8	0.207
Conditional relative weight at birth	0.10* (0.05,0.14)	0	0.881	0.11* (0.03,0.19)	0	0.659	0.10* (0.02,0.18)	0.1	0.565	0.14 (-0.03,0.3)	30.7	0.086
Conditional relative weight in infancy	0.24* (0.18,0.3)	0	0.839	0.25* (0.11,0.39)	7.2	0.297	0.32* (0.26,0.38)	0	0.826	0.34* (0.24,0.44)	10.8	0.486
Conditional relative weight in childhood	0.43* (0.16,0.69)	71.4	0.010	0.50 (-0.03,1.03)	90.5	<0.001	0.55* (0.29,0.8)	76.2	0.003	0.50* (0.19,0.82)	77.0	0.004
Conditional relative weight in adolescence	0.34* (0.24,0.44)	32.7	0.267	0.49* (0.37,0.6)	36	0.281	0.41* (0.29,0.54)	55.3	0.093	0.45* (0.34,0.56)	43.2	0.206

[1] Values are standardized linear regression coefficients (*β*s and 95% CIs). FMI, fat mass index; FFMI, fat-free mass index. In adjusted analyses we controlled for birth characteristics (gestational age, birth order), maternal characteristics (height, age at childbirth, schooling, marital status), paternal schooling, income and/or wealth index of child's household at birth, and age in adulthood. In women' models, we adjusted for age of menarche and teenage childbearing. Additionally, we controlled for maternal skin color in Brazil and adjusted for birth year and intervention group (village fixed effects) in Guatemala analysis. * P value < 0.05

[2] We used birth length as the anchor in conditional models of sites that collected this measure (Brazil 1993, Guatemala, India, The Philippines)

[3] As a rule of thumb I^2 = 25% is interpreted as low heterogeneity, 50% moderate heterogeneity, and 75% substantial heterogeneity

For peer review only

Supplementary Table 9. Pooled adjusted associations between length at birth, conditional growth (height and relative weight) in infancy, childhood, and adolescence with adult body mass index and waist circumference, stratified by sex[1]

	BMI (SD units)						Waist circumference (SD units)					
	Men (n = 1524)			Women (n = 1344)			Men (n = 1524)			Women (n = 1344)		
	β (95%CI)	I^2 statistic (%)[3]	Cochran's Q test (P value)	β (95%CI)	I^2 statistic (%)	Cochran's Q test (P value)	β (95%CI)	I^2 statistic (%)	Cochran's Q test (P value)	β (95%CI)	I^2 statistic (%)	Cochran's Q test (P value)
**Conditional growth, z-scores[2]**												
Birth length	0.06 (-0.03,0.15)	53.6	0.096	0.11* (0.03,0.18)	7.1	0.358	0.07 (-0.03,0.16)	43.6	0.168	0.06 (-0.01,0.12)	0	0.744
Conditional height in infancy	0.12* (0.02,0.22)	27.3	0.167	0.11* (0.03,0.18)	7.6	0.482	0.17* (0.06,0.28)	24.8	0.295	0.04 (-0.18,0.26)	46.6	0.176
Conditional height in childhood	0.12* (0.04,0.21)	0.1	0.648	0.15 (-0.08,0.38)	58.2	0.084	0.17* (0.02,0.33)	0.1	0.297	0.14 (-0.02,0.31)	0	0.658
Conditional height in adolescence	0.02 (-0.09,0.12)	0.1	0.512	-0.02 (-0.16,0.11)	8.2	0.599	0.11* (0.04,0.18)	0	0.871	-0.09 (-0.36,0.17)	2.7	0.543
Conditional relative weight at birth	0.10* (0.07,0.14)	0	0.945	0.13* (0.01,0.25)	0	0.288	0.08* (0.02,0.14)	0	0.794	0.13 (-0.01,0.27)	0	0.196
Conditional relative weight in infancy	0.30* (0.25,0.36)	0	0.838	0.30* (0.22,0.38)	0	0.688	0.27* (0.19,0.36)	0	0.653	0.26* (0.19,0.34)	0	0.752
Conditional relative weight in childhood	0.53* (0.32,0.73)	56.3	0.091	0.55* (0.1,0.99)	86.6	0.001	0.45* (0.41,0.48)	0	0.985	0.41 (-0.06,0.88)	86.7	0.001
Conditional relative weight in adolescence	0.42* (0.31,0.53)	56.5	0.100	0.54* (0.37,0.71)	64.5	0.028	0.34* (0.19,0.49)	69.1	0.022	0.47* (0.32,0.62)	53.9	0.095

[1] Values are standardized linear regression coefficients (*β*s and 95% CIs). BMI, body mass index. In adjusted analyses we controlled for birth characteristics (gestational age, birth order), maternal characteristics (height, age at childbirth, schooling, marital status), paternal schooling, income and/or wealth index of child's household at birth, and age in adulthood. In women' models, we adjusted for age of menarche and teenage childbearing. Additionally, we controlled for maternal skin color in Brazil and adjusted for birth year and intervention group (village fixed effects) in Guatemala analysis. * P value < 0.05

[2] We used birth length as the anchor in conditional models of sites that collected this measure (Brazil 1993, Guatemala, India, The Philippines)

[3] As a rule of thumb I^2 = 25% is interpreted as low heterogeneity, 50% moderate heterogeneity, and 75% substantial heterogeneity

For peer review only

Supplementary Table 10. Adjusted associations between length at birth, conditional growth (height and relative weight) in infancy, childhood, and adolescence with adult fat mass index (FMI, SD units), stratified by study site and sex[1]

	Brazil 1982		Brazil 1993		Guatemala		India		The Philippines		South Africa	
	Men (n = 344)	Women (n = 330)	Men (n = 375)	Women (n = 452)	Men (n = 67)	Women (n = 96)	Men (n = 426)	Women (n = 255)	Men (n = 656)	Women (n = 541)	Men (n = 287)	Women (n = 308)
**Conditional growth, z-scores[2]**												
Birth length	NA	NA	0.00 (-0.06,0.06)	0.07* (0.01,0.14)	0.00 (-0.23,0.23)	0.06 (-0.16,0.28)	0.11* (0.02,0.2)	0.22* (0.10,0.33)	0.07* (0.01,0.14)	0.07 (0,0.14)	NA	NA
Conditional height in infancy	NA	NA	0.07* (0.02,0.13)	0.07 (0,0.13)	0.30 (-0.04,0.64)	0.11 (-0.21,0.42)	0.10* (0.00,0.20)	0.08 (-0.07,0.23)	0.13* (0.06,0.2)	0.15* (0.07,0.22)	NA	NA
Conditional height in childhood	NA	NA	0.13* (0.05,0.21)	0.14* (0.03,0.24)	-0.11 (-0.58,0.35)	0.03 (-0.61,0.66)	0.11 (-0.07,0.28)	0.07 (-0.16,0.31)	0.14* (0.02,0.25)	0.25* (0.13,0.37)	NA	NA
Conditional height in adolescence	NA	NA	0.07 (-0.01,0.16)	0.03 (-0.1,0.15)	0.35 (-0.14,0.84)	-0.06 (-0.66,0.54)	0.00 (-0.12,0.12)	0.00 (-0.28,0.28)	0.00 (-0.15,0.14)	-0.09 (-0.27,0.09)	NA	NA
Conditional relative weight at birth	NA	NA	0.07 (-0.03,0.17)	0.11* (0.01,0.21)	0.14 (-0.18,0.47)	0.23 (-0.05,0.51)	0.13 (0.00,0.27)	0.02 (-0.17,0.21)	0.08 (-0.02,0.17)	0.12* (0.02,0.22)	NA	NA
Conditional relative weight in infancy	NA	NA	0.21* (0.11,0.31)	0.22* (0.13,0.31)	0.44 (-0.15,1.03)	-0.07 (-0.54,0.4)	0.22* (0.08,0.35)	0.27* (0.08,0.45)	0.25* (0.15,0.36)	0.32* (0.2,0.45)	NA	NA
Conditional relative weight in childhood	NA	NA	0.32* (0.22,0.42)	0.51* (0.41,0.62)	0.05 (-0.81,0.91)	0.44 (-0.18,1.07)	0.34* (0.13,0.55)	0.00 (-0.26,0.26)	0.59* (0.46,0.71)	0.72* (0.57,0.88)	NA	NA
Conditional relative weight in adolescence	NA	NA	0.38* (0.3,0.45)	0.56* (0.46,0.67)	0.18 (-0.35,0.7)	0.52* (0.11,0.93)	0.28* (0.19,0.37)	0.49* (0.34,0.64)	0.38* (0.29,0.47)	0.42* (0.32,0.52)	NA	NA

[1] Values are standardized linear regression coefficients (βs and 95% CIs). In adjusted analyses we controlled for birth characteristics (gestational age, birth order), maternal characteristics (height, age at childbirth, schooling, marital status), paternal characteristics (age, schooling), income and/or wealth index of child's household at birth, and age in adulthood. In women' models, we adjusted for age of menarche and teenage childbearing. Additionally, we controlled for maternal skin color in Brazil and adjusted for birth year and intervention group (village fixed effects) in Guatemala analysis. * P value < 0.05; SD = Standard Deviation

[2] We used birth length as the anchor in conditional models of sites that collected this measure (Brazil 1993, Guatemala, India, The Philippines)

For peer review only

Supplementary Table 11. Adjusted associations between length at birth, conditional growth (height and relative weight) in infancy, childhood, and adolescence with adult fat-free mass index (FFMI, SD units), stratified by study site and sex[1]

	Brazil 1982		Brazil 1993		Guatemala		India		The Philippines		South Africa	
	Men (n = 344)	Women (n = 330)	Men (n = 375)	Women (n = 452)	Men (n = 67)	Women (n = 96)	Men (n = 426)	Women (n = 255)	Men (n = 656)	Women (n = 541)	Men (n = 287)	Women (n = 308)
**Conditional growth, z-scores[2]**												
Birth length	NA	NA	0.00 (-0.05,0.05)	0.08* (0.04,0.12)	0.00 (-0.21,0.2)	0.18 (-0.01,0.38)	0.08 (-0.01,0.17)	0.09 (-0.02,0.19)	0.09* (0.03,0.15)	0.03 (-0.04,0.09)	NA	NA
Conditional height in infancy	NA	NA	0.01 (-0.06,0.07)	0.07* (0.02,0.11)	0.34* (0.04,0.64)	0.16 (-0.11,0.43)	0.13* (0.04,0.23)	-0.05 (-0.2,0.09)	0.14* (0.08,0.21)	0.19* (0.12,0.26)	NA	NA
Conditional height in childhood	NA	NA	0.03 (-0.06,0.12)	0.06 (0,0.12)	-0.11 (-0.52,0.3)	-0.40 (-0.96,0.16)	0.10 (-0.06,0.27)	-0.15 (-0.37,0.07)	0.17* (0.06,0.27)	0.24* (0.13,0.36)	NA	NA
Conditional height in adolescence	NA	NA	-0.08 (-0.18,0.02)	0.08 (-0.01,0.17)	0.25 (-0.19,0.68)	-0.08 (-0.6,0.45)	0.07 (-0.04,0.18)	-0.12 (-0.38,0.14)	-0.03 (-0.17,0.1)	-0.09 (-0.26,0.08)	NA	NA
Conditional relative weight at birth	NA	NA	0.13* (0.05,0.21)	0.10* (0.03,0.17)	-0.08 (-0.37,0.21)	0.42* (0.17,0.66)	0.09 (-0.04,0.22)	0.10 (-0.08,0.27)	0.11* (0.02,0.2)	0.14* (0.04,0.24)	NA	NA
Conditional relative weight in infancy	NA	NA	0.29* (0.21,0.37)	0.29* (0.23,0.36)	0.47 (-0.06,0.99)	0.22 (-0.19,0.63)	0.34* (0.22,0.47)	0.4* (0.23,0.58)	0.31* (0.21,0.41)	0.37* (0.25,0.49)	NA	NA
Conditional relative weight in childhood	NA	NA	0.42* (0.33,0.52)	0.41* (0.33,0.5)	0.35 (-0.41,1.11)	0.20 (-0.35,0.75)	0.44* (0.25,0.64)	0.36* (0.12,0.6)	0.71* (0.59,0.83)	0.70* (0.55,0.85)	NA	NA
Conditional relative weight in adolescence	NA	NA	0.34* (0.28,0.41)	0.38* (0.32,0.44)	0.59* (0.12,1.06)	0.44* (0.09,0.8)	0.36* (0.28,0.45)	0.52* (0.38,0.66)	0.47* (0.38,0.55)	0.47* (0.38,0.57)	NA	NA

[1] Values are standardized linear regression coefficients (βs and 95% CIs). In adjusted analyses we controlled for birth characteristics (gestational age, birth order), maternal characteristics (height, age at childbirth, schooling, marital status), paternal characteristics (age, schooling), income and/or wealth index of child's household at birth, and age in adulthood. In women' models, we adjusted for age of menarche and teenage childbearing. Additionally, we controlled for maternal skin color in Brazil and adjusted for birth year and intervention group (village fixed effects) in Guatemala analysis. * P value < 0.05; SD = Standard Deviation

[2] We used birth length as the anchor in conditional models of sites that collected this measure (Brazil 1993, Guatemala, India, The Philippines)

For peer review only

Supplementary Table 12. Adjusted associations length at birth, conditional growth (height and relative weight) in infancy, childhood, and adolescence with adult body mass index (BMI, SD units), stratified by study site and sex[1]

	Brazil 1982		Brazil 1993		Guatemala		India		The Philippines		South Africa	
	Men (n = 344)	Women (n = 330)	Men (n = 375)	Women (n = 452)	Men (n = 67)	Women (n = 96)	Men (n = 426)	Women (n = 255)	Men (n = 656)	Women (n = 541)	Men (n = 287)	Women (n = 308)
**Conditional growth, z-scores[2]**												
Birth length	NA	NA	-0.01 (-0.06,0.05)	0.1* (0.04,0.16)	0.00 (-0.22,0.22)	0.13 (-0.07,0.33)	0.10* (0.01,0.18)	0.18* (0.08,0.29)	0.08* (0.02,0.15)	0.07 (0,0.14)	NA	NA
Conditional height in infancy	NA	NA	0.07* (0.01,0.12)	0.09* (0.02,0.16)	0.36* (0.03,0.68)	0.15 (-0.14,0.43)	0.12* (0.03,0.22)	0.04 (-0.11,0.18)	0.14* (0.07,0.21)	0.15* (0.07,0.23)	NA	NA
Conditional height in childhood	NA	NA	0.13* (0.05,0.21)	0.14* (0.04,0.25)	-0.12 (-0.56,0.31)	-0.17 (-0.76,0.42)	0.10 (-0.06,0.27)	0.00 (-0.23,0.23)	0.15* (0.05,0.26)	0.27* (0.16,0.39)	NA	NA
Conditional height in adolescence	NA	NA	0.03 (-0.06,0.11)	0.06 (-0.06,0.19)	0.33 (-0.13,0.8)	-0.08 (-0.63,0.48)	0.03 (-0.08,0.14)	-0.03 (-0.3,0.24)	-0.03 (-0.17,0.12)	-0.08 (-0.26,0.09)	NA	NA
Conditional relative weight at birth	NA	NA	0.12* (0.03,0.22)	0.14* (0.03,0.25)	0.04 (-0.27,0.35)	0.35* (0.09,0.61)	0.11 (-0.02,0.24)	0.05 (-0.13,0.23)	0.09 (0,0.19)	0.12* (0.02,0.22)	NA	NA
Conditional relative weight in infancy	NA	NA	0.32* (0.22,0.41)	0.33* (0.23,0.42)	0.50 (-0.05,1.06)	0.06 (-0.37,0.5)	0.30* (0.17,0.42)	0.33* (0.15,0.51)	0.28* (0.18,0.38)	0.31* (0.19,0.43)	NA	NA
Conditional relative weight in childhood	NA	NA	0.48* (0.38,0.57)	0.63* (0.53,0.74)	0.22 (-0.59,1.03)	0.39 (-0.19,0.97)	0.42* (0.23,0.62)	0.13 (-0.12,0.38)	0.65* (0.53,0.78)	0.70* (0.54,0.85)	NA	NA
Conditional relative weight in adolescence	NA	NA	0.49* (0.42,0.55)	0.66* (0.55,0.77)	0.42 (-0.08,0.91)	0.56* (0.18,0.93)	0.35* (0.26,0.44)	0.53* (0.38,0.68)	0.43* (0.34,0.52)	0.44* (0.33,0.54)	NA	NA

[1] Values are standardized linear regression coefficients (βs and 95% CIs). In adjusted analyses we controlled for birth characteristics (gestational age, birth order), maternal characteristics (height, age at childbirth, schooling, marital status), paternal characteristics (age, schooling), income and/or wealth index of child's household at birth, and age in adulthood. In women' models, we adjusted for age of menarche and teenage childbearing. Additionally, we controlled for maternal skin color in Brazil and adjusted for birth year and intervention group (village fixed effects) in Guatemala analysis. * P value < 0.05; SD = Standard Deviation

[2] We used birth length as the anchor in conditional models of sites that collected this measure (Brazil 1993, Guatemala, India, The Philippines)

For peer review only

Supplementary Table 13. Adjusted associations between length at birth, conditional growth (height and relative weight) in infancy, childhood, and adolescence with adult waist circumference (SD units), stratified by study site and sex

	**Brazil 1982**		**Brazil 1993**		**Guatemala**		**India**		**The Philippines**		**South Africa**	
	Men (n = 344)	Women (n = 330)	Men (n = 375)	Women (n = 452)	Men (n = 67)	Women (n = 96)	Men (n = 426)	Women (n = 255)	Men (n = 656)	Women (n = 541)	Men (n = 287)	Women (n = 308)
**Conditional growth, z-scores[2]**												
Birth length	NA	NA	0.00 (-0.07,0.06)	0.08 (-0.02,0.19)	-0.03 (-0.26,0.21)	0.02 (-0.23,0.28)	0.09 (0,0.19)	0.11 (-0.05,0.26)	0.09* (0.02,0.17)	0.02 (-0.09,0.12)	NA	NA
Conditional height in infancy	NA	NA	0.12* (0.03,0.2)	0.09 (-0.04,0.21)	0.31 (-0.06,0.68)	-0.05 (-0.56,0.46)	0.11 (0,0.23)	-0.18 (-0.43,0.06)	0.22* (0.12,0.32)	0.12 (-0.04,0.28)	NA	NA
Conditional height in childhood	NA	NA	0.19* (0.08,0.3)	0.19 (0,0.38)	-0.19 (-0.65,0.28)	-0.18 (-0.83,0.46)	0.12 (-0.07,0.3)	0.07 (-0.27,0.41)	0.24* (0.1,0.38)	0.20 (-0.03,0.43)	NA	NA
Conditional height in adolescence	NA	NA	0.10 (-0.01,0.2)	0.07 (-0.16,0.3)	0.26 (-0.35,0.87)	-0.39 (-1.4,0.62)	0.07 (-0.06,0.19)	-0.21 (-0.65,0.23)	0.13 (-0.04,0.31)	-0.12 (-0.48,0.23)	NA	NA
Conditional relative weight at birth	NA	NA	0.10 (0,0.21)	0.11* (0.01,0.22)	0.08 (-0.25,0.4)	0.4* (0.14,0.67)	0.11 (-0.03,0.25)	0.12 (-0.07,0.31)	0.04 (-0.06,0.14)	0.10 (0.00,0.21)	NA	NA
Conditional relative weight in infancy	NA	NA	0.32* (0.21,0.42)	0.30* (0.21,0.39)	0.49 (-0.1,1.08)	0.11 (-0.34,0.56)	0.24* (0.11,0.38)	0.32* (0.13,0.5)	0.25* (0.14,0.35)	0.24* (0.11,0.37)	NA	NA
Conditional relative weight in childhood	NA	NA	0.45* (0.33,0.56)	0.51* (0.39,0.63)	0.33 (-0.49,1.16)	0.19 (-0.38,0.77)	0.43* (0.23,0.64)	-0.02 (-0.27,0.24)	0.47* (0.33,0.6)	0.57* (0.41,0.73)	NA	NA
Conditional relative weight in adolescence	NA	NA	0.45* (0.37,0.52)	0.58* (0.48,0.68)	0.36 (-0.19,0.91)	0.49* (0.11,0.87)	0.26* (0.16,0.35)	0.42* (0.25,0.58)	0.32* (0.23,0.42)	0.40* (0.30,0.51)	NA	NA

[1] Values are standardized linear regression coefficients (βs and 95% CIs). In adjusted analyses we controlled for birth characteristics (gestational age, birth order), maternal characteristics (height, age at childbirth, schooling, marital status), paternal characteristics (age, schooling), income and/or wealth index of child's household at birth, and age and height in adulthood. In women' models, we adjusted for age of menarche and teenage childbearing. Additionally, we controlled for maternal skin color in Brazil and adjusted for birth year and intervention group (village fixed effects) in Guatemala analysis. * P value < 0.05; SD = Standard Deviation

[2] We used birth length as the anchor in conditional models of sites that collected this measure (Brazil 1993, Guatemala, India, The Philip

For peer review only

Supplementary Table 14. Correlations between adult height and body composition measurements, stratified by study site and sex

	Brazil 1982		Brazil 1993		Guatemala		India		The Philippines		South Africa	
	r	p-value	r	p-value	r	p-value	r	p-value	r	p-value	r	p-value
**Men**												
BMI	0.00	0.944	0.02	0.744	0.01	0.935	-0.01	0.904	0.06	0.103	0.01	0.830
FMI	-0.01	0.888	0.04	0.450	0.10	0.445	0.02	0.704	0.05	0.160	0.04	0.504
FFMI	0.00	0.944	-0.02	0.682	-0.08	0.509	-0.01	0.851	0.07	0.059	0.01	0.872
WC	0.17	0.001	0.17	0.001	0.17	0.163	0.18	<0.001	0.21	<0.001	0.26	<0.001
**Women**												
BMI	-0.04	0.479	-0.09	0.065	0.03	0.806	0.01	0.888	0.05	0.246	-0.06	0.258
FMI	-0.02	0.749	-0.09	0.052	0.04	0.676	0.09	0.167	0.04	0.322	-0.07	0.219
FFMI	-0.08	0.131	-0.05	0.294	-0.01	0.945	-0.15	0.014	0.06	0.157	-0.01	0.794
WC	0.08	0.133	0.06	0.194	0.22	0.03	0.23	<0.001	0.18	<0.001	0.15	0.008

BMI: body mass index; FMI: fat mass index; FFMI: fat-free mass index; WC: waist circumference

For peer review only

STROBE Statement—Checklist of items that should be included in reports of ***cohort studies***

	Item No	Recommendation	Reported on page
**Title and abstract**	1	(*a*) Indicate the study's design with a commonly used term in the title or the abstract	1
		(*b*) Provide in the abstract an informative and balanced summary of what was done and what was found	4
**Introduction**			
Background/rationale	2	Explain the scientific background and rationale for the investigation being reported	6-7
Objectives	3	State specific objectives, including any prespecified hypotheses	7
**Methods**			
Study design	4	Present key elements of study design early in the paper	8
Setting	5	Describe the setting, locations, and relevant dates, including periods of recruitment, exposure, follow-up, and data collection	8
Participants	6	(*a*) Give the eligibility criteria, and the sources and methods of selection of participants. Describe methods of follow-up	8
		(*b*) For matched studies, give matching criteria and number of exposed and unexposed	N/A
Variables	7	Clearly define all outcomes, exposures, predictors, potential confounders, and effect modifiers. Give diagnostic criteria, if applicable	8-10
Data sources/ measurement	8*	For each variable of interest, give sources of data and details of methods of assessment (measurement). Describe comparability of assessment methods if there is more than one group	8, 10-11
Bias	9	Describe any efforts to address potential sources of bias	12-13
Study size	10	Explain how the study size was arrived at	Figure 1
Quantitative variables	11	Explain how quantitative variables were handled in the analyses. If applicable, describe which groupings were chosen and why	6-7
Statistical methods	12	(*a*) Describe all statistical methods, including those used to control for confounding	11-13
		(*b*) Describe any methods used to examine subgroups and interactions	11-13
		(*c*) Explain how missing data were addressed	13
		(*d*) If applicable, explain how loss to follow-up was addressed	N/A
		(*e*) Describe any sensitivity analyses	11
**Results**			
Participants	13*	(a) Report numbers of individuals at each stage of study—eg numbers potentially eligible, examined for eligibility, confirmed eligible, included in the study, completing follow-up, and analysed	13-14
		(b) Give reasons for non-participation at each stage	8
		(c) Consider use of a flow diagram	Figure 1, 14
Descriptive data	14*	(a) Give characteristics of study participants (eg demographic, clinical, social) and information on exposures and potential confounders	13 & Tables 1 and 2
		(b) Indicate number of participants with missing data for each variable of interest	Suppl. Table 1
		(c) Summarise follow-up time (eg, average and total amount)	N/A

For peer review only - http://bmjopen.bmj.com/site/about/guidelines.xhtml

Outcome data	15*	Report numbers of outcome events or summary measures over time	Table 2
Main results	16	(*a*) Give unadjusted estimates and, if applicable, confounder-adjusted estimates and their precision (eg, 95% confidence interval). Make clear which confounders were adjusted for and why they were included	16-21 & Tables 3-4
		(*b*) Report category boundaries when continuous variables were categorized	N/A
		(*c*) If relevant, consider translating estimates of relative risk into absolute risk for a meaningful time period	N/A
Other analyses	17	Report other analyses done—eg analyses of subgroups and interactions, and sensitivity analyses	16-21 & Suppl. Tables
**Discussion**			
Key results	18	Summarise key results with reference to study objectives	22
Limitations	19	Discuss limitations of the study, taking into account sources of potential bias or imprecision. Discuss both direction and magnitude of any potential bias	24-25
Interpretation	20	Give a cautious overall interpretation of results considering objectives, limitations, multiplicity of analyses, results from similar studies, and other relevant evidence	22-24
Generalisability	21	Discuss the generalisability (external validity) of the study results	25
**Other information**			
Funding	22	Give the source of funding and the role of the funders for the present study and, if applicable, for the original study on which the present article is based	26

*Give information separately for exposed and unexposed groups.

Note: An Explanation and Elaboration article discusses each checklist item and gives methodological background and published examples of transparent reporting. The STROBE checklist is best used in conjunction with this article (freely available on the Web sites of PLoS Medicine at http://www.plosmedicine.org/, Annals of Internal Medicine at http://www.annals.org/, and Epidemiology at http://www.epidem.com/). Information on the STROBE Initiative is available at http://www.strobe-statement.org.

For peer review only

For peer review only - http://bmjopen.bmj.com/site/about/guidelines.xhtml

BMJ Open

Growth patterns in childhood and adolescence and adult body composition: a pooled analysis of birth cohort studies from five low- and middle-income countries (COHORTS collaboration)

Journal:	*BMJ Open*
Manuscript ID	bmjopen-2022-068427.R1
Article Type:	Original research
Date Submitted by the Author:	18-Jan-2023

Complete List of Authors:	Poveda Rey, Natalia Elvira; Emory University, Rollins School of Public Health<br>Adair, Linda; University of North Carolina at Chapel Hill, Carolina Population Center<br>Martorell, Reynaldo; Emory University School of Public Health, Hubert Department of Global Health<br>Patel, Shivani; Emory University, Global Health<br>Ramirez-Zea, Manuel; Institute of Nutrition of Central America and Panama (INCAP), INCAP Research Center for the Prevention of Chronic Diseases (CIIPEC)<br>Bhargava, Santosh; Senior Consultant Pediatrics and Founder of the New Delhi Birth Cohort<br>Bechayda, Sonny A; University of San Carlos - Talamban Campus, Office of Population Studies Foundation<br>Carba, Delia B; University of San Carlos - Talamban Campus, Office of Population Studies Foundation<br>Kroker-Lobos, Maria; Institute of Nutrition of Central America and Panama (INCAP), INCAP Research Center for the Prevention of Chronic Diseases (CIIPEC)<br>Horta, Bernardo; Federal University of Pelotas, Post-Graduate Program in Epidemiology<br>Lima, Natália; Federal University of Pelotas<br>Mazariegos, Mónica; Institute of Nutrition of Central America and Panama (INCAP), INCAP Research Center for the Prevention of Chronic Diseases (CIIPEC)<br>Menezes, Ana Maria ; Federal University of Pelotas, Post-Graduate Program in Epidemiology<br>Norris, Shane; University of the Witwatersrand, SAMRC/Wits Developmental Pathways for Health Research Unit, Department of Pediatrics, Faculty of Health Sciences<br>Nyati, Lukhanyo H; University of the Witwatersrand, SAMRC/Wits Developmental Pathways for Health Research Unit, Department of Pediatrics, Faculty of Health Sciences<br>Richter, Linda; University of the Witwatersrand, DSI-NRF Centre of Excellence in Human Development<br>Sachdev, Harshpal; Sitaram Bhartia Institute of Science and Research, Senior Consultant Pediatrics and Clinical Epidemiology<br>Wehrmeister , Fernando ; Universidade Federal de Pelotas, Post-

	Graduate Program in Epidemiology<br>Stein, Aryeh D.; Emory University, Hubert Department of Global Health
**Primary Subject Heading**:	Public health
Secondary Subject Heading:	Epidemiology, Global health
Keywords:	EPIDEMIOLOGY, PUBLIC HEALTH, NUTRITION & DIETETICS

I, the Submitting Author has the right to grant and does grant on behalf of all authors of the Work (as defined in the below author licence), an exclusive licence and/or a non-exclusive licence for contributions from authors who are: i) UK Crown employees; ii) where BMJ has agreed a CC-BY licence shall apply, and/or iii) in accordance with the terms applicable for US Federal Government officers or employees acting as part of their official duties; on a worldwide, perpetual, irrevocable, royalty-free basis to BMJ Publishing Group Ltd ("BMJ") its licensees and where the relevant Journal is co-owned by BMJ to the co-owners of the Journal, to publish the Work in this journal and any other BMJ products and to exploit all rights, as set out in our licence.

The Submitting Author accepts and understands that any supply made under these terms is made by BMJ to the Submitting Author unless you are acting as an employee on behalf of your employer or a postgraduate student of an affiliated institution which is paying any applicable article publishing charge ("APC") for Open Access articles. Where the Submitting Author wishes to make the Work available on an Open Access basis (and intends to pay the relevant APC), the terms of reuse of such Open Access shall be governed by a Creative Commons licence – details of these licences and which Creative Commons licence will apply to this Work are set out in our licence referred to above.

Other than as permitted in any relevant BMJ Author's Self Archiving Policies, I confirm this Work has not been accepted for publication elsewhere, is not being considered for publication elsewhere and does not duplicate material already published. I confirm all authors consent to publication of this Work and authorise the granting of this licence.

For peer review only

Growth patterns in childhood and adolescence and adult body composition: a pooled analysis of birth cohort studies from five low- and middle-income countries (COHORTS collaboration)

Author names:

Natalia E Poveda,[1] Linda S Adair,[2] Reynaldo Martorell,[3] Shivani A Patel,[3] Manuel Ramirez-Zea,[4] Santosh K Bhargava,[5] Sonny A Bechayda,[6] Delia B Carba,[6] María F Kroker-Lobos,[4] Bernardo L Horta,[7] Natalia P Lima,[7] Mónica Mazariegos,[4] Ana M B Menezes,[7] Shane A Norris,[8] Lukhanyo H Nyati,[8] Linda M Richter,[9] Harshpal Singh Sachdev,[10] Fernando C Wehrmeister,[7] Aryeh D Stein,[3] and COHORTS Group[11]

Author affiliations

[1]Nutrition and Health Sciences, Laney Graduate School, Emory University, Atlanta, GA, USA

[2]Department of Nutrition, Gillings School of Global Public Health, University of North Carolina at Chapel Hill, Chapel Hill, NC, USA

[3]Hubert Department of Global Health, Rollins School of Public Health, Emory University, Atlanta GA, USA

[4]INCAP Research Center for the Prevention of Chronic Diseases (CIIPEC), Institute of Nutrition of Central America and Panama (INCAP), Guatemala City, Guatemala

[5]Senior Consultant Pediatrics and Founder of the New Delhi Birth Cohort, D-7 Gulmohar Park, New Delhi 110049, India

[6]Office of Population Studies Foundation, University of San Carlos, Talamban, Cebu City, The Philippines

[7]Post-Graduate Program in Epidemiology, Federal University of Pelotas, Pelotas, Brazil

[8]SAMRC/Wits Developmental Pathways for Health Research Unit, Department of Pediatrics, Faculty of Health Sciences, University of the Witwatersrand, Johannesburg, South Africa.

[9]DSI-NRF Centre of Excellence in Human Development, University of the Witwatersrand, Johannesburg, South Africa

For peer review only

[10]Senior Consultant Pediatrics and Clinical Epidemiology, Sitaram Bhartia Institute of Science and Research, B-16 Qutab Institutional Area, New Delhi, India

[11]Additional members of the COHORTS Group include: **Pelotas Birth Cohorts**: Cesar G Victora; **New Delhi Birth Cohort**: Caroline HD Fall, Clive Osmond, Lakshmy Ramakrishnan, Bhaskar Singh, and Sikha Sinha; **INCAP Nutrition Supplementation Trial Longitudinal Study**: Adela Sanchez; **Cebu Longitudinal Health and Nutrition Survey:** Isabelita Bas, Judith B Borja, Nanette R Lee, Lorna L Perez

Correspondence to:

Aryeh D Stein, PhD

Rollins School of Public Health of Emory University

1518 Clifton Rd NE #7007

Atlanta GA 30322, USA

Aryeh.stein@emory.edu

ORCID iDs

Natalia E Poveda https://orcid.org/0000-0001-6070-362X

Linda S Adair https//orcid.org/ 0000-0002-3670-8073

Reynaldo Martorell https://orcid.org/ 0000-0001-8862-7243

Shivani A Patel https://orcid.org/0000-0003-0082-5857

Manuel Ramirez-Zea https://orcid.org/0000-0001-5107-9175

Sonny A Bechayda https://orcid.org/0000-0003-0076-5276

Bernardo L Horta https://orcid.org/ 0000-0001-9843-412X

Mónica Mazariegos https://orcid.org/0000-0002-2250-3683

Ana M B Menezes https://orcid.org/ 0000-0002-2996-9427

For peer review only

Shane A Norris https://orcid.org/ 0000-0001-7124-3788

Linda M Richter https://orcid.org/ 0000-0002-3654-3192

Harshpal Singh Sachdev https://orcid.org/0000-0002-4956-9391

Fernando C Wehrmeister https://orcid.org/0000-0001-7137-1747

Aryeh D Stein http://orcid.org/0000-0003-1138-6458

For peer review only

ABSTRACT

Objective We examined associations among serial measures of linear growth and relative weight with adult body composition.

Design Secondary data analysis of prospective birth cohort studies

Settings Six birth cohorts from Brazil, Guatemala, India, Philippines and South Africa.

Participants 4173 individuals followed from birth to ages 22-46 years with complete and valid weight and height at birth, infancy, childhood and adolescence, and body composition in adult life.

Exposures Birth weight and conditional size (standardized residuals of height representing linear growth and of relative weight representing weight increments independent of linear size) in infancy, childhood, and adolescence

Primary outcome measures Body mass index (BMI), fat mass index (FMI), fat-free mass index (FFMI), fat mass/fat-free mass ratio (FM/FFM), and waist circumference in young and mid-adulthood

Results In pooled analyses, a higher birthweight and relative weight gains in infancy, childhood, and adolescence were positively associated with all adult outcomes. Relative weight gains in childhood and adolescence were the strongest predictors of adult body composition [β (95%CI) among men: FMI (childhood: 0.41 (0.26 to 0.55); adolescence: 0.39 (0.27 to 0.50)), FFMI (childhood: 0.50 (0.34 to 0.66); adolescence: 0.43 (0.32 to 0.55)), FM/FFM (childhood: 0.31 (0.16 to 0.47); adolescence: 0.31 (0.19 to 0.43))]. Among women, similar patterns were observed, but, effect sizes in adolescence were slightly stronger than in childhood. Conditional height in infancy was positively associated with FMI [men: 0.08 (0.03 to 0.14)]; women: 0.11 (0.07 to 0.16)]. Conditional height in childhood was positively but weakly associated with women's adiposity. Site- and sex-stratified analyses showed consistency in the direction of estimates, although there were differences in their magnitude.

Conclusions Prenatal and postnatal relative weight gains were positive predictors of larger body size and increased adiposity in adulthood. A faster linear growth in infancy was a significant but weak predictor of higher adult adiposity.

Keywords: fat mass, fat-free mass, body mass index, waist circumference, growth, conditional height, conditional relative weight, cohort studies

Strengths and limitations of this study

- We analyzed six well-characterized population-based prospective birth cohorts from five low- and middle-income countries.
- We considered a two-compartment model of body composition (FMI and FFMI), that were measured with gold standards and/or validated techniques at each study site.
- The use of conditional growth measures allowed us to break the high correlations among serial measures of weight and height to study the independent roles of linear growth and relative weight at specific age intervals.
- Attrition might be one source of selection bias given the long-lasting follow-up of these birth cohorts.
- Residual confounding by unmeasured characteristics such as diet, reproductive history and other life-style factors might bias the estimates.

Introduction

Low and middle-income countries (LMICs) have witnessed an increase in the prevalence of cardiometabolic risk factors such as diabetes, hypertension, and obesity in the adult population.[1-3] Overweight and obesity are not only metabolic outcomes but also serve as risk factors for other cardiometabolic diseases.[4] The developmental origins of health and disease (DOHaD) hypothesis proposes that anatomic, physiologic, and metabolic adaptations in early life have long-lasting effects on the onset of adult cardiometabolic disease.[5]

For peer review only

Adaptations in body composition might have an important role in the programming of cardiometabolic disease "*through its own programming by early growth, and/or through being a mediator of the programming process*".[6] A recent review concluded that associations between infant growth and long-term cardiometabolic outcomes might be mediated by body composition after estimates were attenuated when controlling for current BMI.[7] Similarly, other systematic reviews and one meta-analysis showed that childhood obesity is a risk factor of cardiometabolic outcomes in adulthood. However, results were inconclusive in demonstrating whether or not this excessive weight gain in childhood was an independent factor or whether most of this effect was mediated through contemporary adult BMI.[8-10]

BMI in infancy, childhood, and adolescence has a moderate to strong tracking into adult adiposity.[8, 11] However, the discrimination between body compartments such as fat and lean mass, which have different metabolic functions, is more relevant to understand cardiometabolic risk than BMI alone.[12] The study of the role of growth across the lifespan on body composition has demonstrated similarities and differences when comparing body compartments. Overall studies have shown that birth weight is positively associated with lean mass later in life,[6] and that it is a stronger predictor of fat-free mass (FFM) than fat mass (FM).[13, 14] Less consistency (positive, inverse and null findings) has been observed in studies that have examined the association between birthweight (low and high) with adiposity in childhood, adolescence, and adulthood.[6, 15, 16] Additionally, studies in high-income countries (HIC) have shown that a rapid weight gain (≥ 0.67 SD) in infancy (first 24 months after birth) are associated with higher obesity and fat mass in childhood and adulthood.[7, 17] Whereas findings in LMICs have shown that weight gain in infancy is associated with higher FM and FFM in adult life, being a stronger predictor of FFM.[6, 7, 17]

There is a paucity of studies about the long-term effects of growth across the life span on adult body composition. Available evidence has primarily examined associations between prenatal and postnatal growth (mainly in infancy) with body composition in childhood and adolescence. In a recent literature

For peer review only - http://bmjopen.bmj.com/site/about/guidelines.xhtml

review of publications between 2003 and 2018, only 6 out of 39 studies examined associations between weight gain during infancy and body composition in adult participants from LMICs.[7] Less evidence is available about the role of linear growth (height) on adult body composition, and findings are mixed.[7] There is a need for investigating the independent long-term role of linear growth and relative weight gain across the life course on body composition in young and mid-adulthood; stages in which the accumulation of cardiometabolic risk is higher.

Pooled analyses of COHORTS, a consortium of birth cohorts from five LMICs,[18] showed that higher weight at birth, in infancy, and in mid-childhood were associated with a higher risk of adult overweight (ages 18-31).[13] These pooled analyses also showed that weight at birth and in infancy were stronger predictors of FFM than of FM in adult life, while weight gain in mid-childhood was a stronger predictor of FM than FFM.[13, 14] We expand on these previous studies by 1) analyzing for the first time the period between childhood and adolescence, 2) examining the long term-role of child growth on body composition on young and mid-adulthood (ages 22-46) given that in previous analysis some study participants were adolescents, and 3) using body composition indices (height squared adjustment) to assess body compartments (weight, fat mass, and fat-free mass) independent of linear size. Specifically, we now examine the associations of linear growth and relative weight at birth, infancy, childhood, and adolescence with measures of body size and composition (BMI, FMI, FFMI, FM/FFM ratio, and waist circumference) in young and mid-adulthood.

Methods

Study design and participants

We conducted a secondary data analysis of COHORTS, the Consortium of Health-Oriented Research in Transitional Societies (COHORTS).[18] This consortium comprises six prospective birth cohorts in five study sites: 1) The 1982[19, 20] and 1993 Pelotas Birth Cohorts in Brazil;[21] 2) The Institute of Nutrition of Central America and Panama Nutrition Trial and Longitudinal Study in Guatemala;[22]

For peer review only

3)The New Delhi Birth Cohort in India;[23] 4) The Cebu Longitudinal Health and Nutrition Survey in the Philippines;[24] and The Birth to Twenty Plus Cohort in South Africa.[25] Table 1 presents a general description of each birth cohort.

We selected one analytic sample for each study site (Figure 1). Of a total of 27437 participants, we excluded participants with missing body composition in adulthood (n=16029), implausible values of BMI ≤ 12 or ≥ 60 Kg/m^2 and waist circumference ≤ 51 or ≥ 190 cm (n=1), and pregnancy at the moment of the anthropometric assessment (n=135). We also excluded those individuals with missing anthropometric data at birth, childhood, or adolescence (n=6967), implausible values of weight and height considering the WHO Z-scores thresholds (n=168),[26, 27] and pregnancy at the adolescence measurement (n=47).

Variable specification

Outcomes: Adult body composition

Study sites applied different standard methods to measure body composition in adult participants such as dual-energy X-ray absorptiometry (DEXA), air-displacement plethysmography (BodPod), deuterium oxide dilution technique, bioimpedance,[19, 28-30] and anthropometry (Table 1). Our outcomes measures are: 1) BMI (weight in kg/height in meters^2) as a proxy of body size; 2) FMI to represent the fat compartment adjusted for size (fat mass in kg/height in meters^2); 3) FFMI to represent the lean compartment adjusted for size (fat-free mass in kg/height in meters^2); 4) FM/FMM ratio (a higher ratio indicates a greater increment in the fat mass component); and 5) the waist circumference (centimeters) as a proxy of abdominal adiposity. We transformed these outcomes measures into Z-scores (SD: standard deviation units) to compare effect sizes within strata of site and sex, and among the measures.

For peer review only

202 Table 1. General characteristics of the six birth cohorts and body composition methods used at each study site

203

Country (cohort)	Study design	Cohort enrollment	Age at enrollment	Initial sample	Analytic sample	Age & year most recent follow-up	Body composition methods
Brazil (1982 Pelotas Birth Cohort)	Prospective cohort	1982	Birth	5914	674	30 (2012)	BodPod and anthropometry
Brazil (1993 Pelotas Birth Cohort)	Prospective cohort	1993	Birth	5249	827	22.5 (2015)	BodPod and anthropometry
Guatemala (INCAP Nutrition Trial and Longitudinal Study)	Community trial	1969-77	Pregnancy – 7 years	2392	163	45.5 (2015-17)	Deuterium oxide dilution technique and anthropometry
India (NDBC)	Prospective cohort	1969-72	Before pregnancy	8181	681	46 (2016-19)	Bioimpedance and anthropometry
The Philippines (CLHNS)	Prospective cohort	1983-84	Birth	3080	1197	34.5 (2018)	Bioimpedance and anthropometry
South Africa (Birth to Twenty Plus Cohort)	Prospective cohort	1990	Pregnancy	3273	595	22 (2012)	DEXA (Dual-energy X-ray absorptiometry) and anthropometry

204 INCAP: Institute of Nutrition of Central America and Panama; NDBC: New Delhi Birth Cohort; CLHNS: Cebu Longitudinal Health and Nutrition Survey

205 BodPod: air-displacement plethysmography

For peer review only

Anthropometry at birth, infancy, childhood, and adolescence

Birthweight was measured in all study sites, while birth length was available only in four of the six study sites (except Brazil 1982 and South Africa). In Brazil, anthropometric data at birth was measured at the hospitals by the research team (Brazil 1993) or by the hospital personnel (Brazil 1982) using pediatric scales.[31] In Guatemala, the study team measured weight within the first 48 hours after birth and length within the first 15 days after the delivery, using a beam balance and a measuring board.[32] In India, trained project staff measured birth and length within the first 72 hours of life using standard instruments.[33] In the Philippines, the 60% of the participants who were born at home were weighted by local birth attendants using hanging scales and the remaining participants were measured in hospitals, and birth length was measured within the first 6 days of life.[34] In South Africa, birthweight measured by hospital personnel was obtained from hospital records.

Trained research staff at each study site followed standard procedures to take anthropometric measures of length and/or height and weight in subsequent follow-ups (childhood and adolescence). Growth measures that represent linear growth (length/height) and mass accretion independent of linear size (relative weight) were measured in different periods at each study site. We identified four common or near ages where anthropometric data was available across all cohorts: 1) birth, 2) infancy (all study sites registered weight and length data at 24 months of age except Brazil 1993 that collected these data at 12 months), 3) childhood (five study sites collected growth data at 48 months of age, but Philippines that collected data at 102 months), and 4) adolescence (all study sites collected anthropometric data at180 months of age). To be able to compare growth measures between study sites, we estimated sex-specific length/height-for-age and weight-for-age Z-scores (HAZ and WAZ, respectively) using the WHO growth standards.[35]

Exposures: Conditional growth (height and relative weight)

Repeated measures of linear growth and relative weight are strongly correlated. To eliminate this correlation, we estimated conditional size measures (standardized residuals) by regressing current HAZ

For peer review only

on previous HAZ and WAZ measures, and regressing current WAZ on current HAZ and all prior HAZ and WAZ measures, within strata of site and sex. This approach allowed us to identify if children's growth deviates from their expected length/height or weight as estimated based on their own previous growth measurements and the growth of other children. We used birthweight as the anchor to estimate conditional growth measures (height and relative weight) in the main analysis of all cohorts. In separate models, we derived conditional growth measures using birth length as an anchor in the four study studies in which this variable was available. We estimated conditional height and conditional relative weight in four age intervals across all study sites: 1) prenatal (from conception to birth captured though birthweight and birth length); 2) infancy (birth to 12 months in Brazil 1993, and birth to 24 months in the other study sites); 3) childhood (12-48 months in Brazil 1993, 24-48 months in Brazil 1982, Guatemala, India, and South Africa, and 24-102 months in Philippines); and 4) adolescence (48-180 months in five study sites and 102-180 months in Philippines).

Covariates: We identified potential confounders available at each study site such as birth characteristics (gestational age, birth order), income and/or wealth index of child's household at birth, maternal characteristics (height, age at first childbirth, schooling attainment, marital status), paternal characteristics (age, schooling attainment), reproductive factors (age at menarche), and age in adulthood (Supplementary Table 1).

Statistical analyses

We used R version 4.0 for all statistical analyses. Statistical significance was established at a p-value < 0.05.

Site-specific regression models

We used multiple linear regressions with standardized coefficients to estimate the associations between growth patterns at the four age intervals and adult body composition since all outcomes were continuous and without major violations of the normal distribution (graphical examination). We built site-specific

For peer review only

models stratified by sex given the biological differences in body composition.[36] We ran two sets of models: 1) Unadjusted models for all study sites except Guatemala, in which we minimally adjust for birth year and intervention group considering the initial study design of this cohort;[22] 2) Fully adjusted models controlling for potential confounders available at each study site (Supplementary Table 1). Additionally, we adjusted for adult height in waist circumference models, for age at menarche and teenage childbearing in models of women, and for maternal skin color in both Brazil cohorts as one proxy of socioeconomic status. In Brazil 1993 models, we used survey procedures given that this cohort collected data in subsamples (all children with low-weight at birth and a random sample of other children) at 12 and 48 months of age.[31] After a visual examination of effect sizes from both sets of models at each study site, we observed slight changes in magnitude but not in direction (results not presented). We present the fully adjusted models to account for the confounding effect of this group of covariates.

Pooling Effect Sizes

The cohorts come from different study sites and data were collected in different periods, resulting in heterogeneity among site-specific estimates. We used a random-effects meta-analysis approach to pool effect sizes to account for this underlying heterogeneity. Given that our outcomes were continuous, we used the restricted maximum likelihood estimator (REML) to calculate the between-study heterogeneity (variance of the distribution of true effect sizes).[37] We also applied Knapp-Hartung adjustments in the estimation of confidence intervals of pooled effects. We estimated Cochran's Q with its corresponding p-value to test whether there was heterogeneity between study sites, and calculated the I^2 statistic to quantify the magnitude of between-study heterogeneity. As a rule of thumb an I^2 equal to 25% is interpreted as low heterogeneity, 50% as moderate heterogeneity, and 75% as substantial heterogeneity.[37] We used the Metafor package in R to run these analyses.

Multiple imputation

To deal with missing data, we built sex-specific multiple imputation models at each study site. Missing data patterns are presented in Supplementary Table 1. We assumed that the underlying missing pattern was missing at random (MAR). In brief, we used the R package Multivariate Imputation by Chained

For peer review only

Equations (MICE) to generate different multiple imputed datasets with a maximum of 50 iterations. We estimated the number of imputations based on the highest percentage of missingness within strata of site and sex. In our imputation models, we included all covariates used in the main analyses and the outcome variables. We used the "with and pool" functions to run the main analyses in each imputed dataset and to combine the estimates into a single result with its corresponding standard errors and confidence intervals.

Patient and public involvement Patients and/or the public were not involved in the design, or conduct, or reporting, or dissemination plans of our research

Results

We analyzed data from a combined analytic sample of 4137 participants with complete and valid information of body composition in adulthood and anthropometry across the life course (Figure 1). The comparison of general characteristics between participants included in the final analytic samples versus those excluded only showed significant differences in a couple of variables in selected cohorts (Supplementary Table 2). General characteristics of the six cohorts, and anthropometric characteristics at birth and adulthood of men and women at each study site are presented in Tables 1 and 2, respectively. Participant's average adult age ranged from 22 years in Brazil 1993 and South Africa to 45-46 years in Guatemala and India. India had the lowest mean birth weight compared with other study sites. The proportion of excess weight and adiposity were correlated with the age of the cohorts, with the oldest cohorts having the greatest proportion of BMI ≥ 25 kg/m^2 and the highest FMI. Among men, the proportion of excess weight ranged from 16.0% in South Africa to 73.2% in India, and among women ranged from 44.5% in Brazil 1993 to 85.4% in Guatemala. Participants from Guatemala, India, and Brazil 1982 had the highest FMI (women above 10 kg/m^2 and men above 7 kg/m^2). FFMI ranged from 15.5 to 20.2 kg/m^2 in men, and from 13.7 to 17.7 kg/m^2 in women. Other characteristics (at birth, childhood and parental) of study participants at each study site are presented in Supplementary Table 3.

For peer review only

Table 2. Anthropometric characteristics of participants included in the analysis, stratified by study site and sex[1]

	Brazil 1982		Brazil 1993		Guatemala		India		The Philippines		South Africa	
	Men (n=344)	Women (n=330)	Men (n=375)	Women (n=452)	Men (n=67)	Women (n=96)	Men (n=426)	Women (n=255)	Men (n=656)	Women (n=541)	Men (n=287)	Women (n=308)
Birth length (cm)	NA	NA	49.4 (2.3)	48.4 (2.1)	49.6 (2.6)	48.5 (2.2)	48.7 (2.0)	47.9 (2.1)	49.3 (2.0)	48.7 (2.0)	NA	NA
Birth weight (kg)	3.3 (0.6)	3.2 (0.5)	3.3 (0.5)	3.1 (0.5)	3.1 (0.5)	3.0 (0.5)	2.9 (0.4)	2.7 (0.4)	3.0 (0.4)	3.0 (0.4)	3.1 (0.5)	3.0 (0.5)
Adult height (cm)	174.9 (7.1)	161.9 (6.2)	174.8 (7.6)	160.8 (6.6)	164.7 (5.7)	152.0 (4.8)	169.9 (6.8)	154.5 (5.5)	162.8 (5.6)	150.9 (5.5)	171.5 (6.2)	159.7 (6.4)
Adult weight (kg)	82.8 (16.3)	70.1 (16.6)	75.8 (16.1)	66.4 (15.6)	71.4 (14.3)	69.4 (11.8)	80.6 (15.0)	70.0 (12.5)	66.1 (12.8)	57.3 (11.9)	62.9 (11.3)	64.6 (14.9)
Adult BMI (kg/m^2)	27.0 (4.8)	26.7 (6.1)	24.6 (4.7)	25.5 (5.9)	26.3 (4.8)	30.0 (4.7)	27.9 (4.7)	29.3 (4.8)	24.9 (4.4)	25.2 (5.0)	21.4 (3.5)	25.3 (5.6)
Adult excess weight[2]	215 (62.5%)	171 (51.8%)	160 (40.4%)	195 (44.5%)	42 (62.7%)	82 (85.4%)	312 (73.2%)	208 (81.6%)	297 (45.3%)	256 (47.3%)	46 (16.0%)	142 (46.1%)
Adult waist circumference (cm)	89.9 (11.5)	80.8 (11.7)	82.2 (10.8)	77.3 (11.6)	93.7 (10.8)	102.8 (10.7)	100.6 (12.0)	91.8 (10.5)	82.1 (10.8)	80.8 (11.5)	75.8 (8.6)	82.4 (13.5)
Adult fat mass index (FMI, kg/m^2)	7.1 (3.6)	10.5 (4.7)	5.3 (3.7)	9.6 (4.5)	7.7 (2.8)	12.7 (3.1)	7.7 (2.8)	11.6 (3.4)	5.5 (2.5)	9.0 (3.5)	4.0 (2.1)	9.6 (3.9)
Adult fat-free mass index (FFMI, kg/m^2)	19.9 (2.1)	16.2 (1.9)	19.2 (1.9)	15.8 (1.9)	18.5 (2.6)	17.3 (2.2)	20.2 (2.2)	17.7 (1.7)	19.3 (2.0)	16.1 (1.4)	15.5 (1.8)	13.7 (1.9)
Adult fat mass/Fat-free mass ratio	0.4 (0.2)	0.6 (0.2)	0.3 (0.2)	0.6 (0.2)	0.4 (0.1)	0.7 (0.1)	0.4 (0.1)	0.6 (0.2)	0.3 (0.1)	0.5 (0.2)	0.3 (0.1)	0.7 (0.2)

[1] Values are means (SD) or n (percentages). Sample sizes (n) refer to participants with non-missing data for outcomes (adult body composition), and non-missing data for exposures (growth measures). NA, not available data.
[2] Excess weight defined as a BMI $\geq$ 25 kg/m^2

For peer review only

For peer review only - http://bmjopen.bmj.com/site/about/guidelines.xhtml

313 ***Conditional relative weight and adult body composition***

Table 3 shows the pooled adjusted associations of conditional growth with adult FMI and FFMI. Birth weight and conditional relative weight in infancy, childhood, and adolescence were positively associated with FMI and FFMI in adulthood. A higher birth weight was a predictor of higher FMI (men: 0.07 [95%CI: 0.01,0.14]; women: 0.09 [95%CI: 0.01,0.17]) and FFMI (men: 0.11 [95%CI: 0.05, 0.18]; women: 0.12 [95%CI: 0.05, 0.19]). An increment of one SD in conditional relative weight in infancy was associated with an increment of 0.2-0.3 SD in adult FMI, while for every SD increment in conditional weight in childhood or adolescence there was a 0.4-0.5 SD increase in FMI in adulthood. Similarly, the associations of relative weight gain with adult FFMI were positive and stronger as boys and girls grew older. Increments in conditional relative weight by one SD in infancy, childhood, and adolescence were associated with higher FFMI by 0.3, 0.4-0.5, and 0.4 SD units in adult life, respectively. Table 4 shows the pooled adjusted associations of conditional growth with adult FM/FFM ratio. Birthweight was not significantly associated with FM/FFM ratio in adult life. Conditional relative weight in infancy, childhood and adolescence were associated with a higher FM/FMM ratio with increments ranging from 0.17 to 0.43 for every SD in conditional weight.

For peer review only

Table 3. Pooled adjusted associations between weight at birth, conditional growth (height and relative weight) in infancy, childhood, and adolescence with adult fat mass index, fat-free mass index and fat mass/fat-free mass ratio, stratified by sex[1]

	FMI (SD units)						FFMI (SD units)					
	Men (n = 2155)			Women (n = 1982)			Men (n = 2155)			Women (n = 1982)		
	β (95%CI)	I^2 statistic (%)[3]	Cochran's Q test (P value)	β (95%CI)	I^2 statistic (%)	Cochran's Q test (P value)	β (95%CI)	I^2 statistic (%)	Cochran's Q test (P value)	β (95%CI)	I^2 statistic (%)	Cochran's Q test (P value)
**Conditional growth, z-scores[2]**												
Birth weight	0.07* (0.01,0.14)	42.4	0.147	0.09* (0.01,0.17)	57.4	0.044	0.11* (0.05,0.18)	47.0	0.070	0.12* (0.05,0.19)	9.0	0.040
Conditional relative weight in infancy	0.23* (0.18,0.27)	0.0	0.868	0.26* (0.19,0.33)	0.1	0.498	0.30* (0.23,0.37)	24.5	0.297	0.30* (0.20,0.39)	50.3	0.104
Conditional relative weight in early/mid-childhood	0.41* (0.26,0.55)	61.9	0.020	0.46* (0.21,0.72)	83.9	<0.001	0.50* (0.34,0.66)	75.9	0.001	0.42* (0.17,0.67)	92.2	<0.001
Conditional relative weight in adolescence	0.39* (0.27,0.50)	77.4	0.001	0.53* (0.44,0.61)	50.0	0.101	0.43* (0.32,0.55)	81.9	<0.001	0.39* (0.13,0.66)	95.1	<0.001
Conditional height in infancy	0.08* (0.03,0.14)	21.2	0.260	0.11* (0.07,0.16)	12.9	0.558	0.09 (0.00,0.19)	69.2	0.003	0.09 (-0.01,0.20)	78.5	0.001
Conditional height in early/mid-childhood	0.08 (-0.02,0.18)	42.1	0.156	0.14* (0.04,0.24)	31.2	0.285	0.06 (-0.06,0.17)	58.4	0.039	0.12 (-0.14,0.38)	90.0	<0.001
Conditional height in adolescence	0.02 (-0.08,0.12)	16.1	0.192	0.01 (-0.13,0.15)	56.1	0.060	0.00 (-0.08,0.08)	17.6	0.314	0.06 (-0.16,0.28)	89.3	<0.001

[1] Values are standardized linear regression coefficients (βs and 95% CIs). FMI, fat mass index; FFMI, fat-free mass index. In adjusted analyses we controlled for birth characteristics (gestational age, birth order), maternal characteristics (height, age at childbirth, schooling, marital status), paternal schooling, income and/or wealth index of child's household at birth, and age in adulthood. In women' models, we adjusted for age of menarche and teenage childbearing. Additionally, we controlled for maternal skin color in both Brazil cohorts and adjusted for birth year and intervention group (village fixed effects) in Guatemala analysis. * P value < 0.05.

[2] We used relative birth weight as the anchor in all conditional models given that this measure was available across all study sites. In the supplementary section, we present similar models using birth length as the anchor in the sites that collected this measure.

[3] As a rule of thumb I^2 = 25% is interpreted as low heterogeneity, 50% moderate heterogeneity, and 75% substantial heterogeneity

For peer review only - http://bmjopen.bmj.com/site/about/guidelines.xhtml

Table 4. Pooled adjusted associations between weight at birth, conditional growth (height and relative weight) in infancy, childhood, and adolescence with adult fat mass/fat-free mass ratio, stratified by sex[1]

	FM/FFM (SD units)					
	Men (n = 2155)			Women (n = 1982)		
	β (95%CI)	I^2 statistic (%)[3]	Cochran's Q test (P value)	β (95%CI)	I^2 statistic (%)	Cochran's Q test (P value)
**Conditional growth, z-scores[2]**						
Birth weight	0.06 (-0.03,0.14)	64.3	0.032	0.06 (-0.03,0.15)	68.0	0.013
Conditional relative weight in infancy	0.17* (0.12,0.23)	0.0	0.685	0.20* (0.09,0.31)	32.7	0.108
Conditional relative weight in early/mid-childhood	0.31* (0.16,0.47)	65.9	0.009	0.37* (0.07,0.66)	87.5	<0.001
Conditional relative weight in adolescence	0.31* (0.19,0.43)	78.0	0.002	0.43* (0.39,0.48)	0.0	0.803
Conditional height in infancy	0.07* (0.02,0.12)	25.0	0.336	0.10* (0.03,0.16)	37.6	0.181
Conditional height in early/mid-childhood	0.08 (0.00,0.17)	28.7	0.313	0.16* (0.08,0.25)	15.1	0.477
Conditional height in adolescence	0.02 (-0.08,0.13)	27.2	0.124	-0.01 (-0.19,0.17)	72.0	0.002

[1] Values are standardized linear regression coefficients (*β*s and 95% CIs). FM/FFM, fat mass/fat-free mass ratio. In adjusted analyses we controlled for birth characteristics (gestational age, birth order), maternal characteristics (height, age at childbirth, schooling, marital status), paternal schooling, income and/or wealth index of child's household at birth, and age in adulthood. In women' models, we adjusted for age of menarche and teenage childbearing. Additionally, we controlled for maternal skin color in both Brazil cohorts and adjusted for birth year and intervention group (village fixed effects) in Guatemala analysis. * P value < 0.05.

[2] We used relative birth weight as the anchor in all conditional models given that this measure was available across all study sites. In the supplementary section, we present similar models using birth length as the anchor in the sites that collected this measure.

[3] As a rule of thumb I^2 = 25% is interpreted as low heterogeneity, 50% moderate heterogeneity, and 75% substantial heterogeneity

For peer review only

354 The between-study heterogeneity assessment showed low or moderate heterogeneity across almost all associations of conditional growth with FMI and FM/FFM, except conditional relative weight in childhood among women (I^2=83.9%) and conditional relative weight in adolescence among men (I^2=78.0%) (Tables 3 and 4). The between-study heterogeneity assessment of FFMI models showed substantial variability in estimates of conditional relative weight in childhood and adolescence among women and men, and conditional height in childhood and adolescence among women ($I^2 \geq 75\%$) (Table 3).

Site-specific estimates were consistent in magnitude and direction. Birth weight was positively associated with adult FMI in men and women from India and the Philippines, and in women from Brazil 1993. Conditional relative weights in infancy, childhood, and adolescence were positively associated with adult FMI among men and women across all cohorts (Supplementary Table 4). Site-specific analyses showed that birthweight was a predictor of a higher adult FFMI among men and women from four out of the six cohorts (Brazil 1982, India, the Philippines, and South Africa), and among women from Brazil 1993 and Guatemala. Across all cohorts but Guatemala, a higher relative weight gain in childhood was significantly associated with a higher FFMI. Conditional relative weight in adolescent was a strong and significant predictor of a higher FFMI in adult life among all study sites and both sexes (Supplementary Table 5). Birthweight was positively and significantly associated with FM/FFM among men and women from India and the Philippines, and women from Brazil 1993. Conditional relative weights in infancy, childhood, and adolescence were positively associated with FM/FFM across all study sites with a few exceptions (Supplementary Table 6).

Table 5 shows the pooled adjusted associations of conditional growth with adult BMI and waist circumference. Birth weight and all conditional relative weight measures were positively associated with BMI and waist circumference among adult men and women. Effect sizes at birth were small but significant predictors of BMI and waist circumference. Associations strengthened with age at

For peer review only

measurement; for example, men's BMI increased by 0.29 [95%CI: 0.24, 0.35], 0.50 [95%CI: 0.37, 0.64] and 0.47 [95%CI: 0.35, 0.60] SD units per each SD in conditional relative weight in infancy, childhood, and adolescence, respectively. Similar patterns were observed in women. Relative weight gains in infancy and childhood were associated with increments in waist circumference ranging from 0.25-0.26 and 0.40-0.43 SD among adult men and women, respectively. Increments in relative weight during adolescence predicted increments in waist circumference by 0.39 [95%CI: 0.27, 0.52] SD in men and 0.49 [95%CI: 0.42, 0.56] SD units in women. The between-study heterogeneity was low or moderate for most of the associations, except some associations of conditional relative weight in childhood and adolescence (heterogeneities above 75%) (Table 5).

Site-specific analyses of BMI and waist circumference showed similar effect sizes in direction and magnitude as pooled estimates. Relative weight gains in infancy, childhood, and adolescence were predictors of higher BMI and waist circumference in adulthood across study sites, with a few exceptions (Supplementary Tables 7 and 8).

For peer review only

Table 5. Pooled adjusted associations between weight at birth, conditional growth (height and relative weight) in infancy, childhood, and adolescence with adult body mass index and waist circumference, stratified by sex[1]

	BMI (SD units)						Waist circumference (SD units)					
	Men (n = 2155)			Women (n = 1982)			Men (n = 2155)			Women (n = 1982)		
	β (95%CI)	I^2 statistic (%)[3]	Cochran's Q test (P value)	β (95%CI)	I^2 statistic (%)	Cochran's Q test (P value)	β (95%CI)	I^2 statistic (%)	Cochran's Q test (P value)	β (95%CI)	I^2 statistic (%)	Cochran's Q test (P value)
**Conditional growth, z-scores[2]**												
Birth weight	0.10* (0.05,0.14)	13.7	0.454	0.12* (0.03,0.20)	47.0	0.056	0.08* (0.03,0.12)	0.0	0.573	0.09* (0.01,0.16)	0.1	0.260
Conditional relative weight in infancy	0.29* (0.24,0.35)	0.0	0.602	0.30* (0.24,0.36)	0.0	0.634	0.26* (0.18,0.33)	17.9	0.345	0.25* (0.19,0.32)	0.0	0.638
Conditional relative weight in early/mid-childhood	0.50* (0.37,0.64)	58.6	0.044	0.53* (0.31,0.75)	78.4	0.001	0.43* (0.35,0.52)	0.0	0.551	0.40* (0.16,0.63)	79.3	0.002
Conditional relative weight in adolescence	0.47* (0.35,0.60)	85.5	<0.001	0.58* (0.47,0.69)	69.4	0.006	0.39* (0.27,0.52)	79.9	<0.001	0.49* (0.42,0.56)	30.0	0.285
Conditional height in infancy	0.10* (0.03,0.16)	36.5	0.097	0.12* (0.08,0.16)	0.0	0.736	0.12* (0.03,0.21)	48.2	0.089	0.08 (-0.05,0.20)	19.8	0.216
Conditional height in early/mid-childhood	0.07 (-0.04,0.19)	58.4	0.043	0.12 (-0.01,0.25)	53.2	0.066	0.11 (-0.04,0.26)	57.3	0.04	0.14* (0.06,0.23)	0.0	0.866
Conditional height in adolescence	0.01 (-0.07,0.09)	0.0	0.307	0.01 (-0.1,0.13)	37.3	0.225	0.08 (0.00,0.16)	0.0	0.596	-0.04 (-0.26,0.18)	41.7	0.161

[1] Values are standardized linear regression coefficients (βs and 95% CIs). BMI, body mass index. In adjusted analyses we controlled for birth characteristics (gestational age, birth order), maternal characteristics (height, age at childbirth, schooling, marital status), paternal schooling, income and/or wealth index of child's household at birth, and age in adulthood. In women' models, we adjusted for age of menarche and teenage childbearing. Additionally, we controlled for maternal skin color in both Brazilian cohorts and adjusted for birth year and intervention group (village fixed effects) in Guatemala analysis. * P value < 0.05.

[2] We used relative birth weight as the anchor in all conditional models given that this measure was available across all study sites. In the supplementary section, we present similar models using birth length as the anchor in the sites that collected this measure.

[3] As a rule of thumb: $I^2 = 25\%$ is interpreted as low heterogeneity, 50% moderate heterogeneity, and 75% substantial heterogeneity.

For peer review only

Conditional height and adult body composition

Conditional height in infancy was positively associated with FMI and FM/FFM ratio in adult life, but effect sizes were small (around 0.1) (Tables 3 and 4). None of the conditional height variables were significantly associated with adult FFMI (Table 3). A high conditional height in infancy was a significant but weak predictor of higher adult BMI among men and women, and waist circumference among women (increments of 0.10-0.12 SD units) (Table 5). Conditional height in childhood was positively but weakly associated with women's FMI, FM/FFM ratio, and waist circumference (Tables 3, 4 and 5).

We used conditionals derived from birth length as the anchor in models in four of the study sites. In these pooled analyses, we found that birth length was a weak but statistically significant predictor of higher FFMI and BMI in adult women (Supplementary Tables 9 and 10). Other effect sizes from these models were consistent in direction and magnitude with effect sizes observed in the main models (birth weight as anchor). Site-specific analysis using birth length as anchor are presented in Supplementary Tables 11-14.

Body size and composition indices should control for allometric scaling (differences in body compartments given differences in linear size) and should be independent of their denominator (height).[38, 39] Thus, to assess the statistical validity of body size and composition indices, we examined the correlation between these indices with adult height. We observed that the three indices (BMI, FMI, and FFMI) were not significantly correlated with height across all study sites and sexes (correlation coefficients ranged from -0.15 to 0.10). The correlation coefficients of waist circumference with adult height were significant but weak (0.15-0.26) (Supplementary Table 15).

Discussion

In a pooled analysis of six prospective birth cohorts from five LMICs, we found that a higher birthweight predicted higher adult FFMI, FMI, BMI and waist circumference, but not adiposity (FM/FFM). Birth length was a positive predictor of FFMI and BMI, only among adult women. Higher relative weight gains

For peer review only

in infancy, childhood, and adolescence were associated with higher FFMI, FMI, FM/FFM ratio, BMI and waist circumference in adult life. Associations strengthened with age, thus, effect sizes were the strongest in childhood and adolescence, almost twice the magnitude of effect sizes in infancy, and four to five times the magnitude of birth estimates. Our findings also showed that a higher conditional height in infancy was a significant but weak predictor of higher FMI and BMI among adult men and women, and higher waist circumference among women. A higher conditional height in childhood was positively but weakly associated with women's FMI, FM/FFM ratio, and waist circumference.

This updated analysis confirms previous results from COHORTS[13, 14] and similar site-specific studies[28, 33, 40] at showing that birthweight is a predictor of higher adult FFMI and FMI. Thus, our findings showed the independent and long-lasting role of prenatal growth (weight gain mainly) on body composition and size in young and middle-age adulthood. There are differences in effect sizes when comparing our estimates of FMI and FFMI with previous COHORTS' analysis because we adjusted by adult height, used a different approach to combine site-specific estimates, participants were older, and possibly due to the decreased association of birthweight with adult measures across time. The positive and significant associations between prenatal and postnatal relative weight gains with all outcomes in young and mid-adulthood suggest that relative weight is a predictor of overall body size than body composition. A recent review of literature highlighted a similar conclusion in relation to rapid weight gain in infancy (0-2 years of age) with body size in childhood, adolescence, and adulthood.[7] We expand on that conclusion by showing that excessive relative weight gain beyond 2 years of age is also a predictor of overall size in young and middle-age adulthood. Additionally, we infer that a higher FFMI reflects the accretion in FFM as a body response (expansion of bone and muscle mass) to support higher weight,[41, 42] since on average COHORTS participants had overweight or obesity in adulthood. This inference is also supported by the finding that conditional relative weight in infancy, childhood and adolescence were associated with a greater FM/FFM ratio, indicating that the increments in FM were faster than the

For peer review only

increments in FMM. Thus, higher weight gains during these age intervals might be associated with a greater risk of adiposity in adult life.

Compared to previous combined analysis of COHORTs, we examined for the first time the interval from childhood to adolescence. Our findings showed that relative weight gain in adolescence was associated with higher adult adiposity (FMI, FM/FFM ratio, and waist circumference) in consistency with earlier studies in LMICs.[13, 14, 28] Studies in HIC, like an analysis of the Fels Longitudinal Study showed similar trends. This study found that timing and velocity of BMI in childhood, adolescence, and post-adolescence were predictors of higher BMI and adiposity in adulthood (ages 35-45), being adolescence and post-adolescence the strongest predictors of adult adiposity.[43] Whereas we observed similar effect sizes in childhood and adolescence suggesting that both periods are equally important on predicting the risk of higher adiposity in adult life. In a deeper understanding of the predicting role of growth on adult adiposity, recent analyses of Brazil 1982[44] and South Africa[28] cohorts found that conditional relative weight gains across different stages of the life course were significant predictors of both visceral and subcutaneous fat at ages 30 and 22, respectively.[44]

A combination of biological, environmental, behavioral, and sociodemographic factors is associated with weight gain and adiposity in childhood and adolescence that may not only persist later in life but might undermine the positive role of a healthy birthweight and linear growth early in life. For instance, the medium to strong tracking of child and adolescent BMI into adult adiposity,[8, 11] prenatal and postnatal factors,[45] reproductive characteristics of women (earlier age at menarche, short periods between age at menarche and age at first pregnancy)[46], and changes in lifestyle patterns (diets of obesogenic environments, a decline in physical activity, and an increment in sedentary activities) as consequence of the nutrition transition and transformations of the food systems, home and work environments in LMICs during the last decades.[47]

For peer review only

Fewer studies have examined the role of linear growth on later body composition and results are mixed.[7] Our results showed that linear growth in infancy was a significant but weak predictor of adiposity in adult life (FMI and BMI in men and women, and waist circumference in women). These findings are also consistent with previous reports of COHORTs[13] and South Africa birth cohort[28], but our estimates were smaller given the adjustment by adult height. Our findings showed that linear growth was not a predictor of adult FFMI, which suggests that linear growth is a predictor of adult height rather than FFM. In contrast, an earlier study in South Africa showed that linear growth across 5 age intervals (from 0-22 years of age) predicted a higher FFM in young adulthood, [28] and an analysis conducted in Guatemala before the start of obesity epidemic found that linear growth was associated with higher FFM in adolescence.[48]

Linear growth is determined by the hormonal regulation of the growth hormone (GH) secreted by the pituitary and the insulin-like growth factor 1 (IGF-1), an intermediary hormone between the GH and target tissues.[49] Based on human models, growth during the first year after birth is described as an extension of fetal growth,[50] and linear size in infancy is influenced prominently by IGF-1 that is regulated mainly by nutritional factors than by the GH.[51, 52] Thus, IGF-1 might be the hormonal link underlying the association between faster linear growth in infancy and adult adiposity given its metabolic functions (anabolism) and role in promoting linear growth.[49] Studies of British cohorts have shown that infants (0-2 years) with a faster postnatal growth (catch-up growth) in weight or height, had higher levels of IGF-1 at age 5 years, independent of birth levels.[53] IGF-1 levels in childhood were positively correlated with weight, height, and body composition in childhood.[53] Besides, children who are taller tend to be heavier, and rapid weight gains and catch-up growth in postnatal years have been associated with higher adiposity and risk of obesity in childhood and adult life.[7] In turn, animal models and clinical trials have demonstrated that excess weight causes a downregulation of the GH/IGF-1 axis leading to higher adiposity in adulthood.[54]

For peer review only

Thus, increased growth in infancy, including a faster linear growth, might induce long-lasting metabolic programing of the GH/IGF-1 axis and a subsequent increased risk of adiposity. However, the evidence on the role of linear growth on obesity risk and higher adiposity later in life is limited and inconclusive.[7] For example, a recent study of a British cohort showed that infant linear growth (ages 0-3 months) modestly improved prediction models of childhood adiposity, but the estimates were small and with a negative trend.[55] While, our findings showed that faster linear growth in infancy (0-24 months) has a weak but significant association with adult adiposity, showing a positive trend, in contrast to findings in children.

Overall, men and women showed similar trends, larger weight gain across the life course was associated with larger body size and adiposity in adult life. However, among women, estimates for size in adolescence tended to be stronger than those for childhood, in contrast to the men, in whom childhood estimated tended to be stronger. Regarding conditional height, a faster liner growth in infancy was positively but weakly associated with higher adult adiposity among both men and women, and faster linear growth in childhood was positively associated with higher adiposity among adult women but not in men.

Sex-based dimorphism in human growth patterns, body size and composition since the prenatal period, with pronounced differences in certain age intervals is well recognized.[56] In infancy, growth patterns are similar among boys and girls, a period characterized by a deceleration of linear growth and fat accretion after the first year of birth.[57] However, in childhood, after the adiposity rebound (around age 6), there is acceleration in linear growth and weight gain, accretion that is mainly due to gains in lean mass among boys and gains in fat mass among girls.[57] In adolescence, women reach puberty earlier and growth stops at a younger age compared to men. Thus, men have a longer prepubertal period for growth

For peer review only

and in adolescence they experience a greater accretion in lean mass than fat mass, whereas women weight gain is characterized by a greater accretion in fat mass and their growth velocity is slower than men.[50, 57] These sex differences remain until adult life, where men and women on average have different body stature, composition, shape and fat distribution. These changes and sex-differences in body size and composition are the result of hormonal regulations, mainly by the action of the GH/IGF-1 axis in infancy and childhood, plus the effect of steroid hormones in puberty.[50, 57] Thus, sex dimorphisms in growth patterns and body composition might explain the differences in the effect sizes and trends among men and women that we observed. Public health recommendations and interventions should pay special attention to these differences in growth and body composition among boys and girls mainly in childhood and adolescence, to prevent the excess of adiposity and high prevalence of obesity in adult life (higher among women), since early stages of life.

Some limitations of our analysis include: 1) attrition as one source of selection bias given the long-lasting follow-up of these birth cohorts; 2) residual confounding since we could not control for covariates such as diet, reproductive and life style factors; 3) missing data of covariates (ranges between 0.1-41%), for which we assumed MAR and applied multiple imputation techniques; and 4) as expected there was substantial between-study heterogeneity, mostly in associations of conditional relative weight in childhood and adolescence. Some reasons that might explain the observed heterogeneity, beyond the baseline differences in places and contexts, include: a) the age intervals definition was guided by data availability, as not all children were measured at the same intervals; b) differences in sample sizes across study sites given that we had to restrict the analysis to participants with complete anthropometric data in the four age intervals to be able to estimate conditional growth measures; c) differences in the techniques and instruments to measure body composition which might be a source of information bias (non-differential misclassification). In this regard, our research question intended to study the absolute increase in body composition not the relative estimate, and we assumed that all techniques, independent of the method and units used, ranked people in the same way. Additionally, we transformed all body

For peer review only

composition measurements into SD units in each cohort and estimated standardized betas to be able to compare estimates between sites. To minimize the previous limitations, we used the random meta-analysis approach that not only considers the underlying heterogeneity when pooling estimates but also allowed us to control for differences in sample sizes across study sites.

Among the strengths of our analyses, we highlight the prospective and long follow-up of these six birth cohorts in LMICs. We used large sample sizes (except Guatemala), and analyzed two compartments of body composition (beyond BMI) that were measured using gold standards and/or validated techniques. We studied the independent association of linear growth and weight gain with body composition through the use of conditional growth measures, which allowed us to break the high correlation between weight and height and to study the independent role of linear growth and relative weight gain in specific age intervals. We selected the best model at each study site, fully adjusted by multiple confounders, and applied a random effects meta-analysis that by default considers the heterogeneity between study sites.

We are cautious with the generalizability of our results since we used selected birth cohorts, the study design of cohorts is not intended to be representative of countries were information was collected, and there are differences (social, demographic, economic, political, cultural) in contexts and periods in which each cohort started. However, the consistency in magnitude and direction of effect sizes across study-sites (six birth cohorts from five different cities around the world), indicates that our findings are robust and suggests that the long-term role of growth on body composition might apply to individuals from other LMICs with similar characteristics.

In summary, our findings suggest that: 1) growth in the first 1,000 days of life has a small and long-lasting association with adult body size and composition; 2) childhood and adolescence exhibit the strongest associations between relative weight gain with body size and composition in adulthood; and 3) a faster linear growth in infancy (girls and boys) and childhood (girls) predicts increased

For peer review only

adiposity in adult life. Historical efforts to reduce growth faltering in LMICs have been successful, with little evidence to date of increases in BMI-for age.[30, 58, 59] However, once nutritional and other environmental constraints on growth are removed, further nutritional inputs beyond individual requirements are likely to increase adiposity. Thus, public health interventions might focus on promoting adequate linear growth in early life and a healthy weight gain throughout the life course.

Acknowledgments We thank to all COHORTS participants and study teams. In Guatemala, we acknowledge Paul Melgar, the late field director of the INCAP Nutrition Trial and Longitudinal Study.

Contributors NEP and ADS conceived the idea. NEP, LSA, RM, SAP, MRZ, and ADS were involved in the conceptualization of this analysis. LSA, MRZ, SKB, SAB, DBC, MFKL, BLH, NPL, MM, AMBM, SAN, LHN, LMR, HSS, FCW, ADS, and COHORTS group led and/or participated in data collection. NEP drafted the analysis plan, conducted the data management and statistical analysis, wrote the first draft of the manuscript, and incorporated edits along the review process. All coauthors reviewed and provided insightful comments to the analysis and manuscript drafts. All authors approved the final report.

Funding: The Bill and Melinda Gates Foundation (OPP1164115) funded the most recent wave of data collection in Guatemala, Philippines, and South Africa, and data management and analysis in Brazil. Data collection in Brazil was funded by the Wellcome Trust (086974/Z/08/Z). The New Delhi Birth Cohort has also received funding from the Indian Council of Medical Research (50/1-3/TF/05-NCD-II; 3/1/2/2/15-RCH; 5/10/FR/10/2019-RCH; 5/4/8-7/2019-NCD-II), the Department of Biotechnology (BT/PR3874/MED/97/1/2011; BT/PR5317/FNS/20/552/2012), the United States National Center for Health Statistics (PL-480, RESEARCH PROJECT 0-1-658-2), and the British Heart Foundation (UKPG/05/046). South Africa (Birth to Thirty) funders are the South African Medical Research Council, Wellcome Trust (UK, 077210/Z/05/Z; 092097/Z/10/Z), University of the Witwatersrand and the DSI-NRF Centre of Excellence in Human Development. NEP was partially supported by a Fulbright-Colciencias fellowship.

For peer review only

Competing interests None declared

Patient consent for publication Not applicable

Ethics approval and consent to participate All cohorts obtained ethics approvals for data collection from the corresponding Institutional Review Boards at each study site (Brazil, Federal University of Pelotas, reference number 1.250.366; Guatemala, Institute of Nutrition for Central America and Panama, reference number CIE-REV-072-2017; India, Sitaram Bhartia Institute of Science and Research, reference numbers SBISR/2012/002, SBISR/RES1/3/2012, SBISR/IEC/2014/001, IEC/SBISR/2015/1, and FL/SBISR/IEC/2019–01; Philippines, Research Ethics Committee at University of San Carlos, reference number 006/2018-01-borja; South Africa, Human Research Ethics Committee at University of Witswatersrand, reference number M180225). Parents or guardians of participants granted their written or verbal consent to participate at baseline and in childhood/adolescence follow-ups, while in adulthood participants gave their own consent. The present analysis was approved by the Institutional Review Board of Emory University (IRB number 95960).

Provenance and peer review Not commissioned; externally peer reviewed.

Data availability statement COHORTS data are not freely available due to privacy considerations. Data will be available upon reasonable request to the principal investigators at each study site.

For peer review only

References

1. (NCD-RisC) NRFC. Worldwide trends in diabetes since 1980: a pooled analysis of 751 population-based studies with 4.4 million participants. *Lancet* 2016;387(10027):1513-30. doi: 10.1016/s0140-6736(16)00618-8 [published Online First: 2016/04/12]
2. (NCD-RisC) NRFC. Worldwide trends in blood pressure from 1975 to 2015: a pooled analysis of 1479 population-based measurement studies with 19·1 million participants. *Lancet* 2017;389(10064):37-55. doi: 10.1016/s0140-6736(16)31919-5 [published Online First: 2016/11/20]
3. (NCD-RisC) NRFC. Trends in adult body-mass index in 200 countries from 1975 to 2014: a pooled analysis of 1698 population-based measurement studies with 19·2 million participants. *Lancet* 2016;387(10026):1377-96. doi: 10.1016/s0140-6736(16)30054-x [published Online First: 2016/04/27]
4. Ezzati M, Pearson-Stuttard J, Bennett JE, et al. Acting on non-communicable diseases in low- and middle-income tropical countries. *Nature* 2018;559(7715):507-16. doi: 10.1038/s41586-018-0306-9 [published Online First: 2018/07/27]
5. Barker DJ. The developmental origins of well-being. *Philos Trans R Soc Lond B Biol Sci* 2004;359(1449):1359-66. doi: 10.1098/rstb.2004.1518 [published Online First: 2004/09/07]
6. Wells JC, Chomtho S, Fewtrell MS. Programming of body composition by early growth and nutrition. *Proc Nutr Soc* 2007;66(3):423-34. doi: 10.1017/s0029665107005691 [published Online First: 2007/07/20]
7. Woo JG. Infant Growth and Long-term Cardiometabolic Health: a Review of Recent Findings. *Curr Nutr Rep* 2019;8(1):29-41. doi: 10.1007/s13668-019-0259-0 [published Online First: 2019/02/08]
8. Umer A, Kelley GA, Cottrell LE, et al. Childhood obesity and adult cardiovascular disease risk factors: a systematic review with meta-analysis. *BMC Public Health* 2017;17(1):683. doi: 10.1186/s12889-017-4691-z [published Online First: 20170829]
9. Lloyd LJ, Langley-Evans SC, McMullen S. Childhood obesity and adult cardiovascular disease risk: a systematic review. *International journal of obesity (2005)* 2010;34(1):18-28. doi: 10.1038/ijo.2009.61 [published Online First: 20090512]
10. Lloyd LJ, Langley-Evans SC, McMullen S. Childhood obesity and risk of the adult metabolic syndrome: a systematic review. *International journal of obesity (2005)* 2012;36(1):1-11.
11. Singh AS, Mulder C, Twisk JW, et al. Tracking of childhood overweight into adulthood: a systematic review of the literature. *Obes Rev* 2008;9(5):474-88. doi: 10.1111/j.1467-789X.2008.00475.x [published Online First: 20080305]

For peer review only

12. Ward LC. Human body composition: yesterday, today, and tomorrow. *Eur J Clin Nutr* 2018;72(9):1201-07. doi: 10.1038/s41430-018-0210-2 [published Online First: 2018/09/07]

13. Adair LS, Fall CH, Osmond C, et al. Associations of Linear Growth and Relative Weight Gain During Early Life With Adult Health and Human Capital in Countries of Low and Middle Income: Findings From Five Birth Cohort Studies. *Lancet (London, England)* 2013;382(9891) doi: 10.1016/S0140-6736(13)60103-8

14. Kuzawa CW, Hallal PC, Adair L, et al. Birth weight, postnatal weight gain, and adult body composition in five low and middle income countries. *Am J Hum Biol* 2012;24(1):5-13. doi: 10.1002/ajhb.21227 [published Online First: 2011/11/28]

15. Weng SF, Redsell SA, Swift JA, et al. Systematic review and meta-analyses of risk factors for childhood overweight identifiable during infancy. *Arch Dis Child* 2012;97(12):1019-26. doi: 10.1136/archdischild-2012-302263 [published Online First: 20121029]

16. Rallis D, Balomenou F, Tzoufi M, et al. A systematic review indicates an association between birth weight and body fat in childhood. *Acta Paediatr* 2021;110(7):2023-39. doi: 10.1111/apa.15834 [published Online First: 20210315]

17. Yang Z, Huffman SL. Nutrition in pregnancy and early childhood and associations with obesity in developing countries. *Matern Child Nutr* 2013;9 Suppl 1(Suppl 1):105-19. doi: 10.1111/mcn.12010 [published Online First: 2012/11/28]

18. Richter LM, Victora CG, Hallal PC, et al. Cohort profile: the consortium of health-orientated research in transitioning societies. *Int J Epidemiol* 2012;41(3):621-6. doi: 10.1093/ije/dyq251 [published Online First: 2011/01/13]

19. Horta BL, Gigante DP, Gonçalves H, et al. Cohort Profile Update: The 1982 Pelotas (Brazil) Birth Cohort Study. *Int J Epidemiol* 2015;44(2):441, 41a-41e. doi: 10.1093/ije/dyv017 [published Online First: 2015/03/04]

20. Victora CG, Barros FC. Cohort profile: the 1982 Pelotas (Brazil) birth cohort study. *Int J Epidemiol* 2006;35(2):237-42. doi: 10.1093/ije/dyi290 [published Online First: 2005/12/24]

21. Victora CG, Hallal PC, Araújo CL, et al. Cohort Profile: The 1993 Pelotas (Brazil) Birth Cohort Study. *International journal of epidemiology* 2008;37(4) doi: 10.1093/ije/dym177

22. Stein AD, Melgar P, Hoddinott J, et al. Cohort Profile: the Institute of Nutrition of Central America and Panama (INCAP) Nutrition Trial Cohort Study. *Int J Epidemiol* 2008;37(4):716-20. doi: 10.1093/ije/dyn028 [published Online First: 2008/02/21]

23. Bhargava SK, Sachdev HS, Fall CH, et al. Relation of serial changes in childhood body-mass index to impaired glucose tolerance in young adulthood. *N Engl J Med* 2004;350(9):865-75.

For peer review only

24. Adair LS, Popkin BM, Akin JS, et al. Cohort profile: the Cebu longitudinal health and nutrition survey. *Int J Epidemiol* 2011;40(3):619-25. doi: 10.1093/ije/dyq085 [published Online First: 2010/05/29]

25. Richter L, Norris S, Pettifor J, et al. Cohort Profile: Mandela's children: the 1990 Birth to Twenty study in South Africa. *Int J Epidemiol* 2007;36(3):504-11. doi: 10.1093/ije/dym016 [published Online First: 2007/03/16]

26. Mei Z, Grummer-Strawn LM. Standard deviation of anthropometric Z-scores as a data quality assessment tool using the 2006 WHO growth standards: a cross country analysis. *Bull World Health Organ* 2007;85(6):441-8. doi: 10.2471/blt.06.034421

27. Jesson J, Schomaker M, Malasteste K, et al. Stunting and growth velocity of adolescents with perinatally acquired HIV: differential evolution for males and females. A multiregional analysis from the IeDEA global paediatric collaboration. *J Int AIDS Soc* 2019;22(11):e25412. doi: 10.1002/jia2.25412

28. Prioreschi A, Munthali RJ, Kagura J, et al. The associations between adult body composition and abdominal adiposity outcomes, and relative weight gain and linear growth from birth to age 22 in the Birth to Twenty Plus cohort, South Africa. *PLoS One* 2018;13(1):e0190483. doi: 10.1371/journal.pone.0190483 [published Online First: 20180116]

29. Gonçalves H, Wehrmeister FC, Assunção MCF, et al. Cohort Profile Update: The 1993 Pelotas (Brazil) Birth Cohort follow-up at 22 years. *Int J Epidemiol* 2018;47(5):1389-90e. doi: 10.1093/ije/dyx249 [published Online First: 2017/12/15]

30. Kroker-Lobos MF, Ramirez-Zea M, Stein AD. Overweight and Obesity, Cardiometabolic Health, and Body Composition: Findings From the Follow-Up Studies of the INCAP Longitudinal Study. *Food and Nutrition Bulletin* 2020;41(1_suppl):S59-S68. doi: 10.1177/0379572120903222

31. Menezes AMB, Oliveira PD, Wehrmeister FC, et al. Associations between growth from birth to 18 years, intelligence, and schooling in a Brazilian cohort. *The American journal of clinical nutrition* 2020 doi: 10.1093/ajcn/nqaa047 [published Online First: 2020/04/03]

32. Li H, Stein AD, Barnhart HX, et al. Associations between prenatal and postnatal growth and adult body size and composition. *The American journal of clinical nutrition* 2003;77(6):1498-505. doi: 10.1093/ajcn/77.6.1498 [published Online First: 2003/06/07]

33. Sachdev HS, Fall CH, Osmond C, et al. Anthropometric indicators of body composition in young adults: relation to size at birth and serial measurements of body mass index in childhood in the New Delhi birth cohort. *The American journal of clinical nutrition* 2005;82(2):456-66. doi: 10.1093/ajcn.82.2.456 [published Online First: 2005/08/10]

34. Adair LS. Size at birth and growth trajectories to young adulthood. *Am J Hum Biol* 2007;19(3):327-37. doi: 10.1002/ajhb.20587

For peer review only

35. WHO. The WHO Child Growth Standards: World Health Organization; 2016 [updated 2016-06-08 09:11:46. Available from: https://www.who.int/childgrowth/en/.

36. Li C, Ford ES, Zhao G, et al. Estimates of body composition with dual-energy X-ray absorptiometry in adults. *The American journal of clinical nutrition* 2009;90(6):1457-65. doi: 10.3945/ajcn.2009.28141 [published Online First: 2009/10/09]

37. Harrer M, Cuijpers P, Furukawa TA, et al. Doing Meta-Analysis With R: A Hands-On Guide.

38. Judd SE, Ramirez-Zea M, Stein AD. Relation of ratio indices of anthropometric measures to obesity in a stunted population. *Am J Hum Biol* 2008;20(4):446-50. doi: 10.1002/ajhb.20762

39. Wells JC, Victora CG. Indices of whole-body and central adiposity for evaluating the metabolic load of obesity. *International journal of obesity (2005)* 2005;29(5):483-9. doi: 10.1038/sj.ijo.0802899

40. Corvalán C, Gregory CO, Ramirez-Zea M, et al. Size at birth, infant, early and later childhood growth and adult body composition: a prospective study in a stunted population. *Int J Epidemiol* 2007;36(3):550-7. doi: 10.1093/ije/dym010 [published Online First: 2007/03/23]

41. Savvidis C, Tournis S, Dede AD. Obesity and bone metabolism. *Hormones (Athens)* 2018;17(2):205-17. doi: 10.1007/s42000-018-0018-4 [published Online First: 20180424]

42. Tomlinson DJ, Erskine RM, Morse CI, et al. The impact of obesity on skeletal muscle strength and structure through adolescence to old age. *Biogerontology* 2016;17(3):467-83. doi: 10.1007/s10522-015-9626-4 [published Online First: 20151214]

43. Guo SS, Huang C, Maynard LM, et al. Body mass index during childhood, adolescence and young adulthood in relation to adult overweight and adiposity: the Fels Longitudinal Study. *International journal of obesity and related metabolic disorders : journal of the International Association for the Study of Obesity* 2000;24(12):1628-35. doi: 10.1038/sj.ijo.0801461

44. Araújo de França GV, De Lucia Rolfe E, Horta BL, et al. Associations of birth weight, linear growth and relative weight gain throughout life with abdominal fat depots in adulthood: the 1982 Pelotas (Brazil) birth cohort study. *International journal of obesity (2005)* 2016;40(1):14-21. doi: 10.1038/ijo.2015.192 [published Online First: 20150923]

45. Larqué E, Labayen I, Flodmark CE, et al. From conception to infancy - early risk factors for childhood obesity. *Nat Rev Endocrinol* 2019;15(8):456-78. doi: 10.1038/s41574-019-0219-1 [published Online First: 20190703]

46. Mazariegos M, Varghese JS, Kroker-Lobos MF, et al. Age at childbirth and change in BMI across the life-course: evidence from the INCAP Longitudinal Study. *BMC Pregnancy Childbirth* 2022;22(1):151. doi: 10.1186/s12884-022-04485-6 [published Online First: 20220224]

For peer review only

47. Popkin BM, Corvalan C, Grummer-Strawn LM. Dynamics of the double burden of malnutrition and the changing nutrition reality. *Lancet* 2020;395(10217):65-74. doi: 10.1016/s0140-6736(19)32497-3 [published Online First: 20191215]

48. Rivera JA, Martorell R, Ruel MT, et al. Nutritional supplementation during the preschool years influences body size and composition of Guatemalan adolescents. *The Journal of nutrition* 1995;125(4 Suppl):1068s-77s. doi: 10.1093/jn/125.suppl_4.1068S [published Online First: 1995/04/01]

49. Laron Z. Insulin-like growth factor 1 (IGF-1): a growth hormone. *Mol Pathol* 2001;54(5):311-6. doi: 10.1136/mp.54.5.311

50. Karlberg J. A biologically-oriented mathematical model (ICP) for human growth. *Acta Paediatr Scand Suppl* 1989;350:70-94. doi: 10.1111/j.1651-2227.1989.tb11199.x

51. Fliesen T, Maiter D, Gerard G, et al. Reduction of serum insulin-like growth factor-I by dietary protein restriction is age dependent. *Pediatr Res* 1989;26(5):415-9. doi: 10.1203/00006450-198911000-00010

52. Ong KK, Elmlinger M, Jones R, et al. Growth hormone binding protein levels in children are associated with birth weight, postnatal weight gain, and insulin secretion. *Metabolism: clinical and experimental* 2007;56(10):1412-7. doi: 10.1016/j.metabol.2007.06.004

53. Ong K, Kratzsch J, Kiess W, et al. Circulating IGF-I levels in childhood are related to both current body composition and early postnatal growth rate. *The Journal of clinical endocrinology and metabolism* 2002;87(3):1041-4. doi: 10.1210/jcem.87.3.8342

54. Berryman DE, Glad CA, List EO, et al. The GH/IGF-1 axis in obesity: pathophysiology and therapeutic considerations. *Nat Rev Endocrinol* 2013;9(6):346-56. doi: 10.1038/nrendo.2013.64 [published Online First: 20130409]

55. Ong KK, Cheng TS, Olga L, et al. Which infancy growth parameters are associated with later adiposity? The Cambridge Baby Growth Study. *Ann Hum Biol* 2020;47(2):142-49. doi: 10.1080/03014460.2020.1745887

56. WHO Multicentre Growth Reference Study Group. WHO Child Growth Standards based on length/height, weight and age. *Acta Paediatr Suppl* 2006;450:76-85. doi: 10.1111/j.1651-2227.2006.tb02378.x

57. Wells JC. Sexual dimorphism of body composition. *Best Pract Res Clin Endocrinol Metab* 2007;21(3):415-30. doi: 10.1016/j.beem.2007.04.007

58. Martorell R. History and Design of the INCAP Longitudinal Study (1969-1977) and Its Impact in Early Childhood. *Food Nutr Bull* 2020;41(1_suppl):S8-s22. doi: 10.1177/0379572120906062 [published Online First: 2020/06/12]

For peer review only

59. Stein AD, Wang M, Ramirez-Zea M, et al. Exposure to a nutrition supplementation intervention in early childhood and risk factors for cardiovascular disease in adulthood: evidence from Guatemala. *Am J Epidemiol* 2006;164(12):1160-70. doi: 10.1093/aje/kwj328 [published Online First: 20061003]

Figure Legend

Figure 1. Flowchart of analytic samples selection, COHORTS

For peer review only

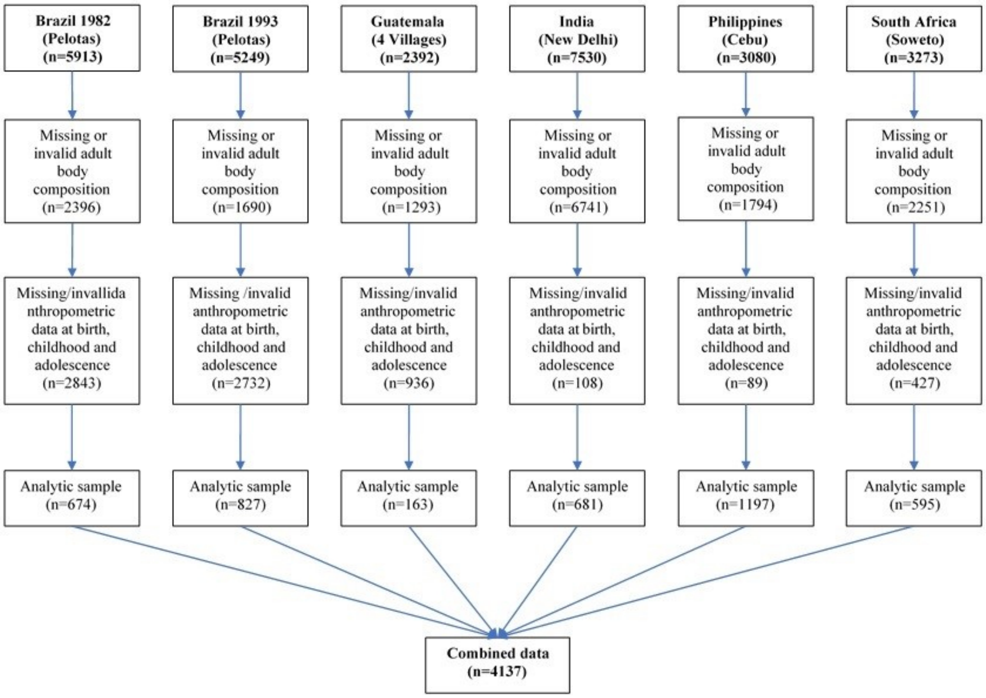

Figure 1. Flowchart of analytic samples selection, COHORTS

72x50mm (300 x 300 DPI)

Supplementary Tables

Supplementary Table 1. Data missing patterns (covariates) of analytic samples at each study site

Covariates	Brazil 1982 (n=674)	Brazil 1993 (n=827)	Guatemala (n=163)	India (n=681)	The Philippines (n=1197)	South Africa (n=595)
Sex	0	0	0	0	0	0
Gestational age	137 (20.3%)	91 (11%)	10 (6.1%)	149	8 (0.7%)	4 (0.7%)
Birth order	0	0	0	256 (37.6%)	0	0
Maternal age at first child birth	0	0	0	227 (33.3%)	0	0
Maternal height	11 (1.6%)	8 (1%)	0	246 (36.1%)	0	84 (14.1%)
Maternal schooling	1 (0.1%)	12 (1.4%)	1 (0.6%)	62	0	24 (4.0%)
Maternal marital status	0	0	0	0	0	0
Paternal age at child birth	163 (24.2%)	NA	3 (1.8%)	NA	36 (3.0%)	NA
Paternal schooling	40 (5.9%)	48 (5.8%)	3 (1.8%)	116 (17.0%)	59 (4.9%)	158 (26.5%)
Income in childhood	7 (1.0%)	6 (0.7%)	NA	222 (32.6%)	5 (0.4%)	244 (41.0%)
Wealth index in childhood	0	NA	1 (0.6%)	222 (32.6%)	0	25 (4.2%)
Age at menarche (women)	0	NA	7 (4.3%)	52 (7.6%)	1 (0.1%)	4 (0.7%)
Teenage childbearing	12 (1.8%)	24 (2.9%)	24 (14.7%)	280 (41.1%)	253 (21.1%)	NA
Skin color Brazil	0	0	NA	NA	NA	NA

NA: not available variable

For peer review only

Supplementary Table 2. Characteristics of participants excluded (missing anthropometric and body composition data) and included in the analyses at each study site

	Brazil 82		Brazil 93		Guatemala		India		The Philippines		South Africa	
	Excluded (N=5239)	Analytic sample (N=674)	Excluded (N=4421)	Analytic sample (N=827)	Excluded (N=2229)	Analytic sample (N=163)	Excluded (N=6849)	Analytic sample (N=681)	Excluded (N=1883)	Analytic sample (N=1197)	Excluded (N=2678)	Analytic sample (N=595)
Birth Variables												
Sex												
n	5239	674	4421	827	2229	163	6849	681	1883	1197	2678	595
Women	48.6%	49.0%	49.6%	54.7%*	47.8%	58.9%*	48.9%	37.4%*	48.2%	45.2%	51.3%	51.8%
Gestational age (weeks)												
n	4063	537	3936	736	698	153	847	532	1859	1189	2580	591
Mean (SD)	39.3 (1.9)	39.5 (1.8)	39.5 (2.3)	39.3 (2.5)	39.3 (2.9)	39.2 (3.4)	38.9 (2.7)	38.9 (2.4)	38.7 (2.2)	38.8 (2.1)	38.2 (1.9)	37.9* (2.0)
Birth length (cm)												
n	NA	NA	4335	827	689	163	5964	681	1853	1197	NA	NA
Mean (SD)	NA	NA	48.9 (2.3)	48.1* (2.6)	50.0 (2.3)	49.0*(2.4)	48.2 (2.3)	48.4 (2.1)	49.1 (2.1)	49.1 (2.0)	NA	NA
Birth weight (kg)												
n	5131	674	4405	827	810	163	6128	681	1832	1197	2672	595
Mean (SD)	3.2 (0.6)	3.3* (0.6)	3.2 (0.5)	3.0* (0.6)	3.1 (0.5)	3.0 (0.5)	2.8 (0.4)	2.8 (0.4)	3.0 (0.5)	3.0 (0.4)	3.1 (0.5)	3.1 (0.5)
Childhood variables												
HAZ in infancy												
n	4162	674	536	827	922	163	4661	681	1307	1197	1210	595
Mean (SD)	-0.7 (1.2)	-0.5*(1.2)	-0.3 (1.6)	-0.3 (1.3)	-2.9 (1.2)	-2.9 (1.1)	-1.9 (1.2)	-2.0 (1.1)	-2.4 (1.2)	-2.4 (1.1)	-1.1 (1.2)	-1.2 (1.0)
HAZ in childhood												
n	3978	674	440	827	951	163	4151	681	1064	1197	1209	595
Mean (SD)	-0.7 (1.1)	-0.6 (1.1)	-0.3 (1.3)	-0.3 (1.1)	-2.4 (1.0)	-2.3 (0.9)	-1.9 (1.1)	-2.0 (1.0)	-2.0 (1.0)	-2.1 (0.9)	-0.9 (0.9)	-1.0 (0.9)
HAZ in adolescence												
n	398	674	3172	827	724	163	2384	681	891	1197	1328	595
Mean (SD)	-0.3 (1.0)	-0.2 (1.0)	-0.1 (1.0)	-0.3* (1.0)	-2.0 (0.9)	-1.9 (0.8)	-1.1 (1.0)	-1.2 (1.0)	-1.8 (0.9)	-1.9* (0.8)	-0.6 (1.0)	-0.6 (0.9)
WAZ in infancy												
n	4163	674	534	827	922	163	4623	681	1307	1197	1596	595
Mean (SD)	0.1 (1.1)	0.2 (1.1)	0.2 (1.4)	0.3 (1.1)	-1.7 (1.0)	-1.7(1.0)	-1.4 (1.1)	-1.5 (1.1)	-1.7 (1.1)	-1.7 (0.9)	-0.4 (1.0)	-0.5 (1.0)
WAZ in childhood												
n	3978	674	444	827	950	163	4127	681	1064	1197	1604	595
Mean (SD)	-0.0 (1.1)	0.1 (1.0)	0.5 (2.9)	0.1 (1.2)*	-1.4 (0.8)	-1.4(0.8)	-1.3 (1.0)	-1.4 (0.9)	-1.8 (1.1)	-2.0 (0.9)*	-0.4 (0.9)	-0.5 (0.9)*
BAZ in adolescence												
n	398	674	3172	827	723	163	2384	681	891	1197	1323	595
Mean (SD)	0.3 (1.1)	0.3 (1.1)	0.4 (1.2)	0.3* (1.1)	-0.5 (0.8)	-0.3 (0.9)	-0.8 (1.2)	-1.0* (1.2)	-0.8 (1.1)	-0.9 (1.0)	-0.1 (1.3)	-0.1 (1.2)

For peer review only

Adult variables												
Age (years)												
n	2989	667	2986	827	1115	153	149	681	129	1197	913	484
Mean (SD)	30.2 (0.3)	30.2 (0.3)	22.6 (0.3)	22.6 (0.3)	47.7 (4.4)	45.5*(1.1)	46.1 (1.2)	46.1 (1.1)	34.5 (0.5)	34.4 (0.5)	22.0 (0.4)	22.0 (0.4)
Height (cm)												
n	2933	674	2765	827	982	163	140	681	129	1197	921	595
Mean (SD)	167.5(9.2)	168.6*(9.3)	167.8(9.4)	166.4*(10.0)	156.3(8.3)	157.3(8.2)	164.7(9.1)	164.1(9.8)	152.6(26.1)	157.4*(8.1)	165.7(8.9)	165.4(8.6)
Weight (kg)												
n	2877	674	2764	827	982	163	136	681	126	1197	911	595
Mean (SD)	75.6(17.7)	76.6(17.6)	71.9(16.8)	69.6*(16.4)	68.7(13.2)	70.2(12.9)	76.5(14.5)	76.6(15.0)	62.1(14.9)	62.1 (13.1)	64.8(14.8)	63.8(13.3)
BMI (kg/m^2)												
n	2863	671	2735	827	982	163	135	681	107	1197	911	595
Mean (SD)	26.8 (5.5)	26.9 (5.5)	25.3 (5.3)	25.0 (5.4)	28.1 (5.1)	28.5 (5.1)	28.4 (5.5)	28.4 (4.8)	24.8 (5.0)	25.0 (4.6)	23.7 (5.7)	23.4 (5.1)
Waist circumference (cm)												
n	2893	674	2766	827	982	163	138	681	103	1197	902	595
Mean (SD)	84.7(12.6)	85.4(12.5)	80.2(11.6)	79.2 (11.5)	98.7(12.4)	99.1(11.6)	97.8(12.1)	97.3(12.2)	81.1(12.6)	81.5(11.1)	78.4(11.8)	79.2(11.8)
FMI, kg/m^2												
n	2849	674	2735	827	939	163	112	681	90	1197	452	595
Mean (SD)	8.7 (4.6)	8.8 (4.5)	7.7 (4.5)	7.5 (4.6)	10.7 (4.1)	10.7(3.9)	8.6 (3.7)	9.1 (3.6)	6.7 (3.4)	7.1 (3.5)	6.7 (4.3)	6.9 (4.2)
FFMI, kg/m^2												
n	2849	674	2735	827	941	163	112	681	90	1197	452	595
Mean (SD)	18.1 (2.7)	18.1 (2.7)	17.5 (2.6)	17.3 (2.5)	17.5 (2.4)	17.8 (2.5)	19.5 (2.6)	19.3 (2.4)	17.6 (2.2)	17.9 (2.3)	14.8 (2.1)	14.6 (2.0)

*p<0.01 based on Bonferroni-adjusted comparisons of participants excluded and included in the analytic sample at each study site

For peer review only

Supplementary Table 3. Characteristics at birth, childhood and parental of study participants, stratified by study site and sex[1]

	Brazil 1982		Brazil 1993		Guatemala		India		The Philippines		South Africa	
	Men (n=344)	Women (n=330)	Men (n=375)	Women (n=452)	Men (n=67)	Women (n=96)	Men (n=426)	Women (n=255)	Men (n=656)	Women (n=541)	Men (n=287)	Women (n=308)
**Birth variables**												
Gestational age (weeks)	39.4 (1.8)	39.5 (1.8)	39.6 (2.2)	39.7 (2.3)	39.3 (3.0)	39.1 (3.6)	38.8 (2.4)	39.1 (2.3)	38.7 (2.1)	38.8 (2.1)	38.0 (1.9)	37.8 (2.0)
Birth order (%)												
1	143 (41.6%)	120 (36.4%)	147 (37.1%)	145 (33.1%)	12 (17.9%)	14 (14.6%)	46 (17.2%)	26 (16.5%)	149 (22.7%)	120 (22.2%)	105 (36.6%)	118 (38.3%)
2	98 (28.5%)	85 (25.8%)	107 (27.2%)	130 (29.6%)	13 (19.4%)	12 (12.5%)	59 (22.1%)	37 (23.4%)	132 (20.1%)	120 (22.2%)	86 (30.0%)	93 (30.2%)
3	54 (15.7%)	64 (19.4%)	63 (15.8%)	99 (22.6%)	6 (9.0%)	15 (15.6%)	53 (19.9%)	31 (19.6%)	130 (19.8%)	103 (19.0%)	48 (16.7%)	51 (16.6%)
≥4	49 (14.2%)	61 (18.5%)	79 (20.0%)	64 (14.6%)	36 (53.7%)	55 (57.3%)	109 (40.8%)	64 (40.5%)	245 (37.3%)	198 (36.6%)	48 (16.7%)	46 (14.9%)
**Childhood variables[2]**												
HAZ in infancy	-0.6 (1.2)	-0.5 (1.2)	-0.1 (1.3)	-0.0 (1.2)	-3.1 (1.2)	-2.8 (1.0)	-2.0 (1.1)	-1.9 (1.1)	-2.5 (1.1)	-2.4 (1.1)	-1.4 (1.0)	-1.1 (1.0)
HAZ in childhood	-0.5 (1.1)	-0.6 (1.1)	-0.1 (1.2)	-0.2 (1.1)	-2.3 (0.9)	-2.4 (0.9)	-1.9 (1.0)	-2.1 (1.0)	-2.1 (0.9)	-2.1 (0.9)	-1.0 (0.9)	-0.9 (0.9)
HAZ in adolescence	-0.1 (1.0)	-0.3 (0.9)	0.5 (1.2)	0.5 (1.0)	-2.0 (0.9)	-1.9 (0.7)	-1.2 (1.1)	-1.3 (0.8)	-1.9 (0.8)	-1.9 (0.8)	-0.7 (1.0)	-0.5 (0.9)
WAZ at infancy	0.2 (1.1)	0.2 (0.1)	0.3 (1.2)	0.2 (1.1)	-1.9 (1.0)	-1.6 (1.0)	-1.5 (1.1)	-1.5 (1.0)	-1.7 (0.9)	-1.7 (1.0)	-0.7 (1.1)	-0.4 (0.9)
WAZ in childhood	0.2 (1.0)	0.0 (1.0)	0.6 (1.2)	0.4 (1.1)	-1.4 (0.7)	-1.4 (0.8)	-1.3 (0.9)	-1.5 (0.9)	-2.0 (1.0)	-1.9 (0.9)	-0.5 (0.9)	-0.5 (0.9)
BAZ in adolescence	0.3 (1.1)	0.3 (1.1)	0.3 (1.2)	0.3 (1.1)	-0.9 (0.8)	0.1 (0.7)	-1.3 (1.2)	-0.6 (1.0)	-1.0 (1.0)	-0.7 (1.0)	-0.5 (1.1)	0.2 (1.2)
**Parental variables**												
Maternal age at childbirth (years)	26.5 (6.4)	26.8 (6.5)	26.3 (6.5)	26.4 (6.3)	26.7 (7.9)	27.7 (7.2)	26.9 (5.9)	26.9 (5.5)	26.6 (6.2)	26.4 (6.0)	26.0 (6.5)	25.7 (6.4)
Maternal height (cm)	157.1(6.4)	156.7(6.2)	160.2(6.5)	160.0(6.9)	148.3(4.7)	148.7(5.1)	151.9(5.8)	151.8(5.1)	150.4(4.8)	150.2(5.0)	158.2(6.4)	158.6(6.0)
Maternal schooling (years)	6.7 (4.1)	6.8 (4.3)	7.0 (3.5)	6.7 (3.4)	1.3 (1.5)	1.3 (1.5)	5.7 (4.5)	5.6 (4.4)	6.9 (3.3)	6.7 (3.1)	9.6 (2.5)	9.7 (2.7)
Paternal age (years)	29.8 (7.0)	30.3 (7.0)	NA	NA	32.7 (9.4)	32.9 (8.4)	NA	NA	29.1 (6.8)	28.9 (6.8)	NA	NA
Paternal schooling (years)	6.9 (4.3)	7.1 (4.0)	6.8 (3.6)	6.8 (3.4)	2.0 (2.0)	1.8 (2.0)	10.4 (5.0)	11.4 (4.7)	7.0 (3.5)	6.7 (3.2)	10.6 (2.2)	10.7 (2.6)

[1] Values are means (SD) or n (percentages). Sample sizes (n) refer to participants with non-missing data for outcomes (adult body composition), and non-missing data for exposures (growth measures). NA, not available data.[2] HAZ, height-for-age Z-score; WAZ, weight-for-age Z-score; BAZ, BMI-for-age Z-score; BMI, body mass index. HAZ and WAZ measures in infancy were measured at 24 months of age in all sites except Brazil 1993, where children were measured at 12 months of age. Childhood corresponds to 48 months for all study sites but Philippines, where anthropometric measures were obtained at 102 months of age. Measures of adolescence were obtained at 15 years of age (180 months) in all study sites.

For peer review only

Supplementary Table 4. Adjusted associations between weight at birth, conditional growth (height and relative weight) in infancy, childhood, and adolescence with adult fat mass index (FMI, SD units), stratified by study site and sex[1]

	Brazil 1982		Brazil 1993		Guatemala		India		The Philippines		South Africa	
	Men (n = 344)	Women (n = 330)	Men (n = 375)	Women (n = 452)	Men (n = 67)	Women (n = 96)	Men (n = 426)	Women (n = 255)	Men (n = 656)	Women (n = 541)	Men (n = 287)	Women (n = 308)
**Conditional growth, z-scores[2]**												
Birth weight	0.04 (-0.03,0.11)	-0.02 (-0.1,0.07)	0.02 (-0.05,0.09)	0.11* (0.04,0.17)	0.10 (-0.18,0.38)	0.21 (-0.03,0.44)	0.15* (0.05,0.25)	0.18* (0.04,0.32)	0.10* (0.03,0.18)	0.12* (0.04,0.20)	-0.01 (-0.11,0.09)	0.02 (-0.07,0.11)
Conditional relative weight in infancy	0.18* (0.08,0.28)	0.32* (0.17,0.46)	0.22* (0.12,0.32)	0.23* (0.14,0.32)	0.47 (-0.09,1.04)	-0.06 (-0.52,0.41)	0.20* (0.06,0.34)	0.25* (0.07,0.44)	0.25* (0.15,0.36)	0.33* (0.21,0.45)	0.22* (0.12,0.31)	0.24* (0.12,0.36)
Conditional relative weight in childhood	0.39* (0.24,0.55)	0.49* (0.29,0.68)	0.32* (0.22,0.42)	0.50* (0.40,0.61)	0.02 (-0.83,0.87)	0.43 (-0.18,1.05)	0.36* (0.15,0.56)	0.04 (-0.22,0.3)	0.59* (0.46,0.72)	0.72* (0.57,0.88)	0.28* (0.12,0.45)	0.29* (0.11,0.46)
Conditional relative weight in adolescence	0.43* (0.35,0.51)	0.61* (0.52,0.71)	0.38* (0.30,0.45)	0.56* (0.45,0.66)	0.19 (-0.32,0.71)	0.53* (0.13,0.94)	0.28* (0.19,0.37)	0.50* (0.35,0.66)	0.38* (0.29,0.47)	0.42* (0.32,0.52)	0.56* (0.47,0.66)	0.58* (0.49,0.67)
Conditional height in infancy	0.01 (-0.06,0.09)	0.17* (0.07,0.27)	0.05* (0.00,0.11)	0.06 (-0.01,0.13)	0.27 (-0.06,0.61)	0.10 (-0.19,0.39)	0.09 (0.00,0.19)	0.12 (-0.03,0.28)	0.12* (0.05,0.19)	0.14* (0.06,0.21)	0.06 (-0.03,0.16)	0.10* (0.01,0.19)
Conditional height in childhood	-0.07 (-0.20,0.07)	0.00 (-0.17,0.17)	0.13* (0.05,0.21)	0.14* (0.03,0.24)	-0.12 (-0.59,0.35)	0.00 (-0.62,0.62)	0.10 (-0.08,0.27)	0.12 (-0.12,0.36)	0.13* (0.02,0.24)	0.25* (0.13,0.36)	0.09 (-0.09,0.26)	0.16 (0,0.33)
Conditional height in adolescence	0.07 (-0.04,0.17)	0.22* (0.07,0.37)	0.08 (-0.01,0.16)	0.03 (-0.09,0.15)	0.38 (-0.1,0.86)	-0.06 (-0.65,0.53)	0.00 (-0.12,0.12)	0.03 (-0.25,0.31)	0.00 (-0.15,0.14)	-0.10 (-0.28,0.08)	-0.1 (-0.25,0.04)	-0.08 (-0.23,0.08)

[1] Values are standardized linear regression coefficients (βs and 95% CIs). In adjusted analyses we controlled for birth characteristics (gestational age, birth order), maternal characteristics (height, age at childbirth, schooling, marital status), paternal characteristics (age, schooling), income and/or wealth index of child's household at birth, and age in adulthood. In women' models, we adjusted for age of menarche and teenage childbearing. Additionally, we controlled for maternal skin color in both Brazilian cohorts and adjusted for birth year and intervention group (village fixed effects) in Guatemala analysis. * P value < 0.05; SD = Standard Deviation

[2] We used birth weight as the anchor in all conditional models given that this measure was available across all study sites

For peer review only

Supplementary Table 5. Adjusted associations between weight at birth, conditional growth (height and relative weight) in infancy, childhood, and adolescence with adult fat-free mass index (FFMI, SD units), stratified by study site and sex[1]

	Brazil 1982		Brazil 1993		Guatemala		India		The Philippines		South Africa	
	Men (n = 344)	Women (n = 330)	Men (n = 375)	Women (n = 452)	Men (n = 67)	Women (n = 96)	Men (n = 426)	Women (n = 255)	Men (n = 656)	Women (n = 541)	Men (n = 287)	Women (n = 308)
**Conditional growth, z-scores[2]**												
Birth weight	0.11** (0.04,0.17)	0.07* (0.01,0.12)	0.05* (0.00,0.10)	0.10* (0.05,0.14)	-0.06 (-0.31,0.19)	0.41* (0.21,0.62)	0.12* (0.02,0.21)	0.12 (-0.01,0.25)	0.13* (0.06,0.21)	0.10* (0.03,0.18)	0.20* (0.10,0.30)	0.15* (0.06,0.23)
Conditional relative weight in infancy	0.3*** (0.2,0.39)	0.2*** (0.11,0.29)	0.30* (0.22,0.37)	0.29* (0.22,0.36)	0.45 (-0.05,0.95)	0.23 (-0.18,0.64)	0.34* (0.21,0.46)	0.40* (0.23,0.57)	0.31* (0.21,0.41)	0.37* (0.26,0.49)	0.18* (0.09,0.28)	0.21* (0.09,0.32)
Conditional relative weight in childhood	0.41*** (0.27,0.55)	0.4*** (0.27,0.52)	0.43* (0.33,0.52)	0.41* (0.33,0.49)	0.33 (-0.42,1.08)	0.20 (-0.34,0.74)	0.46* (0.26,0.65)	0.37* (0.13,0.61)	0.71* (0.59,0.83)	0.71* (0.56,0.86)	0.31* (0.15,0.46)	0.41* (0.24,0.58)
Conditional relative weight in adolescence	0.39*** (0.31,0.46)	0.39*** (0.33,0.45)	0.35* (0.28,0.41)	0.38* (0.32,0.44)	0.58* (0.12,1.03)	0.45* (0.10,0.81)	0.36* (0.28,0.44)	0.52* (0.38,0.67)	0.47* (0.39,0.55)	0.47* (0.38,0.57)	0.60* (0.51,0.69)	0.62* (0.53,0.71)
Conditional height in infancy	0.05 (-0.02,0.12)	0.05. (-0.01,0.12)	-0.01 (-0.07,0.05)	0.06* (0.02,0.11)	0.35* (0.06,0.64)	0.16 (-0.10,0.41)	0.13* (0.04,0.23)	-0.05 (-0.19,0.10)	0.14* (0.07,0.20)	0.17* (0.09,0.24)	0.05 (-0.04,0.14)	0.20* (0.11,0.29)
Conditional height in childhood	-0.08 (-0.2,0.04)	-0.04 (-0.15,0.07)	0.03 (-0.06,0.12)	0.06 (0.00,0.12)	-0.11 (-0.53,0.3)	-0.39 (-0.94,0.15)	0.11 (-0.05,0.28)	-0.14 (-0.36,0.08)	0.16* (0.06,0.27)	0.24* (0.13,0.35)	-0.04 (-0.2,0.12)	0.06 (-0.1,0.22)
Conditional height in adolescence	0.02 (-0.08,0.11)	-0.01 (-0.1,0.09)	-0.08 (-0.18,0.02)	0.08 (-0.01,0.17)	0.23 (-0.19,0.66)	-0.04 (-0.56,0.48)	0.08 (-0.03,0.19)	-0.12 (-0.38,0.14)	-0.04 (-0.17,0.1)	-0.11 (-0.29,0.06)	-0.02 (-0.16,0.12)	0.13 (-0.01,0.28)

[1] Values are standardized linear regression coefficients (βs and 95% CIs). In adjusted analyses we controlled for birth characteristics (gestational age, birth order), maternal characteristics (height, age at childbirth, schooling, marital status), paternal characteristics (age, schooling), income and/or wealth index of child's household at birth, and age in adulthood. In women' models, we adjusted for age of menarche and teenage childbearing. Additionally, we controlled for maternal skin color in both Brazilian cohorts and adjusted for birth year and intervention group (village fixed effects) in Guatemala analysis. * P value < 0.05; SD = Standard Deviation

[2] We used birth weight as the anchor in all conditional models given that this measure was available across all study sites

For peer review only

Supplementary Table 6. Adjusted associations between weight at birth, conditional growth (height and relative weight) in infancy, childhood, and adolescence with adult fat mass/fat free mass ratio (FM/FFM, SD units), stratified by study site and sex[1]

	Brazil 1982		Brazil 1993		Guatemala		India		The Philippines		South Africa	
	Men (n = 344)	Women (n = 330)	Men (n = 375)	Women (n = 452)	Men (n = 67)	Women (n = 96)	Men (n = 426)	Women (n = 255)	Men (n = 656)	Women (n = 541)	Men (n = 287)	Women (n = 308)
**Conditional growth, z-scores[2]**												
Birth weight	0.01 (-0.05,0.07)	-0.05 (-0.14,0.03)	0.01 (-0.05,0.07)	0.07* (0.01,0.13)	0.18 (-0.12,0.47)	0.02 (-0.24,0.27)	0.15* (0.05,0.26)	0.18* (0.04,0.33)	0.08* (0.01,0.16)	0.12* (0.04,0.20)	-0.07 (-0.18,0.04)	-0.03 (-0.13,0.07)
Conditional relative weight in infancy	0.11* (0.02,0.2)	0.25* (0.11,0.39)	0.15* (0.06,0.24)	0.14* (0.05,0.24)	0.35 (-0.24,0.94)	-0.24 (-0.74,0.25)	0.13 (-0.01,0.27)	0.16 (-0.03,0.35)	0.21* (0.10,0.31)	0.32* (0.20,0.44)	0.20* (0.09,0.30)	0.21* (0.08,0.34)
Conditional relative weight in childhood	0.26* (0.13,0.4)	0.38* (0.19,0.57)	0.22* (0.13,0.3)	0.39* (0.28,0.49)	-0.16 (-1.05,0.72)	0.31 (-0.35,0.96)	0.26* (0.04,0.47)	-0.11 (-0.38,0.15)	0.52* (0.38,0.65)	0.69* (0.53,0.85)	0.24* (0.07,0.42)	0.19 (0.00,0.39)
Conditional relative weight in adolescence	0.30* (0.23,0.37)	0.50* (0.40,0.59)	0.30* (0.23,0.37)	0.45* (0.36,0.53)	-0.01 (-0.54,0.53)	0.32 (-0.11,0.75)	0.21* (0.11,0.3)	0.41* (0.25,0.57)	0.34* (0.25,0.43)	0.40* (0.30,0.51)	0.49* (0.39,0.59)	0.46* (0.36,0.56)
Conditional height in infancy	0.00 (-0.07,0.07)	0.16* (0.06,0.25)	0.05 (0,0.09)	0.04 (-0.03,0.1)	0.16 (-0.18,0.51)	-0.02 (-0.33,0.29)	0.06 (-0.04,0.16)	0.17* (0.01,0.32)	0.11* (0.04,0.19)	0.13* (0.05,0.21)	0.06 (-0.04,0.16)	0.04 (-0.06,0.15)
Conditional height in childhood	-0.03 (-0.15,0.08)	0.03 (-0.13,0.20)	0.12* (0.05,0.19)	0.13* (0.04,0.23)	-0.09 (-0.57,0.4)	0.24 (-0.42,0.91)	0.10 (-0.09,0.28)	0.18 (-0.06,0.43)	0.13* (0.02,0.25)	0.24* (0.12,0.36)	0.08 (-0.10,0.26)	0.17 (-0.02,0.35)
Conditional height in adolescence	0.05 (-0.05,0.14)	0.26* (0.12,0.41)	0.08* (0.01,0.16)	-0.01 (-0.14,0.12)	0.42 (-0.08,0.92)	-0.03 (-0.66,0.60)	-0.01 (-0.14,0.11)	0.07 (-0.22,0.35)	0.02 (-0.13,0.17)	-0.11 (-0.29,0.07)	-0.12 (-0.28,0.04)	-0.18* (-0.35,-0.01)

[1] Values are standardized linear regression coefficients (βs and 95% CIs). In adjusted analyses we controlled for birth characteristics (gestational age, birth order), maternal characteristics (height, age at childbirth, schooling, marital status), paternal characteristics (age, schooling), income and/or wealth index of child's household at birth, and age in adulthood. In women' models, we adjusted for age of menarche and teenage childbearing. Additionally, we controlled for maternal skin color in both Brazilian cohorts and adjusted for birth year and intervention group (village fixed effects) in Guatemala analysis. * P value < 0.05; SD = Standard Deviation

[2] We used birth weight as the anchor in all conditional models given that this measure was available across all study sites

For peer review only

Supplementary Table 7. Adjusted associations between weight at birth, conditional growth (height and relative weight) in infancy, childhood, and adolescence with adult body mass index (BMI, SD units), stratified by study site and sex[1]

	Brazil 1982		Brazil 1993		Guatemala		India		The Philippines		South Africa	
	Men (n = 344)	Women (n = 330)	Men (n = 375)	Women (n = 452)	Men (n = 67)	Women (n = 96)	Men (n = 426)	Women (n = 255)	Men (n = 656)	Women (n = 541)	Men (n = 287)	Women (n = 308)
**Conditional growth, z-scores[2]**												
Birth weight	0.08* (0.02,0.15)	0.02 (-0.07,0.10)	0.04 (-0.02,0.10)	0.13* (0.07,0.20)	0.03 (-0.24,0.29)	0.33** (0.12,0.55)	0.14* (0.04,0.23)	0.17* (0.03,0.30)	0.12* (0.04,0.19)	0.12* (0.04,0.20)	0.11* (0.02,0.20)	0.07 (-0.02,0.15)
Conditional relative weight in infancy	0.29* (0.19,0.39)	0.36* (0.21,0.5)	0.33* (0.23,0.42)	0.33* (0.24,0.42)	0.51 (-0.02,1.04)	0.07 (-0.36,0.5)	0.28* (0.15,0.41)	0.32* (0.14,0.50)	0.28* (0.18,0.38)	0.31* (0.19,0.43)	0.22* (0.14,0.31)	0.24* (0.13,0.35)
Conditional relative weight in childhood	0.53* (0.38,0.67)	0.59* (0.4,0.79)	0.47* (0.38,0.57)	0.62* (0.52,0.73)	0.19 (-0.61,0.99)	0.38 (-0.19,0.95)	0.44* (0.24,0.64)	0.17 (-0.08,0.42)	0.65* (0.53,0.78)	0.70* (0.54,0.85)	0.35* (0.21,0.49)	0.37* (0.2,0.53)
Conditional relative weight in adolescence	0.54* (0.46,0.62)	0.70* (0.6,0.79)	0.49* (0.42,0.55)	0.66* (0.55,0.77)	0.42. (-0.07,0.91)	0.57* (0.20,0.94)	0.35* (0.27,0.44)	0.54* (0.39,0.69)	0.43* (0.34,0.52)	0.44* (0.34,0.54)	0.67* (0.59,0.75)	0.63* (0.55,0.72)
Conditional height in infancy	0.04 (-0.04,0.11)	0.16* (0.06,0.26)	0.04 (-0.01,0.10)	0.08* (0.01,0.15)	0.35* (0.03,0.66)	0.14 (-0.13,0.41)	0.12* (0.03,0.21)	0.07 (-0.08,0.22)	0.13* (0.06,0.20)	0.14* (0.06,0.21)	0.06 (-0.02,0.14)	0.13* (0.04,0.22)
Conditional height in childhood	-0.10 (-0.23,0.03)	-0.02 (-0.19,0.15)	0.13* (0.05,0.21)	0.14* (0.04,0.25)	-0.13 (-0.57,0.31)	-0.19 (-0.76,0.39)	0.11 (-0.06,0.27)	0.04 (-0.2,0.27)	0.15* (0.04,0.26)	0.27* (0.15,0.39)	0.03 (-0.11,0.18)	0.14 (-0.02,0.29)
Conditional height in adolescence	0.06 (-0.04,0.16)	0.18* (0.03,0.33)	0.03 (-0.06,0.12)	0.07 (-0.06,0.19)	0.34 (-0.11,0.79)	-0.06 (-0.61,0.48)	0.03 (-0.08,0.14)	-0.01 (-0.28,0.26)	-0.03 (-0.17,0.11)	-0.09 (-0.27,0.08)	-0.09 (-0.21,0.04)	-0.03 (-0.17,0.11)

[1] Values are standardized linear regression coefficients (βs and 95% CIs). In adjusted analyses we controlled for birth characteristics (gestational age, birth order), maternal characteristics (height, age at childbirth, schooling, marital status), paternal characteristics (age, schooling), income and/or wealth index of child's household at birth, and age in adulthood. In women' models, we adjusted for age of menarche and teenage childbearing. Additionally, we controlled for maternal skin color in both Brazilian cohorts and adjusted for birth year and intervention group (village fixed effects) in Guatemala analysis. * P value < 0.05; SD = Standard Deviation

[2] We used birth weight as the anchor in all conditional models given that this measure was available across all study sites

For peer review only

Supplementary Table 8. Adjusted associations between weight at birth, conditional growth (height and relative weight) in infancy, childhood, and adolescence with adult waist circumference (SD units), stratified by study site and sex[1]

	Brazil 1982		Brazil 1993		Guatemala		India		The Philippines		South Africa	
	Men (n = 344)	Women (n = 330)	Men (n = 375)	Women (n = 452)	Men (n = 67)	Women (n = 96)	Men (n = 426)	Women (n = 255)	Men (n = 656)	Women (n = 541)	Men (n = 287)	Women (n = 308)
**Conditional growth, z-scores[2]**												
Birth weight	0.06 (-0.03,0.14)	0.03 (-0.07,0.13)	0.03 (-0.04,0.10)	0.11* (0.01,0.21)	0.03 (-0.26,0.32)	0.32* (0.05,0.58)	0.14* (0.03,0.24)	0.13 (-0.03,0.30)	0.09* (0.01,0.18)	0.08 (-0.02,0.18)	0.03 (-0.08,0.14)	0.01 (-0.10,0.12)
Conditional relative weight in infancy	0.26* (0.14,0.37)	0.27* (0.14,0.39)	0.33* (0.22,0.43)	0.30* (0.21,0.39)	0.51 (-0.05,1.08)	0.12 (-0.33,0.56)	0.23* (0.10,0.37)	0.31* (0.13,0.49)	0.25* (0.14,0.35)	0.25* (0.12,0.37)	0.17* (0.07,0.27)	0.17* (0.04,0.3)
Conditional relative weight in childhood	0.49* (0.32,0.66)	0.50* (0.32,0.67)	0.45* (0.33,0.57)	0.50* (0.38,0.62)	0.29 (-0.52,1.11)	0.17 (-0.4,0.73)	0.44* (0.24,0.65)	0.02 (-0.24,0.28)	0.47* (0.33,0.6)	0.57* (0.41,0.73)	0.28* (0.11,0.45)	0.23* (0.03,0.42)
Conditional relative weight in adolescence	0.53* (0.44,0.62)	0.52* (0.44,0.61)	0.45* (0.37,0.53)	0.58* (0.48,0.68)	0.38 (-0.16,0.91)	0.50* (0.13,0.87)	0.26* (0.16,0.35)	0.44* (0.28,0.6)	0.33* (0.23,0.42)	0.41* (0.30,0.51)	0.51* (0.41,0.61)	0.51* (0.41,0.60)
Conditional height in infancy	0.04 (-0.06,0.15)	0.22* (0.07,0.36)	0.08* (0.00,0.16)	0.08 (-0.05,0.21)	0.28 (-0.08,0.64)	-0.08 (-0.58,0.42)	0.11 (0.00,0.22)	-0.16 (-0.40,0.09)	0.22* (0.12,0.31)	0.11 (-0.06,0.27)	0.02 (-0.1,0.14)	0.10 (-0.06,0.26)
Conditional height in childhood	-0.06 (-0.22,0.1)	0.10 (-0.11,0.31)	0.19* (0.08,0.3)	0.19 (-0.01,0.38)	-0.19 (-0.66,0.28)	-0.22 (-0.85,0.42)	0.12 (-0.06,0.3)	0.12 (-0.22,0.46)	0.23* (0.09,0.38)	0.20 (-0.04,0.43)	0.00 (-0.21,0.21)	0.15 (-0.10,0.4)
Conditional height in adolescence	0.06 (-0.06,0.19)	0.26* (0.03,0.49)	0.10 (0.00,0.21)	0.07 (-0.16,0.31)	0.28 (-0.34,0.89)	-0.38 (-1.38,0.63)	0.06 (-0.06,0.19)	-0.17 (-0.61,0.27)	0.13 (-0.05,0.31)	-0.13 (-0.49,0.22)	-0.07 (-0.26,0.11)	-0.15 (-0.43,0.13)

[1] Values are standardized linear regression coefficients (βs and 95% CIs). In adjusted analyses we controlled for birth characteristics (gestational age, birth order), maternal characteristics (height, age at childbirth, schooling, marital status), paternal characteristics (age, schooling), income and/or wealth index of child's household at birth, and age and height in adulthood. In women' models, we adjusted for age of menarche and teenage childbearing. Additionally, we controlled for maternal skin color in both Brazilian cohorts and adjusted for birth year and intervention group (village fixed effects) in Guatemala analysis. * P value < 0.05; SD = Standard Deviation

[2] We used birth weight as the anchor in all conditional models given that this measure was available across all study sites

For peer review only

Supplementary Table 9. Pooled adjusted associations between length at birth, conditional growth (height and relative weight) in infancy, childhood, and adolescence with adult fat mass and fat-free mass indices, stratified by sex[1]

	FMI (SD units)						FFMI (SD units)					
	Men (n = 1524)			Women (n = 1344)			Men (n = 1524)			Women (n = 1344)		
	β (95%CI)	I^2 statistic (%)[3]	Cochran's Q test (P value)	β (95%CI)	I^2 statistic (%)	Cochran's Q test (P value)	β (95%CI)	I^2 statistic (%)	Cochran's Q test (P value)	β (95%CI)	I^2 statistic (%)	Cochran's Q test (P value)
**Conditional growth, z-scores[2]**												
Birth length	0.06 (-0.03,0.15)	50.2	0.128	0.09 (-0.01,0.20)	46.4	0.165	0.06 (-0.02,0.15)	54.8	0.083	0.07 (0.00,0.14)	7.4	0.325
Conditional height in infancy	0.11* (0.04,0.19)	2.5	0.378	0.11* (0.04,0.18)	16.7	0.539	0.12 (-0.06,0.3)	76.4	0.004	0.10 (-0.07,0.27)	78.8	0.006
Conditional height in childhood	0.11* (0.03,0.20)	0.0	0.714	0.16* (0.03,0.30)	20.2	0.425	0.11 (-0.04,0.25)	41.1	0.175	0.06 (-0.32,0.45)	88.2	0.001
Conditional height in adolescence	0.03 (-0.09,0.16)	0.0	0.402	-0.03 (-0.14,0.07)	0.0	0.768	0.00 (-0.17,0.17)	47.7	0.113	-0.04 (-0.22,0.14)	44.1	0.194
Conditional relative weight at birth	0.09* (0.04,0.14)	0.0	0.870	0.11* (0.03,0.20)	0.0	0.556	0.10* (0.02,0.18)	0.0	0.527	0.14 (-0.05,0.33)	52.8	0.070
Conditional relative weight in infancy	0.23* (0.17,0.30)	0.0	0.819	0.25* (0.11,0.39)	8.9	0.297	0.32* (0.27,0.37)	0.0	0.871	0.34* (0.24,0.44)	8.1	0.511
Conditional relative weight in childhood	0.43* (0.17,0.70)	71.1	0.009	0.49 (-0.03,1.03)	90	<0.001	0.55* (0.3,0.81)	75.7	0.003	0.50* (0.19,0.82)	76.7	0.005
Conditional relative weight in adolescence	0.34* (0.24,0.44)	29.3	0.282	0.49* (0.37,0.60)	34.5	0.296	0.41* (0.29,0.54)	56	0.090	0.45* (0.34,0.56)	43.2	0.206

[1] Values are standardized linear regression coefficients (*β*s and 95% CIs). FMI, fat mass index; FFMI, fat-free mass index. In adjusted analyses we controlled for birth characteristics (gestational age, birth order), maternal characteristics (height, age at childbirth, schooling, marital status), paternal schooling, income and/or wealth index of child's household at birth, and age in adulthood. In women' models, we adjusted for age of menarche and teenage childbearing. Additionally, we controlled for maternal skin color in Brazil and adjusted for birth year and intervention group (village fixed effects) in Guatemala analysis. * P value < 0.05
[2] We used birth length as the anchor in conditional models of sites that collected this measure (Brazil 1993, Guatemala, India, The Philippines)
[3] As a rule of thumb I^2 = 25% is interpreted as low heterogeneity, 50% moderate heterogeneity, and 75% substantial heterogeneity

For peer review only

Supplementary Table 10. Pooled adjusted associations between length at birth, conditional growth (height and relative weight) in infancy, childhood, and adolescence with adult body mass index and waist circumference, stratified by sex[1]

	BMI (SD units)						Waist circumference (SD units)					
	Men (n = 1524)			Women (n = 1344)			Men (n = 1524)			Women (n = 1344)		
	β (95%CI)	I^2 statistic (%)[3]	Cochran's Q test (P value)	β (95%CI)	I^2 statistic (%)	Cochran's Q test (P value)	β (95%CI)	I^2 statistic (%)	Cochran's Q test (P value)	β (95%CI)	I^2 statistic (%)	Cochran's Q test (P value)
**Conditional growth, z-scores[2]**												
Birth length	0.06 (-0.03,0.16)	57.5	0.070	0.10* (0.03,0.17)	0.9	0.389	0.06 (-0.04,0.17)	50.1	0.116	0.06 (-0.01,0.12)	0.0	0.739
Conditional height in infancy	0.12* (0.02,0.23)	29.9	0.149	0.11* (0.04,0.18)	1.7	0.540	0.16* (0.05,0.28)	29.4	0.258	0.05 (-0.15,0.24)	29.1	0.255
Conditional height in childhood	0.12* (0.04,0.21)	0.2	0.651	0.15 (-0.07,0.37)	53.8	0.101	0.18* (0.02,0.33)	0.1	0.309	0.15* (0.00,0.3)	0.0	0.725
Conditional height in adolescence	0.02 (-0.09,0.13)	0.1	0.503	-0.03 (-0.16,0.11)	11.1	0.573	0.11* (0.04,0.18)	0.0	0.871	-0.09 (-0.33,0.15)	0.0	0.631
Conditional relative weight at birth	0.10* (0.07,0.13)	0.0	0.955	0.13 (0.00,0.26)	0.0	0.236	0.08* (0.02,0.14)	0.0	0.808	0.13 (-0.01,0.28)	0.1	0.166
Conditional relative weight in infancy	0.30* (0.24,0.35)	0.0	0.840	0.30* (0.22,0.38)	0.0	0.700	0.27* (0.19,0.35)	0.0	0.642	0.26* (0.19,0.34)	0.0	0.752
Conditional relative weight in childhood	0.53* (0.33,0.73)	54.6	0.095	0.55* (0.11,0.98)	86	0.001	0.45* (0.41,0.49)	0.0	0.982	0.41 (-0.04,0.86)	85.5	0.002
Conditional relative weight in adolescence	0.42* (0.31,0.53)	55.4	0.109	0.54* (0.37,0.71)	63.8	0.030	0.34* (0.19,0.49)	68.8	0.023	0.48* (0.33,0.62)	51.5	0.114

[1] Values are standardized linear regression coefficients (*β*s and 95% CIs). BMI, body mass index. In adjusted analyses we controlled for birth characteristics (gestational age, birth order), maternal characteristics (height, age at childbirth, schooling, marital status), paternal schooling, income and/or wealth index of child's household at birth, and age in adulthood. In women' models, we adjusted for age of menarche and teenage childbearing. Additionally, we controlled for maternal skin color in Brazil and adjusted for birth year and intervention group (village fixed effects) in Guatemala analysis. * P value < 0.05

[2] We used birth length as the anchor in conditional models of sites that collected this measure (Brazil 1993, Guatemala, India, The Philippines)

[3] As a rule of thumb I^2 = 25% is interpreted as low heterogeneity, 50% moderate heterogeneity, and 75% substantial heterogeneity

For peer review only

Supplementary Table 11. Adjusted associations between length at birth, conditional growth (height and relative weight) in infancy, childhood, and adolescence with adult fat mass index (FMI, SD units), stratified by study site and sex[1]

	Brazil 1982		Brazil 1993		Guatemala		India		The Philippines		South Africa	
	Men (n = 344)	Women (n = 330)	Men (n = 375)	Women (n = 452)	Men (n = 67)	Women (n = 96)	Men (n = 426)	Women (n = 255)	Men (n = 656)	Women (n = 541)	Men (n = 287)	Women (n = 308)
**Conditional growth, z-scores**[2]												
Birth length	NA	NA	-0.01 (-0.06,0.05)	0.07* (0.01,0.14)	0.00 (-0.23,0.23)	0.06 (-0.15,0.28)	0.11* (0.02,0.20)	0.21* (0.10,0.32)	0.07* (0.01,0.14)	0.07 (0.00,0.14)	NA	NA
Conditional height in infancy	NA	NA	0.07* (0.02,0.13)	0.07* (0,0.14)	0.31 (-0.04,0.66)	0.11 (-0.2,0.42)	0.09 (-0.01,0.2)	0.09 (-0.06,0.25)	0.13* (0.06,0.20)	0.15* (0.07,0.22)	NA	NA
Conditional height in childhood	NA	NA	0.14* (0.06,0.22)	0.14* (0.03,0.24)	-0.12 (-0.59,0.35)	0.02 (-0.62,0.65)	0.10 (-0.08,0.27)	0.09 (-0.15,0.33)	0.14* (0.03,0.25)	0.25* (0.13,0.37)	NA	NA
Conditional height in adolescence	NA	NA	0.07 (-0.01,0.16)	0.03 (-0.1,0.15)	0.35 (-0.14,0.84)	-0.05 (-0.65,0.55)	0.00 (-0.11,0.12)	-0.02 (-0.30,0.26)	0.00 (-0.15,0.14)	-0.09 (-0.27,0.09)	NA	NA
Conditional relative weight at birth	NA	NA	0.07 (-0.03,0.17)	0.12* (0.02,0.22)	0.14 (-0.18,0.47)	0.23 (-0.05,0.51)	0.13 (0.00,0.27)	0.00 (-0.19,0.19)	0.08 (-0.02,0.17)	0.12* (0.02,0.22)	NA	NA
Conditional relative weight in infancy	NA	NA	0.21* (0.11,0.31)	0.22* (0.13,0.31)	0.42 (-0.16,1.01)	-0.06 (-0.53,0.41)	0.20* (0.06,0.34)	0.26* (0.07,0.44)	0.25* (0.15,0.36)	0.33* (0.21,0.45)	NA	NA
Conditional relative weight in childhood	NA	NA	0.32* (0.22,0.42)	0.50* (0.40,0.61)	0.04 (-0.82,0.89)	0.45 (-0.18,1.07)	0.36* (0.15,0.57)	0.01 (-0.25,0.26)	0.59* (0.46,0.72)	0.72* (0.56,0.88)	NA	NA
Conditional relative weight in adolescence	NA	NA	0.38* (0.30,0.45)	0.56* (0.45,0.66)	0.16 (-0.36,0.69)	0.53* (0.13,0.94)	0.28* (0.19,0.37)	0.50* (0.35,0.65)	0.38* (0.29,0.47)	0.42* (0.32,0.52)	NA	NA

[1] Values are standardized linear regression coefficients (βs and 95% CIs). In adjusted analyses we controlled for birth characteristics (gestational age, birth order), maternal characteristics (height, age at childbirth, schooling, marital status), paternal characteristics (age, schooling), income and/or wealth index of child's household at birth, and age in adulthood. In women' models, we adjusted for age of menarche and teenage childbearing. Additionally, we controlled for maternal skin color in Brazil and adjusted for birth year and intervention group (village fixed effects) in Guatemala analysis. * P value < 0.05; SD = Standard Deviation
[2] We used birth length as the anchor in conditional models of sites that collected this measure (Brazil 1993, Guatemala, India, The Philippines)

For peer review only

Supplementary Table 12. Adjusted associations between length at birth, conditional growth (height and relative weight) in infancy, childhood, and adolescence with adult fat-free mass index (FFMI, SD units), stratified by study site and sex[1]

	Brazil 1982		Brazil 1993		Guatemala		India		The Philippines		South Africa	
	Men (n = 344)	Women (n = 330)	Men (n = 375)	Women (n = 452)	Men (n = 67)	Women (n = 96)	Men (n = 426)	Women (n = 255)	Men (n = 656)	Women (n = 541)	Men (n = 287)	Women (n = 308)
**Conditional growth, z-scores[2]**												
Birth length	NA	NA	0.00 (-0.05,0.05)	0.08* (0.04,0.12)	0.00 (-0.20,0.20)	0.19 (-0.01,0.38)	0.09* (0.01,0.18)	0.09 (-0.02,0.19)	0.09* (0.03,0.15)	0.03 (-0.04,0.09)	NA	NA
Conditional height in infancy	NA	NA	0.01 (-0.05,0.07)	0.06* (0.02,0.11)	0.35* (0.05,0.66)	0.16 (-0.11,0.43)	0.13* (0.04,0.23)	-0.05 (-0.20,0.09)	0.14* (0.08,0.21)	0.19* (0.12,0.26)	NA	NA
Conditional height in childhood	NA	NA	0.03 (-0.06,0.12)	0.06 (0.00,0.12)	-0.10 (-0.52,0.31)	-0.40 (-0.95,0.16)	0.11 (-0.05,0.28)	-0.15 (-0.37,0.07)	0.17* (0.06,0.27)	0.24* (0.13,0.35)	NA	NA
Conditional height in adolescence	NA	NA	-0.08 (-0.18,0.02)	0.08 (-0.01,0.17)	0.25 (-0.19,0.68)	-0.05 (-0.58,0.48)	0.08 (-0.03,0.19)	-0.14 (-0.40,0.13)	-0.03 (-0.17,0.1)	-0.09 (-0.26,0.08)	NA	NA
Conditional relative weight at birth	NA	NA	0.13* (0.05,0.21)	0.09* (0.02,0.16)	-0.08 (-0.37,0.21)	0.42* (0.18,0.67)	0.08 (-0.05,0.21)	0.10 (-0.08,0.28)	0.11* (0.02,0.20)	0.14* (0.04,0.24)	NA	NA
Conditional relative weight in infancy	NA	NA	0.29* (0.21,0.37)	0.29* (0.23,0.36)	0.46 (-0.06,0.98)	0.22 (-0.19,0.63)	0.34* (0.21,0.46)	0.40* (0.23,0.58)	0.31* (0.21,0.41)	0.37* (0.25,0.49)	NA	NA
Conditional relative weight in childhood	NA	NA	0.43* (0.33,0.52)	0.41* (0.33,0.5)	0.33 (-0.43,1.10)	0.21 (-0.34,0.75)	0.46* (0.26,0.65)	0.36* (0.12,0.60)	0.71* (0.59,0.83)	0.70* (0.55,0.85)	NA	NA
Conditional relative weight in adolescence	NA	NA	0.34* (0.28,0.41)	0.38* (0.32,0.44)	0.59* (0.12,1.06)	0.45* (0.10,0.81)	0.36* (0.28,0.44)	0.52* (0.38,0.67)	0.47* (0.39,0.55)	0.47* (0.38,0.57)	NA	NA

[1] Values are standardized linear regression coefficients (βs and 95% CIs). In adjusted analyses we controlled for birth characteristics (gestational age, birth order), maternal characteristics (height, age at childbirth, schooling, marital status), paternal characteristics (age, schooling), income and/or wealth index of child's household at birth, and age in adulthood. In women' models, we adjusted for age of menarche and teenage childbearing. Additionally, we controlled for maternal skin color in Brazil and adjusted for birth year and intervention group (village fixed effects) in Guatemala analysis. * P value < 0.05; SD = Standard Deviation

[2] We used birth length as the anchor in conditional models of sites that collected this measure (Brazil 1993, Guatemala, India, The Philippines)

For peer review only

Supplementary Table 13. Adjusted associations length at birth, conditional growth (height and relative weight) in infancy, childhood, and adolescence with adult body mass index (BMI, SD units), stratified by study site and sex[1]

	Brazil 1982		Brazil 1993		Guatemala		India		The Philippines		South Africa	
	Men (n = 344)	Women (n = 330)	Men (n = 375)	Women (n = 452)	Men (n = 67)	Women (n = 96)	Men (n = 426)	Women (n = 255)	Men (n = 656)	Women (n = 541)	Men (n = 287)	Women (n = 308)
**Conditional growth, z-scores[2]**												
Birth length	NA	NA	-0.01 (-0.06,0.05)	0.10* (0.04,0.16)	0.00 (-0.22,0.22)	0.13 (-0.07,0.33)	0.10* (0.02,0.19)	0.18* (0.07,0.29)	0.08* (0.02,0.15)	0.07 (0.00,0.14)	NA	NA
Conditional height in infancy	NA	NA	0.06* (0.01,0.12)	0.09* (0.02,0.16)	0.37* (0.04,0.70)	0.15 (-0.13,0.44)	0.12* (0.02,0.21)	0.04 (-0.11,0.20)	0.14* (0.07,0.20)	0.15* (0.07,0.23)	NA	NA
Conditional height in childhood	NA	NA	0.13* (0.05,0.21)	0.14* (0.04,0.25)	-0.13 (-0.57,0.32)	-0.18 (-0.76,0.41)	0.11 (-0.06,0.27)	0.01 (-0.22,0.25)	0.15* (0.05,0.26)	0.27* (0.15,0.39)	NA	NA
Conditional height in adolescence	NA	NA	0.03 (-0.06,0.11)	0.06 (-0.06,0.19)	0.33 (-0.13,0.8)	-0.06 (-0.61,0.5)	0.03 (-0.08,0.15)	-0.05 (-0.32,0.22)	-0.03 (-0.17,0.11)	-0.08 (-0.26,0.09)	NA	NA
Conditional relative weight at birth	NA	NA	0.12* (0.02,0.21)	0.15* (0.04,0.25)	0.04 (-0.27,0.34)	0.36* (0.1,0.61)	0.11 (-0.02,0.24)	0.04 (-0.15,0.22)	0.09* (0.00,0.19)	0.12* (0.02,0.22)	NA	NA
Conditional relative weight in infancy	NA	NA	0.32* (0.22,0.41)	0.33* (0.24,0.42)	0.49 (-0.07,1.04)	0.06 (-0.37,0.50)	0.28* (0.15,0.41)	0.33* (0.15,0.51)	0.28* (0.18,0.38)	0.31* (0.19,0.43)	NA	NA
Conditional relative weight in childhood	NA	NA	0.47* (0.38,0.57)	0.63* (0.52,0.73)	0.20 (-0.61,1.01)	0.40 (-0.18,0.97)	0.44* (0.24,0.64)	0.14 (-0.11,0.39)	0.65* (0.53,0.78)	0.70* (0.54,0.85)	NA	NA
Conditional relative weight in adolescence	NA	NA	0.49* (0.42,0.55)	0.66* (0.55,0.77)	0.41 (-0.09,0.91)	0.57* (0.19,0.95)	0.35* (0.27,0.44)	0.54* (0.39,0.69)	0.43* (0.34,0.52)	0.44* (0.34,0.54)	NA	NA

[1] Values are standardized linear regression coefficients (βs and 95% CIs). In adjusted analyses we controlled for birth characteristics (gestational age, birth order), maternal characteristics (height, age at childbirth, schooling, marital status), paternal characteristics (age, schooling), income and/or wealth index of child's household at birth, and age in adulthood. In women' models, we adjusted for age of menarche and teenage childbearing. Additionally, we controlled for maternal skin color in Brazil and adjusted for birth year and intervention group (village fixed effects) in Guatemala analysis. * P value < 0.05; SD = Standard Deviation

[2] We used birth length as the anchor in conditional models of sites that collected this measure (Brazil 1993, Guatemala, India, The Philippines)

For peer review only

Supplementary Table 14. Adjusted associations between length at birth, conditional growth (height and relative weight) in infancy, childhood, and adolescence with adult waist circumference (SD units), stratified by study site and sex

	Brazil 1982		Brazil 1993		Guatemala		India		The Philippines		South Africa	
	Men (n = 344)	Women (n = 330)	Men (n = 375)	Women (n = 452)	Men (n = 67)	Women (n = 96)	Men (n = 426)	Women (n = 255)	Men (n = 656)	Women (n = 541)	Men (n = 287)	Women (n = 308)
**Conditional growth, z-scores**[2]												
Birth length	NA	NA	-0.01 (-0.07,0.05)	0.08 (-0.03,0.19)	-0.03 (-0.26,0.21)	0.03 (-0.22,0.28)	0.10* (0.00,0.20)	0.11 (-0.05,0.26)	0.09* (0.02,0.17)	0.02 (-0.09,0.12)	NA	NA
Conditional height in infancy	NA	NA	0.11* (0.03,0.19)	0.09 (-0.04,0.22)	0.32 (-0.06,0.69)	-0.03 (-0.53,0.47)	0.11 (0.00,0.23)	-0.16 (-0.40,0.08)	0.22* (0.12,0.31)	0.12 (-0.04,0.28)	NA	NA
Conditional height in childhood	NA	NA	0.19* (0.08,0.3)	0.18 (-0.01,0.38)	-0.19 (-0.66,0.28)	-0.18 (-0.82,0.46)	0.12 (-0.06,0.31)	0.12 (-0.22,0.46)	0.24* (0.09,0.38)	0.20 (-0.04,0.43)	NA	NA
Conditional height in adolescence	NA	NA	0.10 (-0.01,0.2)	0.06 (-0.17,0.29)	0.27 (-0.35,0.88)	-0.34 (-1.36,0.67)	0.07 (-0.06,0.19)	-0.18 (-0.62,0.26)	0.13 (-0.05,0.31)	-0.12 (-0.48,0.23)	NA	NA
Conditional relative weight at birth	NA	NA	0.10 (-0.01,0.21)	0.12* (0.02,0.23)	0.07 (-0.25,0.39)	0.42* (0.15,0.68)	0.11 (-0.02,0.25)	0.10 (-0.09,0.29)	0.04 (-0.05,0.14)	0.10 (0.00,0.21)	NA	NA
Conditional relative weight in infancy	NA	NA	0.32* (0.21,0.42)	0.30* (0.21,0.39)	0.47 (-0.12,1.06)	0.10 (-0.34,0.55)	0.23* (0.10,0.37)	0.31* (0.13,0.50)	0.25* (0.14,0.35)	0.24* (0.12,0.37)	NA	NA
Conditional relative weight in childhood	NA	NA	0.45* (0.33,0.57)	0.50* (0.39,0.62)	0.31 (-0.51,1.13)	0.19 (-0.38,0.76)	0.44* (0.24,0.65)	0.00 (-0.26,0.26)	0.47* (0.33,0.60)	0.56* (0.41,0.72)	NA	NA
Conditional relative weight in adolescence	NA	NA	0.45* (0.37,0.52)	0.58* (0.48,0.68)	0.35 (-0.20,0.90)	0.50* (0.13,0.87)	0.26* (0.16,0.35)	0.43* (0.27,0.60)	0.33* (0.23,0.42)	0.40* (0.30,0.51)	NA	NA

[1] Values are standardized linear regression coefficients (*β*s and 95% CIs). In adjusted analyses we controlled for birth characteristics (gestational age, birth order), maternal characteristics (height, age at childbirth, schooling, marital status), paternal characteristics (age, schooling), income and/or wealth index of child's household at birth, and age and height in adulthood. In women' models, we adjusted for age of menarche and teenage childbearing. Additionally, we controlled for maternal skin color in Brazil and adjusted for birth year and intervention group (village fixed effects) in Guatemala analysis. * P value < 0.05; SD = Standard Deviation

[2] We used birth length as the anchor in conditional models of sites that collected this measure (Brazil 1993, Guatemala, India, The Philip

For peer review only

Supplementary Table 15. Correlations between adult height and body composition measurements, stratified by study site and sex

	Brazil 1982		Brazil 1993		Guatemala		India		The Philippines		South Africa	
	r	p-value	r	p-value	r	p-value	r	p-value	r	p-value	r	p-value
**Men**												
BMI	0.00	0.944	0.02	0.744	0.01	0.935	-0.01	0.904	0.06	0.103	0.01	0.830
FMI	-0.01	0.888	0.04	0.450	0.10	0.445	0.02	0.704	0.05	0.160	0.04	0.504
FFMI	0.00	0.944	-0.02	0.682	-0.08	0.509	-0.01	0.851	0.07	0.059	0.01	0.872
WC	0.17	0.001	0.17	0.001	0.17	0.163	0.18	<0.001	0.21	<0.001	0.26	<0.001
**Women**												
BMI	-0.04	0.479	-0.09	0.065	0.03	0.806	0.01	0.888	0.05	0.246	-0.06	0.258
FMI	-0.02	0.749	-0.09	0.052	0.04	0.676	0.09	0.167	0.04	0.322	-0.07	0.219
FFMI	-0.08	0.131	-0.05	0.294	-0.01	0.945	-0.15	0.014	0.06	0.157	-0.01	0.794
WC	0.08	0.133	0.06	0.194	0.22	0.03	0.23	<0.001	0.18	<0.001	0.15	0.008

BMI: body mass index; FMI: fat mass index; FFMI: fat-free mass index; WC: waist circumference

For peer review only

STROBE Statement—Checklist of items that should be included in reports of ***cohort studies***

	Item No	Recommendation	Reported on page
**Title and abstract**	1	(*a*) Indicate the study's design with a commonly used term in the title or the abstract	1
		(*b*) Provide in the abstract an informative and balanced summary of what was done and what was found	4
**Introduction**			
Background/rationale	2	Explain the scientific background and rationale for the investigation being reported	6-7
Objectives	3	State specific objectives, including any prespecified hypotheses	7
**Methods**			
Study design	4	Present key elements of study design early in the paper	8
Setting	5	Describe the setting, locations, and relevant dates, including periods of recruitment, exposure, follow-up, and data collection	8
Participants	6	(*a*) Give the eligibility criteria, and the sources and methods of selection of participants. Describe methods of follow-up	8
		(*b*) For matched studies, give matching criteria and number of exposed and unexposed	N/A
Variables	7	Clearly define all outcomes, exposures, predictors, potential confounders, and effect modifiers. Give diagnostic criteria, if applicable	8-10
Data sources/ measurement	8*	For each variable of interest, give sources of data and details of methods of assessment (measurement). Describe comparability of assessment methods if there is more than one group	8, 10-11
Bias	9	Describe any efforts to address potential sources of bias	12-13
Study size	10	Explain how the study size was arrived at	Figure 1
Quantitative variables	11	Explain how quantitative variables were handled in the analyses. If applicable, describe which groupings were chosen and why	6-7
Statistical methods	12	(*a*) Describe all statistical methods, including those used to control for confounding	11-13
		(*b*) Describe any methods used to examine subgroups and interactions	11-13
		(*c*) Explain how missing data were addressed	13
		(*d*) If applicable, explain how loss to follow-up was addressed	N/A
		(*e*) Describe any sensitivity analyses	11
**Results**			
Participants	13*	(a) Report numbers of individuals at each stage of study—eg numbers potentially eligible, examined for eligibility, confirmed eligible, included in the study, completing follow-up, and analysed	13-14
		(b) Give reasons for non-participation at each stage	8
		(c) Consider use of a flow diagram	Figure 1, 14
Descriptive data	14*	(a) Give characteristics of study participants (eg demographic, clinical, social) and information on exposures and potential confounders	13 & Tables 1 and 2
		(b) Indicate number of participants with missing data for each variable of interest	Suppl. Table 1
		(c) Summarise follow-up time (eg, average and total amount)	N/A

For peer review only

For peer review only - http://bmjopen.bmj.com/site/about/guidelines.xhtml

Outcome data	15*	Report numbers of outcome events or summary measures over time	Table 2
Main results	16	(*a*) Give unadjusted estimates and, if applicable, confounder-adjusted estimates and their precision (eg, 95% confidence interval). Make clear which confounders were adjusted for and why they were included	16-21 & Tables 3-4
		(*b*) Report category boundaries when continuous variables were categorized	N/A
		(*c*) If relevant, consider translating estimates of relative risk into absolute risk for a meaningful time period	N/A
Other analyses	17	Report other analyses done—eg analyses of subgroups and interactions, and sensitivity analyses	16-21 & Suppl. Tables
**Discussion**			
Key results	18	Summarise key results with reference to study objectives	22
Limitations	19	Discuss limitations of the study, taking into account sources of potential bias or imprecision. Discuss both direction and magnitude of any potential bias	24-25
Interpretation	20	Give a cautious overall interpretation of results considering objectives, limitations, multiplicity of analyses, results from similar studies, and other relevant evidence	22-24
Generalisability	21	Discuss the generalisability (external validity) of the study results	25
**Other information**			
Funding	22	Give the source of funding and the role of the funders for the present study and, if applicable, for the original study on which the present article is based	26

*Give information separately for exposed and unexposed groups.

Note: An Explanation and Elaboration article discusses each checklist item and gives methodological background and published examples of transparent reporting. The STROBE checklist is best used in conjunction with this article (freely available on the Web sites of PLoS Medicine at http://www.plosmedicine.org/, Annals of Internal Medicine at http://www.annals.org/, and Epidemiology at http://www.epidem.com/). Information on the STROBE Initiative is available at http://www.strobe-statement.org.

For peer review only
